# Next Generation SICLOPPS Screening for the Identification of Inhibitors of the HIF-1α/HIF-1β Protein–Protein Interaction

**DOI:** 10.1021/acschembio.4c00494

**Published:** 2024-09-23

**Authors:** Alexander McDermott, Leonie M. Windeln, Jacob S. D. Valentine, Leonardo Baldassarre, Andrew D. Foster, Ali Tavassoli

**Affiliations:** † School of Chemistry, University of Southampton, Southampton SO17 1BJ, U.K.; ‡ Curve Therapeutics, Delta House, Southampton Science Park, Southampton SO16 7NS, U.K.

## Abstract

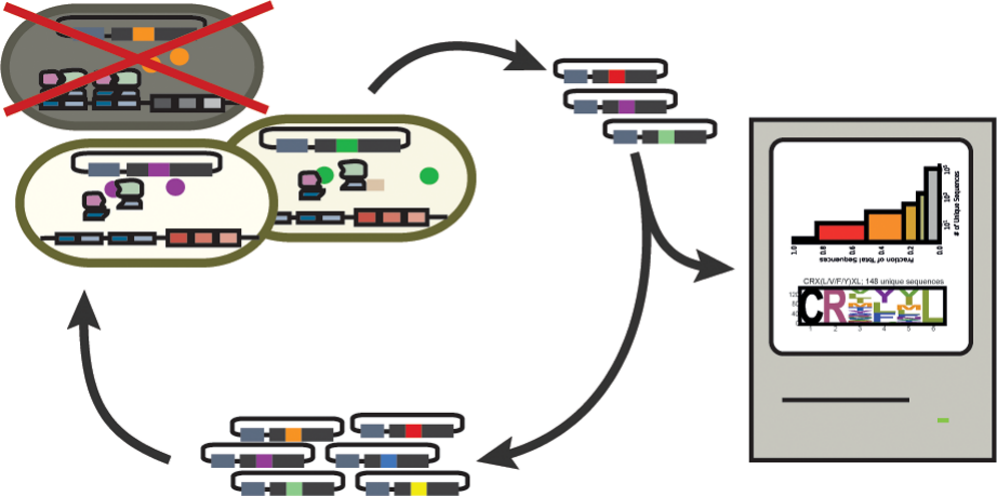

Split-intein circular ligation of proteins and peptides (SICLOPPS) is a method for generating intracellular libraries of cyclic peptides that has yielded several first-in-class inhibitors. Here, we detail a revised high-content, high-throughput SICLOPPS screening protocol that utilizes next-generation sequencing, biopanning, and computational tools to identify hits against a given protein–protein interaction. We used this platform for the identification of inhibitors of the HIF-1α/HIF-1β protein–protein interaction. The revised platform resulted in a significantly higher positive hit rate than that previously reported for SICLOPPS screens, and the identified cyclic peptides were more active in vitro and in cells than our previously reported inhibitors. The platform detailed here may be used for the identification of inhibitors of a wide range of other targets.

## Introduction

The advent of next-generation sequencing (NGS) has significantly aided drug discovery, enabling deep sequencing of potential hits from DNA-encoded libraries, phage display, and RNA display libraries.^1−3^ There are significant advantages in using NGS to deconvolute hits from genetically encoded libraries; naïve sequences, enriched sequences, and less prevalent individual sequences can be readily identified due to the immense throughput capability of modern NGS.^1,2,4−7^ Split-intein circular ligation of peptides and proteins (SICLOPPS) is a genetically encoded method that uses split-inteins to generate head-to-tail cyclic peptide libraries in cells.^8^ SICLOPPS libraries can be combined with high-throughput cell-based assays to enable the identification of cyclic peptide modulators of a given protein or biological process. The ability to generate and screen large libraries in live cells differentiates SICLOPPS from affinity-based in vitro screening approaches (e.g., DNA-encoded libraries, phage display, and mRNA display),^9^ enabling functional screening against proteins in their native intracellular environment where protein conformation is dynamic, and allosteric or cryptic pockets are exposed (c.f. proteins on a solid surface in biochemical buffer). In addition, cyclic peptides used in SICLOPPS screens are of typically 6 amino acids, whereas in affinity screening they tend to be between 12 and 20 amino acids, making translation to the clinic more challenging. SICLOPPS libraries have been used in combination with a bacterial reverse two-hybrid system (RTHS) for the identification of inhibitors of a variety of protein–protein interactions (PPI).^9^ This approach, which relies on visual identification and manual picking of surviving RTHS for hit selection, has led to a number of first-in-class inhibitors of a variety of PPIs.^9^ However, the manual hit selection process has the potential to introduce bias and errors and is not amenable to hit-enrichment methods such as biopanning used in both phage display and mRNA display. SICLOPPS libraries have also been used in cell-based assays with fluorescent reporters, using fluorescence-activated cell sorting (FACS) to separate *E. coli*-containing hits from the population.^10^

**Figure 1:**
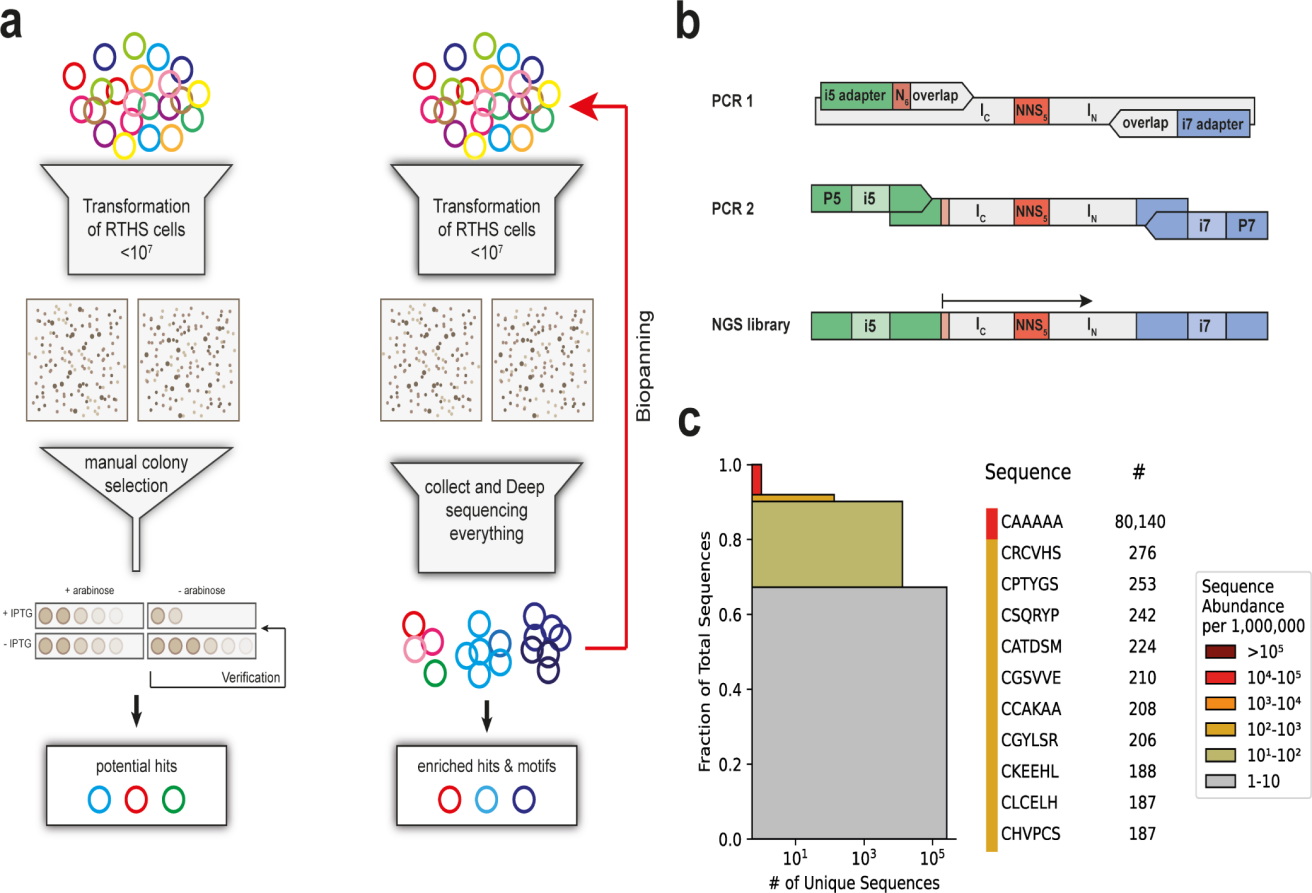
SICLOPPS NGS workflow and library preparation. a) The previous manual screening workflow (left) involved picking the largest surviving colonies by hand and subjecting each colony to further assessment for growth advantage by drop spotting; in the revised workflow (right), the screening plate is scraped to collect all surviving colonies, with rounds of biopanning to enrich hits, and the copy number in NGS used to rank hits. b) Schematic overview of the preparation of an Illumina-compatible library; in PCR 1, the universal i5 and i7 sequencing adapters are added to the C-intein (I_C_) and N-intein (I_N_) as well as N_6_ (NNNNNN) upstream of the complementary region to the I_C_. The second PCR (barcoding PCR) is used to attach unique barcodes i5 and i7 as well as flow cell binding sequences P5 and P7. Sequencing starts immediately after the i5 adapter is attached on the forward strand. c) The abundance of sequences in the initial library as visualized by a sequence redundancy plot; each colored box represents all sequences with the respective abundance of unique sequences per 1M; the number of unique sequences with the same abundance is on the *x*-axis.

Here, we report a revised method and workflow for SICLOPPS screening that uses pooled colony collection, NGS, and biopanning, which we postulate would enable a more accurate screen with fewer false positives. To illustrate the new approach, we use a previously reported hypoxia-inducible factor 1 (HIF-1) RTHS to identify inhibitors of the HIF-1α/HIF-1β PPI. The HIF-1 transcription factor plays a critical role in the adaptation and survival of solid tumors to their hypoxic microenvironment by altering the transcription of over a hundred genes.^11^ Consequently, genes involved in cell proliferation, angiogenesis, and metastasis are upregulated, promoting aggressive cancer phenotypes that are often resistant to therapeutic intervention, leading to poor patient prognosis.^12^ Inhibition of this transcription factor has been a longstanding target of drug discovery, with a potentially significant impact on a variety of cancers.^13^ We have previously used SICLOPPS screening to identify a specific inhibitor of the HIF-1α/HIF-1β PPI (*cyclo*-CLLFVY), an isoform-selective inhibitor of this PPI which is active in cells.^14,15^ We have also used SICLOPPS screens to identify compounds that inhibit both the HIF-1α/HIF-1β and the HIF-2α/HIF-1β PPI; the lead dual inhibitor compound (*cyclo-*CRLIIF) was shown to inhibit hypoxia-response in a variety of cell lines.^16^ Both sets of molecules were identified using the manual screening approach outlined above, and in both cases, the hit rate was low; one validated hit from a library of 3.2 million in the HIF-1 screen and three validated hits from the same library in the dual inhibitor screen. We therefore envisaged an alternative approach for SICLOPPS screening (Figure 1a), whereby the subjective step of manual colony selection is replaced by the collection of all surviving colonies, followed by NGS and retransformation of the SICLOPPS plasmids into the RTHS for another round of selection (1 round of biopanning). Due to the life/death readout of the screen, colonies containing the most potent inhibitors of the targeted PPI will grow faster. We therefore proposed that hit enrichment would be observed after several rounds of biopanning. We further hypothesized that by removing the potential bias from the hit-selection step, the revised approach would have a higher hit-rate, identify a more diverse range of active scaffolds, and potentially lead to the discovery of more potent hits.

## Results

### A Workflow for NGS Analysis of SICLOPPS Cyclic Peptide Libraries

To directly compare the performance of the new screening approach with the previous manual method, we used a CXXXXX library (CX_5_, where X is any of the 20 canonical amino acids) for screening. The randomized positions in the cyclic peptide were encoded by NNS codons (N is any of the four bases and S is either G or C), which allowed for all 20 proteinogenic amino acids to be present while eliminating the ocher (UAA) and opal (UGA) stop codons. To sequence this library by NGS, we employed a modular 2-step PCR protocol for introducing the flow cell adapters, indexes, and sequencing primer binding sites (Figure 1b). For the first PCR, the extracted plasmid library was used as a template, with custom PCR primers used to introduce the sequencing primer binding sites (i5 and i7 adapters). In the second PCR, the indexes (i5 and i7) and flow cell binding sequences (P5 and P7) were added (Figure 1b). The low variation in sequence between the SICLOPPS library samples arising from the fixed intein sequence flanking the 15 randomized bases encoding the SICLOPPS library was expected to be detrimental to sequencing quality due to the lack of diversity. Therefore, we also introduced an insert containing six randomized bases in between the i5 adapter and the C-intein to increase the DNA base usage diversity between samples and so improve cluster identification and sequence quality.^1^

We next developed a set of Python-based scripts for the analysis of the SICLOPPS library NGS. The first script is for quality control, which filters for the correct intein sequence and crops to the cyclic peptide coding region, before translating the peptide sequence encoded by the extein. A second script is used to monitor sequence enrichment over the rounds of biopanning and to identify similarities between the enriched sequences. The CX_5_ library was spiked with a SICLOPPS plasmid encoding *cyclo-*AAAAA (8% of total plasmids) to serve as an internal standard for monitoring sequence enrichment. Sequencing of the CX_5_ library showed good diversity, with a low prevalence of individual sequences in the initial library; more than 67% of analyzed sequences appeared between 1 and 10 times per million (1 M) reads (Figure 1c). Sequences with a low copy number of <100 made up 90% of all sequences in the naïve library, with the spiked CA_5_ control sequence being the only highly enriched sequence, observed at 10^4^–10^5^ copies per 1 M reads and comprising 8% of the library. Thus, with the NGS-analyzed naïve library and appropriate tools for sequence analysis in hand, we turned to screening this library in the HIF-1 RTHS.

### Screening and Biopanning

**Figure 2:**
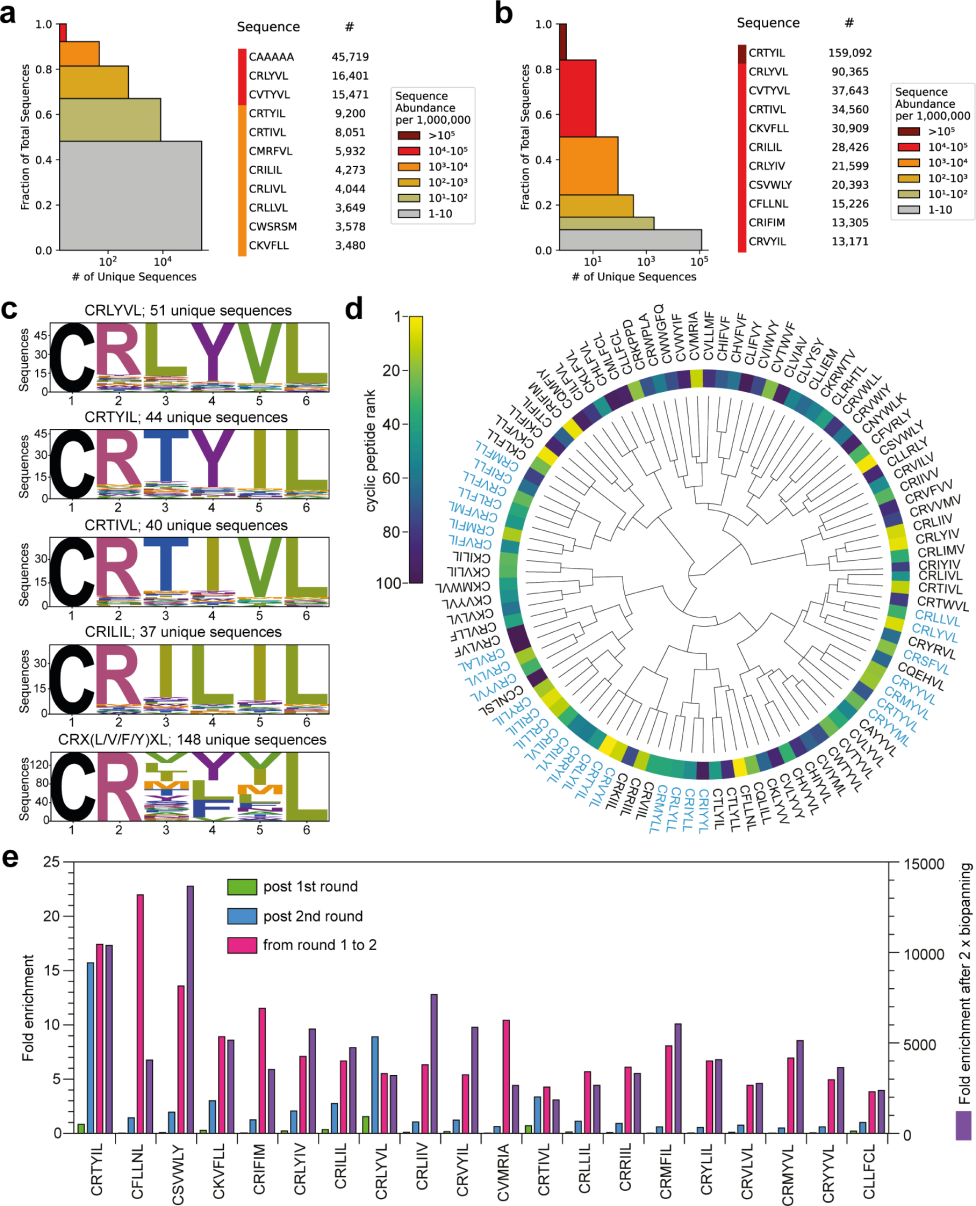
Identification and analysis of hit cyclic peptide sequences. (a) The abundance of sequences after round 1 of biopanning as visualized by a sequence redundancy plot; each colored box represents all sequences with the respective abundance of unique sequences per 1 M, and the number of unique sequences with the same abundance is on the *x*-axis. (b) The abundance of sequences after round 2 of biopanning as visualized by a sequence redundancy plot; each colored box represents all sequences with the respective abundance of unique sequences per 1 M, and the number of unique sequences with the same abundance is on the *x*-axis. (c) Sequence logos of the most enriched motifs in the library after two rounds of biopanning. (d) Hierarchical clustering diagram showing the relationship between the top 100 hit cyclic peptides after 2 rounds of biopanning. The blue sequences are those from the most abundant CRX(L/V/F/Y)XL motif. (e) Fold enrichment of the top 20 hits post round 1 of biopanning (green, left axis), post round 2 of biopanning (blue, left axis), from round 1 to round 2 of biopanning (pink, left axis), and from the initial library to round 2 of biopanning (purple, right axis).

The HIF-1 RTHS links the PPI between HIF-1α-P22 and HIF-1β-434 fusion proteins to the life/death of the host *E. coli* via three reporter genes.^14^ Interaction of HIF-1α and HIF-1β results in the formation of a chimeric P22/434 repressor that inhibits the transcription of the KanR and His3 reporter genes, which are required for cell survival in selection media (Figure S1). Disruption of the HIF-1α/HIF-1β PPI also disrupts the repressor complex, leading to transcription of the reporter genes and survival of colonies that contain cyclic peptide HIF-1 inhibitors. Thus, cells containing cyclic peptide inhibitors of the targeted PPI will survive and grow on selective media, whereas all others will not. The HIF-1 RTHS cells were transformed with the sequenced CX_5_ library and, after recovery, plated onto a selective medium. After incubation for 48 h at 37 °C, selection plates were scraped to collect all colonies, and the SICLOPPS plasmids were isolated. The extein coding region of these plasmids was subsequently sequenced by NGS. We observed sequence enrichment after one round of biopanning, with the three sequences (CAAAAA, CRLYVL, and CVTYVL) making up 7.8% of the entire library with over 10 000 copies each per 1 M reads (Figure 2a). The prevalence of the control sequence was reduced by 44% from 80 000 to 45 000 copies per million reads, which further indicated enrichment of cyclic peptide inhibitors of the HIF-1α/HIF-1β PPI in the RTHS. The next 47 sequences were present with over 1000 copies after round 1 which made up a further 15% of the library (Figure 2a). HIF-1 RTHS cells were transformed with the SICLOPPS plasmid library isolated from round 1 of biopanning, for a second round of selection. After incubation, all colonies were scraped, and plasmids were isolated and sequenced by NGS as before. We observed further sequence enrichment after the second round of biopanning, evident by the shift of the library population from the mostly low-prevalence sequences in both the naïve library and after one round of biopanning to mostly highly prevalent cyclic peptide sequences after round 2 of biopanning (Figure 2b). With 159 000 copies per 1 M reads, the most prevalent sequence is CRTYIL, while the next 14 sequences were all present with more than 10 000 copies per 1 M. The top 15 sequences make up over 50% of the entire library. The CA_5_ internal standard dropped to 0.5%, providing further evidence of enrichment of active inhibitors during biopanning.

While the representation of individual cyclic peptides was less than 0.005% in the initial library, this rose to 1.15% for *cyclo*-CRLYVL and 0.05% for *cyclo-*CVTYVL after one round of biopanning, ranked first and second respectively by % abundance. After the second round of biopanning; however, both sequences were overtaken by *cyclo-*CRTYIL, which was most prevalent. Other sequences (e.g., *cyclo*-CRILIL, *cyclo*-CFLLNL, and *cyclo*-CRIFIM) rose in the ranking between rounds 1 and 2 of biopanning (Figure 2b). This may indicate that they are better inhibitors of the targeted PPI than other enriched sequences, or that they confer a growth advantage through lower toxicity to the *E. coli* RTHS host (while still inhibiting the targeted PPI, which is required for the survival of the RTHS on selective media). Ranking the cyclic peptide sequences by % abundance after two rounds of biopanning, we observed good sequence homology among the top 25 hits (Table S1). Phylogenetic tree analysis using the blosum62 matrix^17^ of the top 25 sequences revealed close relationships between these sequences (Figure S2a). The short nature of the cyclic peptides made it challenging to obtain distinct clusters or potentially active motifs within the top 100 sequences using existing tools. The IEDB cluster tool (Figure S2b) showed most sequences to either be multiple clusters overlaid, or just one cluster with a few singletons, for similarity scores of 80% when using integrated cliques, while XSTREME (part of the MEME suite)^18^ identified one motif (CRXXL/VL) that can also be observed when manually assessing the data. While these tools may be useful for the sequence analysis of libraries of larger cyclic peptide ring sizes, such as those used for phage display or mRNA display (typically 12–20 amino acids), we found them not appropriate for the analysis of shorter (6 amino acids) SICLOPPS cyclic peptides.

We therefore performed a customized search of sequences sharing 80% homology (4 out of 5 amino acid overlap) within all enriched sequences (1528 unique) by using in-house Python scripts. Here, we classified all sequences with a copy number of at least 10 and a higher abundance in pan 2 than that in pans 1 and 0 as enriched. Using this approach, we observed high homology within the enriched sequences; analysis of *cyclo-*CRTYIL showed 44 unique sequences with 80% identity (Figure 2c), including *cyclo-*CRVYIL (which is also present in the top 25 and has a high similarity with *cyclo-*CRLYVL). The cyclic peptide with the highest number of related sequences (51) was *cyclo-*CRLYVL (Figure 2c), while the CXXYVL motif was observed in 65 of the enriched sequences. Strong similarities were found for *cyclo-*CRILIL with 37 highly similar sequences including *cyclo-*CRLLIL, *cyclo-*CRYLIL, and *cyclo-*CRILVL (Figure 2c). Arginine at position 1 and the two leucine residues at positions 4 and 6 are conserved in these sequences; we therefore queried the data for CRX(L/V)XL and found 51 unique sequences that mostly had a hydrophobic amino acid at position 5 (Figure S3). Extending this search to include phenylalanine and tyrosine at position 4 (i.e., CRX (L/V/F/Y)XL) resulted in 148 unique sequences (Figure 2c). These recurring motifs indicate the amino acids required in the cyclic peptides for disruption of the HIF-1α/HIF-1β PPI.

We sought to determine an approach to hit ranking that accounted for the final sequence representation as well as fold-enrichment between rounds of biopanning to identify which cyclic peptides to synthesize for further testing. For this, we calculated an adjusted inverse enrichment (enr_i-adj_) by combining the rank after the second round of biopanning (r_2_) with adjusted enrichment for both the initial library and the first round of biopanning to the second round of biopanning (enr_02_ and enr_12_): *enr*_i-adj_ = *rank*_2_ + 0.0001·*enr*_02_ + 0.01·*enr*_12_ We then reordered the sequences lowest to highest to obtain an overall rank.

The relationship between the newly ranked top 100 sequences was probed using hierarchical clustering on one-hot encoded data of the cyclic peptide sequences using the Radialtree Python module to generate a circular clustering diagram (Figure 2d). The top 100 sequences show three main branch points, which further branch into six secondary branches. The homology of the clustered sequences is readily visible, and the 20 most enriched sequences are spread evenly across the diagram, indicating the presence of multiple enriched motifs in the lead inhibitors. We next compared the fold enrichment of each cyclic peptide in the reranked top 20 hits after and between rounds of biopanning (Figure 2e). All the top 20 sequences showed good enrichment throughout the various points of the screen.

### Characterization of Hits In Vitro

**Figure 3:**
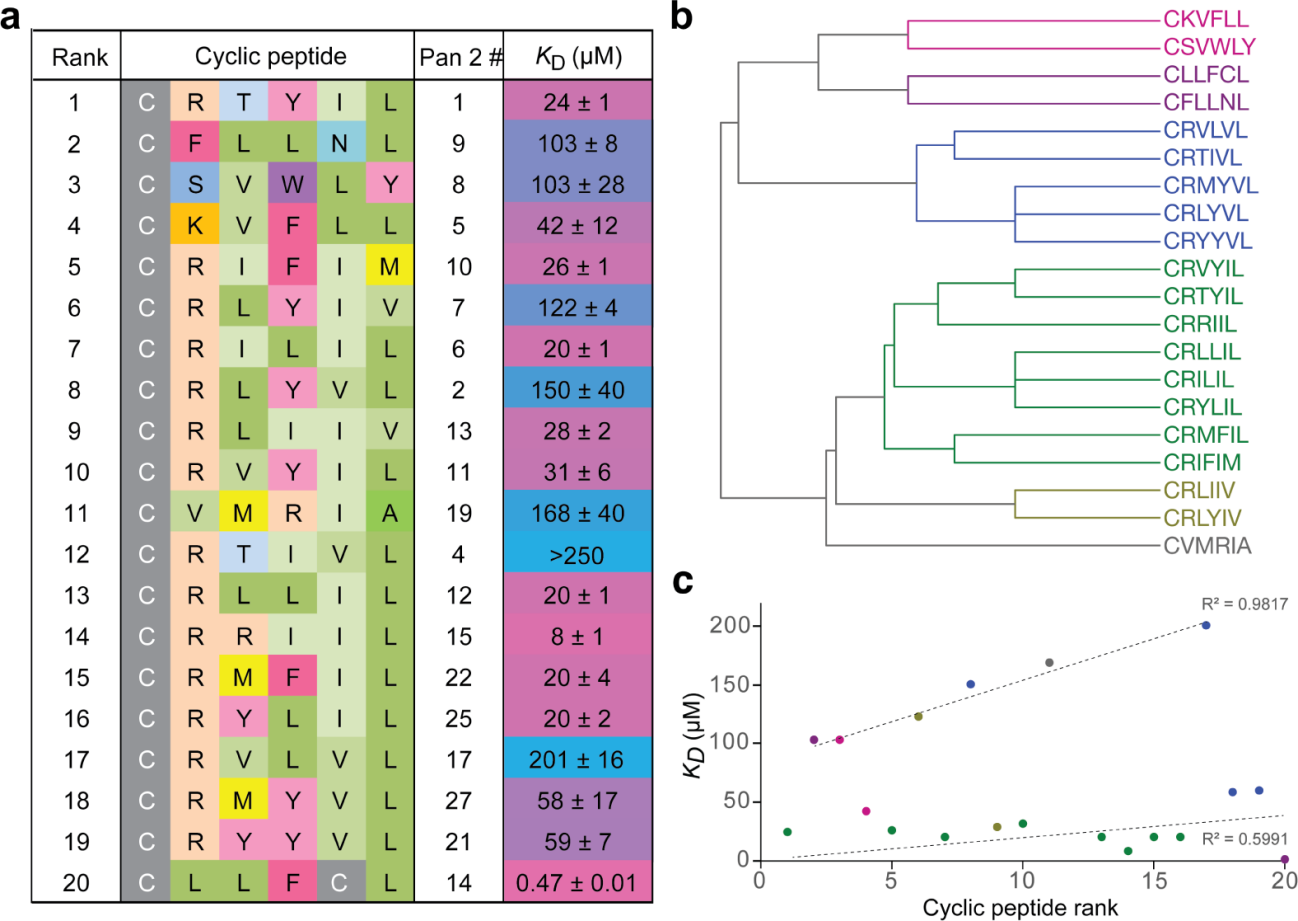
Characterization of cyclic peptide activity in vitro. a) Sequence similarity of the top 20 cyclic peptides and binding affinity for HIF-1α PAS-B by MST; for individual binding curves, see Figure S4; all data are mean (*n* = 3) ± SEM. b) Hierarchical clustering of the top 20 ranked peptides reveals 6 motif families. c) The correlation between rank and HIF-1α binding reveals two distinct clusters of cyclic peptides; the color of each cyclic peptide data point corresponds to that in b).

We synthesized the top 20 cyclic peptides from the ranking approach. We also synthesized *cyclo*-CLLFVY as a control.^14^ These compounds were assessed for binding to the PAS-B domain of HIF-1α by microscale thermophoresis (MST). We observed that 19 of the 20 selected cyclic peptides bound to HIF-1α (Figure 3a), a significantly better positive hit rate than previously reported for SICLOPPS screens.^14,16^ Furthermore, seven of these cyclic peptides bound HIF-1α with higher affinity than *cyclo-*CLLFVY (Figure S4). Of these seven peptides, six contained a CRXXIL motif with the fourth position favoring hydrophobic amino acids (aromatic and aliphatic), while the third position contained greater diversity in side-chain properties. These six cyclic peptides are part of an 8-membered cluster obtained via hierarchical clustering of just the top 20 via one-hot encoding (green, Figure 3b), where the remaining two sequences also show binding affinities under 30 μM (CRVYIL and CRIFIM). A second cluster with the very similar CRX (L/Y/I)VL consensus motif (blue, Figure 3b) showed less activity (>50 μM). The main difference between these two clusters is a change in the aliphatic amino acid, from isoleucine to valine at position 5. The clustering approach used here successfully identified a consistent effect from this relatively minor change, whereas the previous approach for SICLOPPS screening is unlikely to have identified this. The seventh peptide, *cyclo-*CLLFCL, deviated from this motif yet bound HIF-1α with the highest affinity, with a *K*_D_ of 470 ± 10 nM, which is >80-fold better than the control peptide *cyclo-*CLLFVY in the same assay (Figure S4). This difference in activity is surprising considering their sequence similarity, with only the last two amino acids being different. The IFC motif in the recently reported dual HIF-1/HIF-2 inhibitors was shown to be critical for its activity,^16^ it is therefore possible that the presence of two cysteines in *cyclo-*CLLFCL results in a bivalent inhibitor. Alternatively, the presence of two cysteines in this cyclic peptide raised the possibility of an intramolecular disulfide bridge constraining the conformation of the peptide, resulting in the higher affinity observed; the formation of a disulfide bond is unlikely due to the presence of the excess reducing agent in our assay buffer. Nonetheless, we made multiple attempts to synthesize the disulfide-bridged peptide via several different strategies, including exposing the parent monocyclic peptide to a variety of oxidation conditions used for disulfide bond formation, as well as preforming the disulfide bond on the linear peptide and then attempting to join the *N*- and C-termini via standard approaches. In all cases, this product was not observed, and the parent monocyclic peptide or intermolecular dimer product was recovered. We therefore concluded that formation of the disulfide bond was conformationally unfavorable for this peptide. It should be noted that the *K*_D_ value obtained by MST for *cyclo*-CLLFVY is significantly higher (i.e., indicating weaker binding) than that previously reported for a related molecule (Tat-*cyclo-*CLLFVY) by isothermal calorimetry (ITC).^14^ Previous control experiments indicated that Tat alone does not bind to HIF-1α; however, and an indirect contribution to binding from the Tat-tag (perhaps through increased solubility in the ITC assay buffer) cannot be ruled out. In addition, MST requires labeling of the protein, whereas ITC is a label-free method, and it is possible that the conjugated dye is partially blocking the binding site of the control cyclic peptide. It is also possible that both of these factors contribute to the observed difference from previously reported data.

We assessed the correlation between the different possible approaches to ranking our hits and their binding affinity to HIF-1α by MST. We observed relatively poor correlation between *K*_D_ and rank when the hits were ranked by either % present after two rounds of biopanning; the enrichment between rounds 0 and 2, or the enrichment between rounds 1 and 2 (Figure S5). Similarly, we saw only marginally improved overall correlation between binding affinity and our revised ranking approach that incorporates all three of the above factors (Figure 3c). However, this correlation data separate into two clusters, each of which shows a good correlation between rank and binding affinity (dotted lines, Figure 3c). Interestingly, the majority of the cyclic peptides in the high-affinity lower cluster were from the same branch of the hierarchical tree (dark and light green cluster in Figure 3b); similarly, the cyclic peptides in the lower-affinity upper cluster are from the same upper branch of the hierarchical tree (blue and pink in Figure 3b).

### Characterization of Hits in Cells

**Figure 4:**
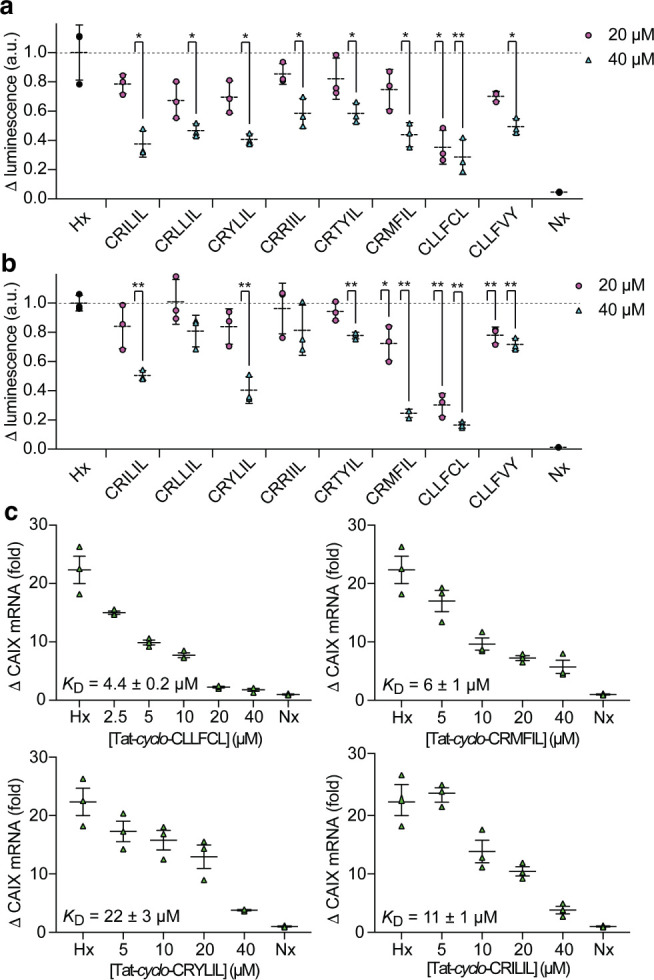
Assessing the cellular activity of cyclic peptide hits. a) U2OS-HRE-Luc cells were dosed with the indicated concentration of cyclic peptides and incubated in hypoxia for 6 h prior to the measurement of the luciferase signal. b) U2OS-HRE-Luc cells were dosed with the indicated concentration of cyclic peptides and incubated in hypoxia for 16 h prior to the measurement of the luciferase signal. c) The effect of selected cyclic peptides on the HIF-1-mediated expression of the CAIX gene in MCF-7 cells after 16 h in hypoxia by qPCR. Luciferase data is normalized to the signal in hypoxia (Hx = hypoxia = 1), qPCR data is shown as fold change from normoxic levels (Nx = normoxia = 1); all data is mean (*n* = 3) ± SEM; **p < 0.01, *p < 0.05.

We next assessed whether the seven most active cyclic peptides inhibited HIF-1 activity in cells. We used a HIF-1-dependent luciferase reporter assay constructed in human osteosarcoma U2OS cells (U2OS-HRE-Luc), where the formation of HIF-1 increases the expression of luciferase under hypoxic conditions.^19^ The initial testing showed no activity from these peptides, which was attributed to their potential lack of cell permeability. As with *cyclo*-CLLFVY, these cyclic peptides were Tat-tagged to enable their translocation through the cell membrane.^14^ A modified Tat sequence containing cysteine at its N-terminus was attached to the set cysteine of the cyclic peptides via a disulfide bond using a previously reported method.^14^ The seven Tat-tagged cyclic peptides and Tat-*cyclo-*CLLFVY (positive control) were incubated with U2OS-HRE-Luc cells in hypoxia for 6 h at 20 and 40 μM. All compounds caused a reduction in the luminescence signal at 40 μM (Figure 4a); Tat alone (40 μM) was used as a negative control and did not affect the luminescence signal in this assay (Figure S6a). The most active peptide in our in vitro assays, *cyclo-*CLLFCL, was also the most active compound in the cell-based reporter assay, reducing the luciferase signal by 65% and 71% at 20 and 40 μM respectively (Figure 4a). The remaining peptides demonstrated activity similar to that of Tat-*cyclo-*CLLFVY.

We next investigated the activity of the Tat-tagged cyclic peptides in cells incubated under hypoxia for 16 h in the same assay. Interestingly, the activity of the control Tat-*cyclo-*CLLFVY diminished after prolonged incubation with hypoxic cells, causing a 28% reduction in luminescence at 40 μM (Figure 4b). Four out of seven cyclic peptides retained their HIF-1 inhibitory function at 40 μM. Tat-*cyclo-*CLLFCL caused the largest effect at this time point too, with 70% and 84% signal reduction at 20 μM and 40 μM respectively (Figure 4b). This peptide was tested at multiple doses, and a dose dependent reduction in the hypoxia-mediated luciferase signal was observed, with an IC_50_ of 13 ± 4 μM (Figure S6b). The effect of each peptide on cell viability was also measured using a commercial nonlytic fluorescence-based cell viability assay (CellTiter-Fluor);^20^ we observed no toxicity for the seven Tat-tagged cyclic peptides at the highest doses tested after 6 h in hypoxia (Figure S6c) and 16 h in hypoxia (Figure S6d).

Cyclic peptides that caused a >50% signal reduction in the U2OS-HRE-Luc assay after 16 h in hypoxia were further assessed for their effect on the expression of a hypoxia-response gene. MCF7 breast cancer cells were dosed with each cyclic peptide, and the transcription of the HIF-1-mediated Carbonic anhydrase IX (CAIX)^21^ gene was measured by qPCR. We observed inhibition of CAIX expression for all four peptides tested (Figure 4c), with IC_50_ ranging from 4.4 ± 0.2 μM for *cyclo-*CLLFCL to 22 ± 3 μM for *cyclo-*CRYLIL. Together, these data illustrate the HIF-inhibiting activity of the identified inhibitors in cells. The fact that all of the top seven peptides bound HIF-1α in vitro and showed some level of HIF-1 inhibition in cells provides evidence for our hypothesis that the new SICLOPPS screening workflow reported here results in a lower false-positive rate, as well as more active hits both in vitro and in cells.

## Discussion

We present a new workflow that combines NGS and biopanning for the identification of cyclic peptides from a genetically encoded SICLOPPS hexa-peptide library. Analysis of the hit sequences revealed several closely related motifs, 19 of the 20 cyclic peptides synthesized were shown to bind the target protein in vitro, and all seven cyclic peptides tested in cells were active.

While the most enriched cyclic peptide (*cyclo*-CRTYIL) was not the most active, we observed modest agreement between the rank of sequences and their affinity for HIF-1α. When assessing which of the chosen parameters had the strongest correlation, we found that the adjusted inverse enrichment from the initial library to round 2 of biopanning, and from round 1 to round 2 of biopanning, both showed relatively good correlation. Conversely, the % enrichment after two rounds of biopanning did not align well with the observed affinity in vitro. This was mainly driven by the high enrichment for the top sequence and could be partly overcome by using the rank after two rounds of biopanning as a measure. Nonetheless, the most active peptide in vitro and in cells (*cyclo-*CLLFCL) was ranked higher for enrichment when ordering hits by % enrichment after 2 rounds of biopanning than when using our reranking that considers multiple parameters. These trends will be further investigated through the additional data sets generated from future screens.

Our studies also revealed several peptides with a shared CRXXIL motif, not identified in our previous manual SICLOPPS screens against this target.^14,16^ Combining the experimental data for these peptides with molecular dynamics simulations of the peptide structures could provide insight into the identity of the pharmacophore and the effect of conformation on binding. This information may be used in the future for the rational design of more potent inhibitors. Given the significantly improved hit-rate, we anticipate that the new approach for SICLOPPS screening and data analysis will be adopted in future SICLOPPS screens, resulting in more robust and potent hits.

## Data Availability

The data supporting the findings of this study are available within the paper and its Supporting Information. The Python scripts written for this study are available on the Tavassoli Lab GitHub: https://github.com/TavassoliLab. The raw data underpinning this study are openly available from the University of Southampton data repository at 10.5258/SOTON/D3197.

## Supporting Information Available

HIF-1 RTHS (Figure S1); analysis of the homology between the top 25 sequences (Figure S2); logo diagram showing sequences related to *cyclo*-CRX(L/V)XL (Figure S3); dose-response curves for top 20 hits (Figure S4); correlation between rank in screen and binding affinity to HIF-1α PASB (Figure S5); assessing the effect of cyclic peptide hits on cell viability (Figure S6); top 25 hits as ranked by % enrichment (Table S1); material and methods; ompound data containing HPLC and MS data for all compounds (PDF)
