# Supplementary material for: Next Generation SICLOPPS Screening for the Identification of Inhibitors of the HIF-1α/HIF-1β Protein–Protein Interaction
Source: ACS Chem Biol. 2024 Sep 23;19(10):2232–9. doi: 10.1021/acschembio.4c00494 (PMC11494503; doi:10.1021/acschembio.4c00494)
Supplement: Supplementary file 1 — cb4c00494_si_001.pdf [file cb4c00494_si_001.pdf]

Supplementary Data

Next Generation SICLOPPS Screening for the Identification of inhibitors of the HIF-1α/HIF-1β protein-protein interaction

Alexander McDermott,[1,#] Leonie Windeln,[1,#] Jacob Valentine,[1] Leonardo Baldassarre,[2] Andrew Foster,[1] and Ali Tavassoli[1,*]

[1]School of Chemistry, University of Southampton, Southampton, SO17 1BJ, U.K.

[2]Curve Therapeutics, Delta House, Southampton Science Park, Southampton, SO16 7NS, U.K.

[#]authors contributed equally to this work

[*]e-mail: ali1@soton.ac.uk

Supplementary Figures

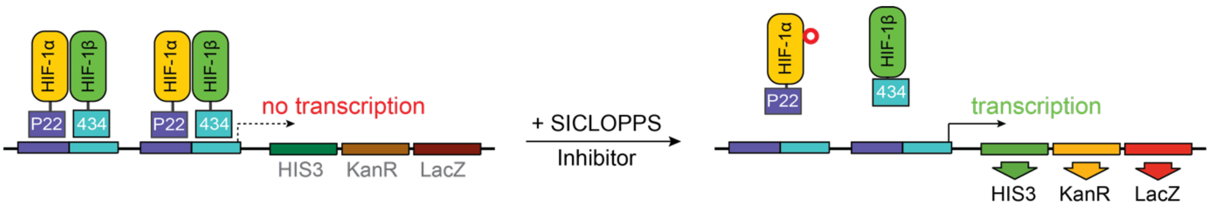

Figure S1. HIF-1 RTHS. The HIF-1 RTHS links the HIF-1α/HIF-1β PPI to the life or death of the *E. coli* host *via* three reporter genes. His3 is the yeast auxotroph of the *E. coli* HisB gene deleted in the RTHS strain, KanR confers kanamycin resistance, and LacZ encodes β-galactosidase. Thus, the HIF-1α/HIF-1β PPI results in cell death on selective media, while an inhibitor of this PPI will also disrupt the P22/434 repressor and allow transcription of the reporter genes and survival and growth of the host strain.

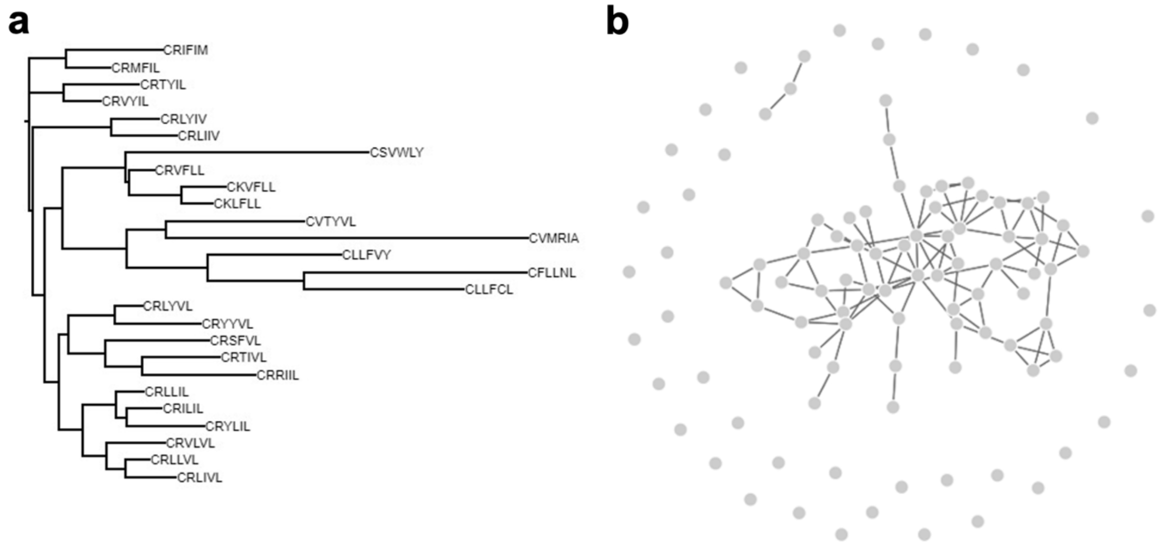

Figure S2. Analysis of the homology between the top 25 sequences. (a) A close sequence homology between the top 25 hits and CLLFVY is revealed using blosum62 matrix[1]. **(b)** IEDB cluster tool shows the majority of the top 25 sequences to be multiple clusters overlayed or just one cluster with a few singletons for similarity scores from to 10 to 80 % identity when using integrated cliques.

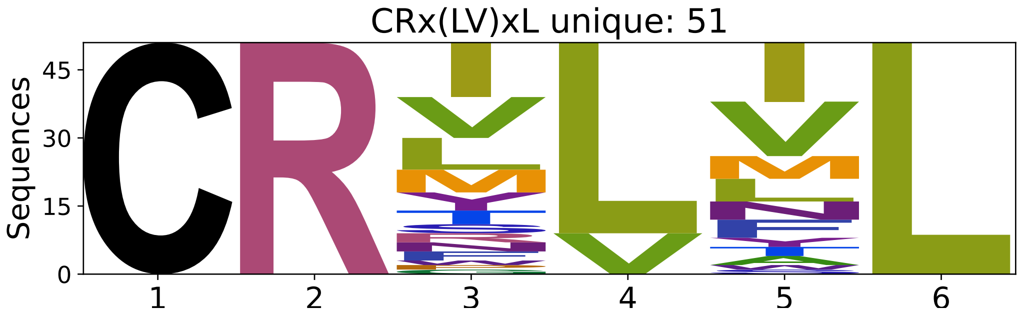

Figure S3. Logo diagram showing sequences related to *cyclo*-CRX(L/V)XL. Sequence logo of cyclic peptides with CRX(L/V)XL motif enriched in the library after 2 rounds of biopanning.

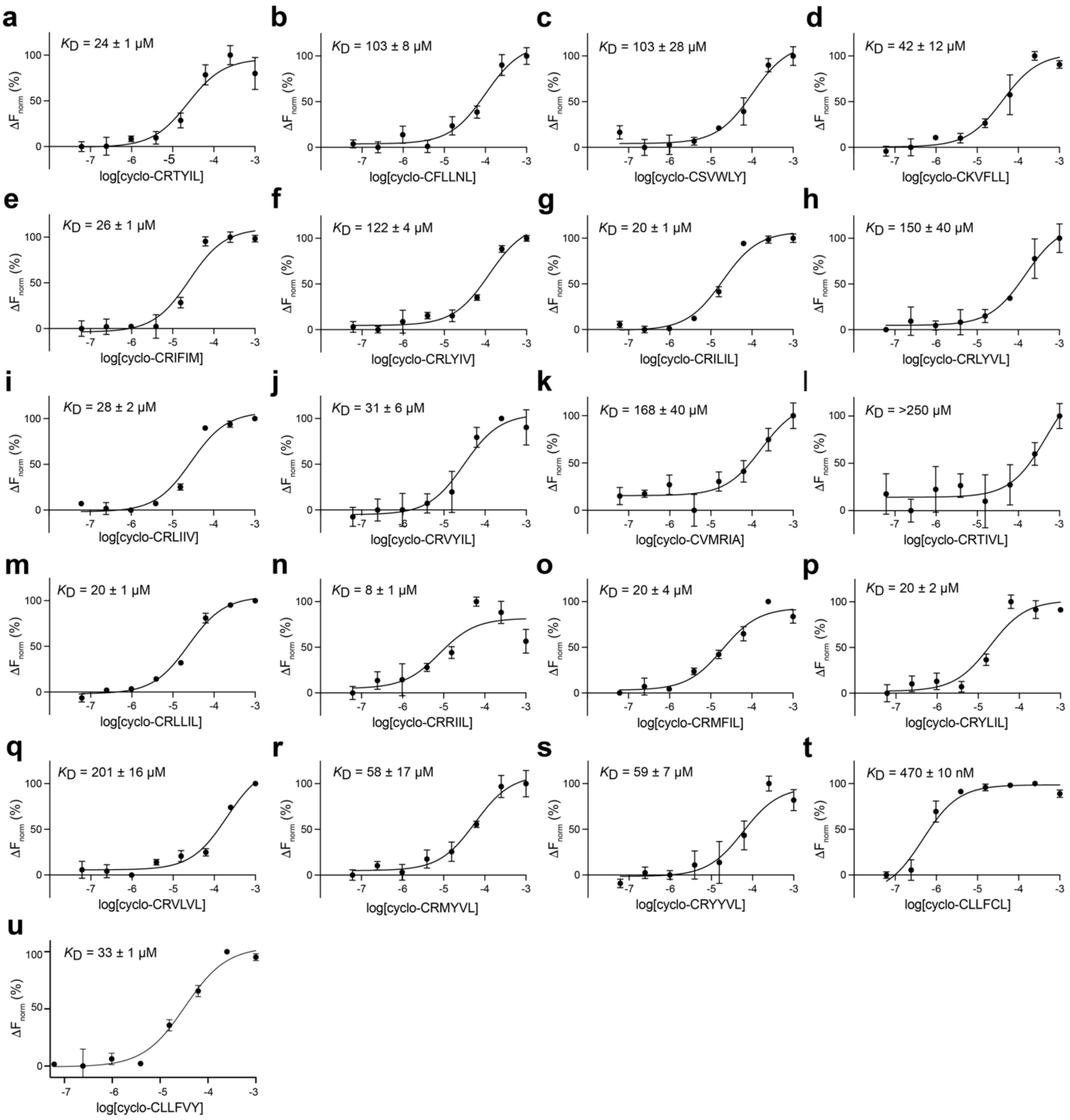

Figure S4. Dose-response curves for top 20 hits measured by MST against HIF-1α PAS-B. (a) *cyclo*-CRTYIL. **(b)** *cyclo*-CFLLNL. **(c)** *cyclo*-CSVWLY. **(d)** *cyclo*-CKVFLL. **(e)** *cyclo*-CRIFIM. **(f)** *cyclo*-CRLYIV. **(g)** *cyclo*-CRILIL. **(h)** *cyclo*-CRLYVL. **(i)** *cyclo*-CRLIIV. **(j)** *cyclo*-CRVYIL. **(k)** *cyclo*-CVMRIA. **(l)** *cyclo*-CRTIVL. **(m)** *cyclo*-CRLLIL. **(n)** *cyclo*-CRRIIL. **(o)** *cyclo*-CRMFIL. **(p)** *cyclo*-CRYLIL. **(q)** *cyclo*-CRVLVL. **(r)** *cyclo*-CRMYVL. **(s)** *cyclo*-CRYYVL. **(t)** *cyclo*-CLLFCL. **(u)** *cyclo*-CLLFVY All data shown as mean ± SEM, n=3.

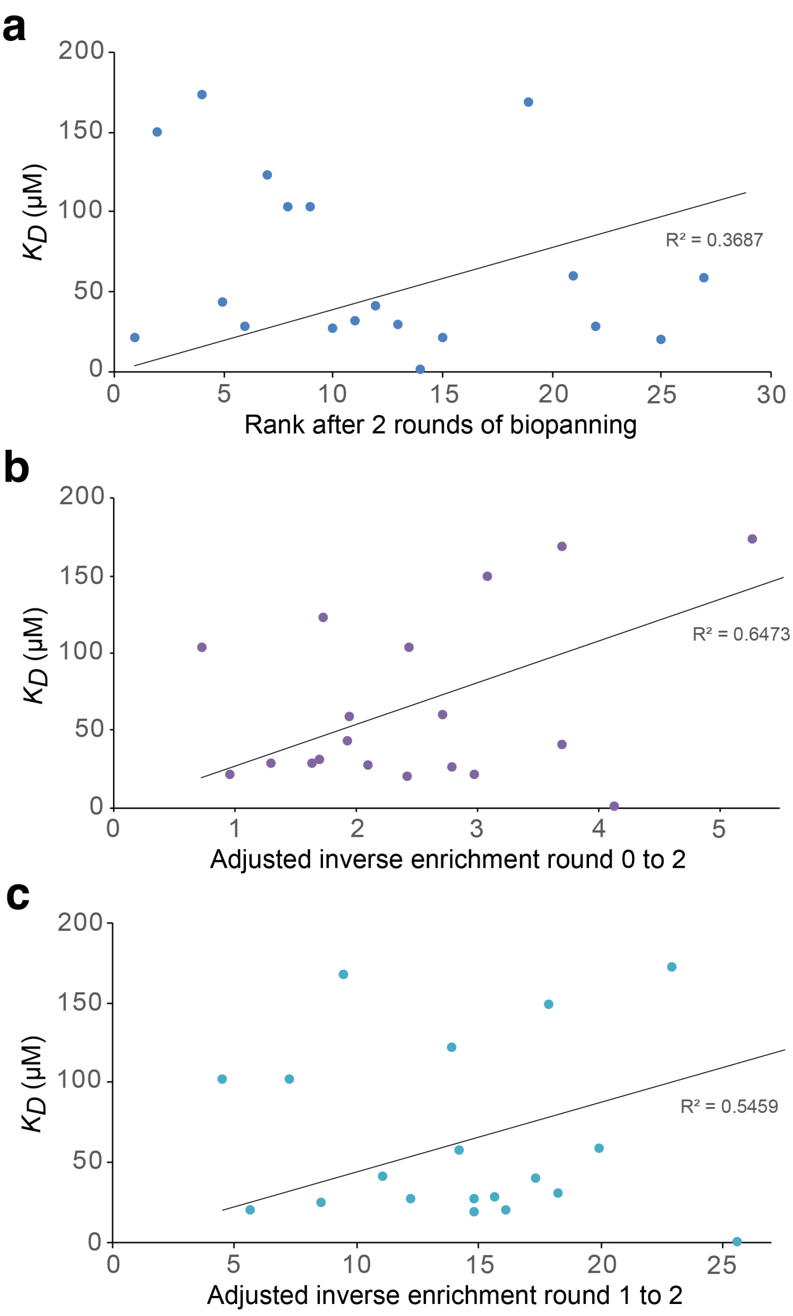

Figure S5. Correlation between rank in screen and binding affinity to HIF-1α PASB. (a) hits ranked by % present after 2 rounds of biopanning. **(b)** hits ranked by enrichment between round 0 to 2 of biopanning. **(c)** hits ranked by enrichment between round 1 to 2 of biopanning.

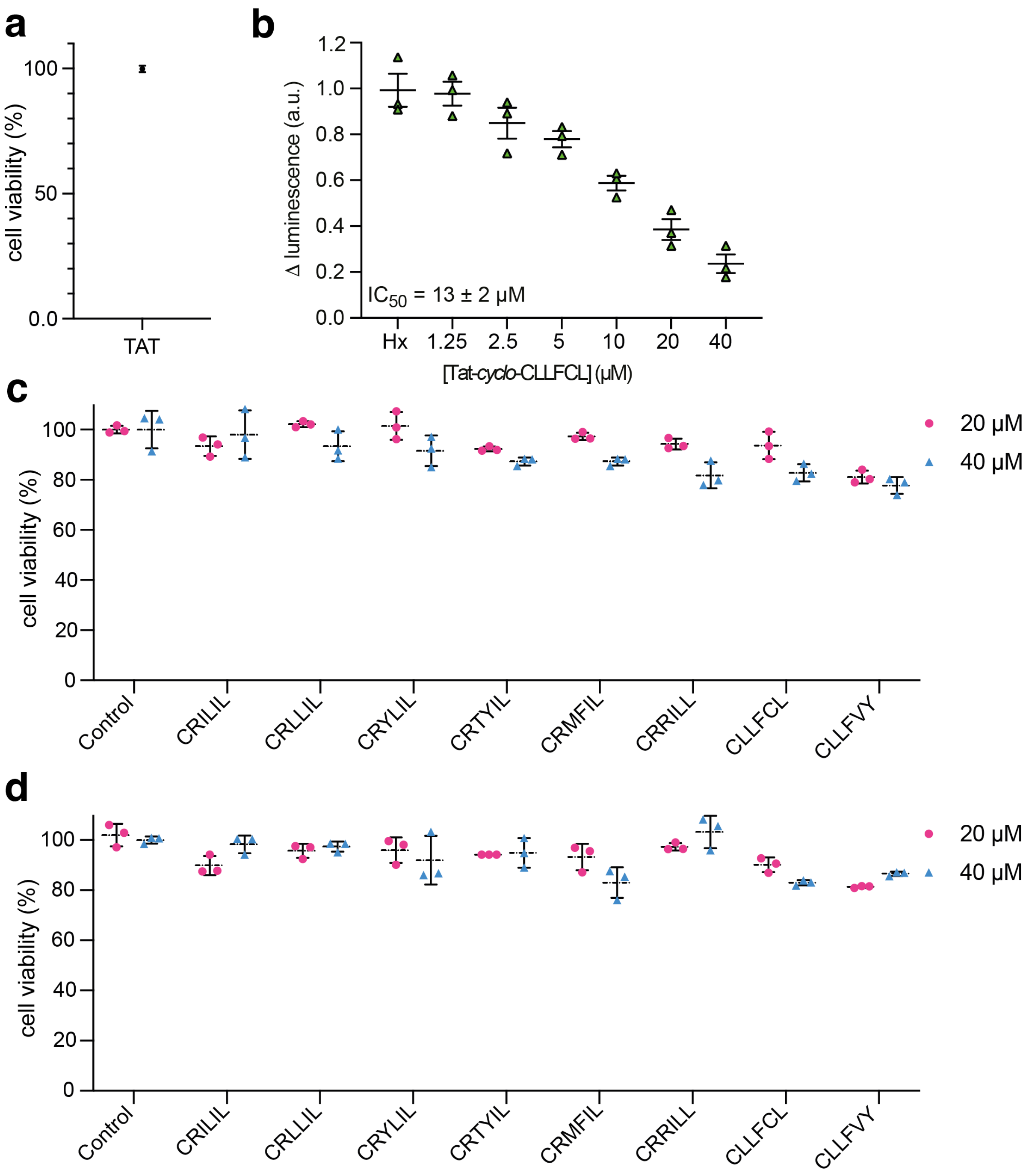

Figure S6. Assessing the effect of cyclic peptide hits on cell viability. a) The effect of Tat (40 µM) on the viability of U2OS-HRE cells. **b)** The effect of Tat-*cyclo*-CLLFCL on luciferase signal in U2OS-HRE-Luc cells. **c)** The effect of the cyclic peptide hits at 20 µM and 40 µM on the viability of U2OS-HRE-Luc cells incubated in hypoxia for 6 h **d)** The effect of the cyclic peptide hits at 20 µM and 40 µM on the viability of U2OS-HRE-Luc cells incubated in hypoxia for 16 h. All data is mean (n=3) ± SEM.

Table S1. Top 25 hits as ranked by % enrichment after 2 rounds of biopanning.

Sequence	Enrichment after pan 2 [%]
CRTYIL	15.7754602
CRLYVL	8.96160881
CVTYVL	3.73224084
CRTIVL	3.42675559
CKVFLL	3.06356655
CRILIL	2.81867953
CRLYIV	2.14181462
CSVWLY	2.02194222
CFLLNL	1.5092283
CRIFIM	1.31936618
CRVYIL	1.30557147
CRLLIL	1.19629144
CRLIIV	1.13846448
CLLFCL	1.07090731
CRRIIL	0.99121818
CRLLVL	0.87180766
CRVLVL	0.82719037
CRLIVL	0.74396019
CVMRIA	0.69684877
CKLFLL	0.68838103
CRYYVL	0.6783737
CRMFIL	0.6746171
CRVFLL	0.66245435
CRSFVL	0.61549689
CRYLIL	0.6083224

Material and Methods

All reagents were purchased from Fisher Scientific or Sigma Aldrich UK unless specified otherwise. Restriction enzymes and microbiology reagents were purchased from New England Biolabs Inc., FisherScientific (UK) or Promega (UK) and were used as directed by the manufacturer. Primers and oligonucleotides were synthesised by Integrated DNA Technologies (UK). DNA sequencing was carried out by Eurofins MWG Operon (Germany).

Construction of the RTHS and SICLOPPS library was conducted as previously described.[2]

All the cells used in this study were cultures in DMEM medium with 10% FBS and incubated in humidified atmosphere with 5% CO2 unless stated otherwise. For studies in hypoxia, cells were incubated in a Don Whitley DW35 hypoxia incubator at 1% Oxygen; isolation of mRNA and protein for these experiments was carried out in a hypoxic environment.

SICLOPPS screen

Electrocompetent RTHS-HIF 1 cells[2] were transformed with 100 ng freshly prepared SICLOPPS CX_5 library, prepared as previously detailed.[3] The recovery mixture was plated on 6 square plates containing M9 minimal media supplemented with 50 µg/mL ampicillin, 25 µg/mL Spectinomycin, 50 µg/mL kanamycin, 35 µg/mL chloramphenicol, 100 µM IPTG, 6.5 µM L-arabinose and 7.5 mM 3-amino triazole and incubate at 37 °C for 48 h. To ensure sufficie transformation efficiency, 10 µL transformation mixture was subjected to 10-fold serial dilutions plated on LB-agar with 35 µg/m Chloramphenicol. The efficiency was found to be ~6 $x10^7$, which covered the library. All cells were then harvested by resuspending the colonies in a total of 50 mL LB medium and transferring to a centrifuge tube and centrifugation for 30 min at 4000 rpm and 4°C. The cell pellet was then subjected to maxi prep (GeneJET Plasmid Maxiprep kit Thermo Scientific) and the resulting plasmid library was used for the next round of biopanning and NGS. The second round of bio panning was performed under the same conditions as the first. Here, 100 ng of pan 1 plasmid were used for the transformation of fresh RTHS cells.

NGS library preparation

For NGS, adapters were attached in two rounds of PCR. In the first PCR, 5 ng of plasmid library was used as a template and amplified in a 25 µL reaction using a high-fidelity master mix (SparQ HiFi PCR Master Mix, QuantaBio) and 0.5 µL of 100 µM forward primer (3'-TCGTCGGCAGCGTCAGATGTGTATAAGAGACAGNNNNNNGGTCTTCCCCAAGACCATAATTTTCTG) and 0.5 µL of 100 µM reverse primer (3'-GTCTCGTGGGCTCGGAGATGTGTATAAGAGACAGCAGCGTCAAGTAATGGAAAGGGAAG). Initial

denaturation was 2 min 98 °C followed by 22 cycles of 20 s denaturation at 98 °C, 30 s annealing at 62 °C and 30 s extension at 72 °C and final extension for 1 min. The PCR was size selectively purified using magnetic beads (SparQ PureMag Beads, QuantaBio) and eluted in 20 µL. For the second PCR (indexing PCR), different indexing pairs for each sample were used. The primers only differed in their indexing region. For i7 primers: 3'- CAAGCAGAAGACGGCATACGAGAT*CGATAG*GTCTCGTGGGCTCGG (*index*), for i5 primers: 3'- AATGATACGGCGACCACCGAGATCTACAC*ATGACT*TCGTCGGCAGCGTC (*index*).

The second PCR followed the same protocol as the first, and the PCR product was analysed for purity and quantity using High sensitivity DNA Screen Tape analysis with TapeStation 4150 (Agilent). For sequencing, all samples were diluted in ddH_2O to 4 nM.

Next Generation Sequencing

Barcoded samples were denatured and pooled according to the manufacturer's instructions and diluted to 15 pM. Depending on other samples to be run alongside, 10-30 % PhiX were added before sequencing was performed on an Illumina MiSeq using a v3 150 cycle kit.

All sequences with Q30 were taken forward for analysis using our custom python scripts. Briefly, we aligned all sequences to the known intein sequence before cropping and translating to cyclic peptide sequence using biopython.[4] We then calculated the abundance of each cyclic peptide and generated a sequence redundancy plot. The sequence logos were generated using logomaker.[5] Hierarchical clustering of sequences was performed using AgglomerativeClustering.[6] The distance matrix was calculated either by blosum62 similarity score (inverse) or one-hot encoded sequence data.

HIF-1α PAS-B domain expression, purification and MST

The HIF-1α PAS-B domain was expressed and purified as previously described; the protein was prepared for MST according to the manufacturer's instructions and MST assays conducted as previously described.[7]

HIF Luciferase Reporter Assays

Assays were conducted in previously reported U2OS-HRE-Luciferase (U2OS-HRE-Luc) cells.[2] Upon reaching 80-90% confluency, U2OS-HRE-Luc were plated (10 000 cells/well) in 96-well plates (Perkin-Elmer) and incubated in a cell incubator for 16 h. The cells were treated with cyclic peptide at stipulated concentrations and left to incubate for 2 h in normoxia before being transferred to the Hypoxia Workstation and incubated for 6 h or 16 h. After the allotted incubation time, plates were removed from the Hypoxia Workstation and left to equilibrate to room temperature for 30 minutes.

Luciferase activity was determined using the Bright-Glo™ assay kit (Promega) following the manufacturer's protocol. Luminescence was measured using a ClarioStar plate reader. The luciferase signal of cells treated with peptide were normalised with untreated cells incubated in hypoxia and normoxia.

Cell viability Assays

Cell viability assays were conducted concurrently with HIF Luciferase Reporter Assays whereby cells were incubated and treated with peptide under identical conditions. Following equilibration to room temperature, cell viability was assessed using the CellTiter-Fluor™ assay (Promega) according to the manufacturer's protocol. Fluorescence was measured using a ClarioStar plate reader. The fluorescence signal of cells treated with peptide were normalised with untreated cells incubated in hypoxia.

qPCR

MCF-7 cells were plated on 35 x 10 mm culture plates and grown in an incubator till 80-90% confluency. Fresh DMEM media was applied to cells before treatment with peptide at specified concentrations and incubated in a cell incubator for 2 h. Subsequently, cells were transferred to a hypoxic workstation for 16 h. Total RNA was isolated using the SV Total RNA Isolation System kit (Promega) following the manufacturer's protocol and quantified using a Nanodrop ND-1000 spectrophotometer. Complementary DNA was synthesised using the GoScript™ Reverse Transcriptase kit (Promega) following the manufacturer's protocol. Quantitative real-time PCRs (qPCR) was performed in triplicate using iTaq Universal SYBR Green Supermix and primers for CA9 (NM_001216.3), 18S (NCBI ID: 106631781) and HPRT1 (NM 012583). Gene expression values were normalised to 18S and HPRT1. Data was plotted and IC50s calculated using Prism software with a four-parameter logistic model ([Inhibitor] vs normalised response).

Primer sequences used for qPCR experiments

Gene Name	Sequence
Carbonic Anhydrase 9 (CA9)	Fw: 5'- CAACACGGGAAACCTCACCC -3'<br>Rv: 5'- CCGGGCCCTCCTCCAGAAAG -3'
18S	Fw: 5'- CAACACGGGAAACCTCACCC – 3'<br>Rv: 5' - CTCCACCAACTAAGAACGGCC – 3'
Hypoxanthine Phophoribosyltransferase 1 (HPRT1)	Fw: 5' - CTCATGGACTGATTATGGACAGGAC – 3'<br>Rv: 5' - GCAGGTCAGCAAAGAACTTATAGCC – 3'

Synthetic Chemistry

2-Chlorotrityl Chloride Resin Loading

Solid Phase Peptide Synthesis Peptides were synthesised by Fmoc solid-phase peptide synthesis using 2-Chlorotrityl chloride (2-CTC) resin which were loaded with the first amino acid in-house. The first amino acid was loaded onto the 2-CTC (1 eq) by swelling the resin in DCM in a sinter funnel followed by the addition of Fmoc amino acid (3 eq) dissolved in DCM and DIPEA (3 eq). The resin was agitated via argon flow for 2 hours. MeOH (5 mL) was then added to the funnel to cap any unreacted resin and agitated for an additional 15 minutes. The reaction mixture was then removed under reduced pressure and the resin washed with DMF, DCM and Et_2O. The resin was then air dried and 10 mg was accurately weighed into a glass vial and the Fmoc protection group was removed via 20% Piperidine in DMF. A 100 mL aliquot of the reaction mixture was diluted 1:10 in DMF and the UV absorbance of dibenzofulvene-piperidine adduct was measured at a wavelength of 301 nm using a NanoDrop ND-1000 Spectrophotometer (NanoDrop Technologies, USA). The absorbance value was used in the following equation to determine the loading of the resin in mmol/gram.

L = (A301 x V x d)/(Ec x w x M)

L= Resin loading (mmols/gram)

A301= Absorbance at 301 nm

V = Volume of the cleavage solution (1 mL)

d = dilution factor (10)

Ec = Extinction coefficient (= 7800mL/mmol.cm)

w = width of the NanoDrop (= 0.1 cm)

M = Weight of the resin sample (10 mg)

Solid Phase Peptide Synthesis

All peptide synthesis was performed at a 0.1 mmol scale. Coupling and deprotection steps were performed at room temperature in 6 mL syringes with a 0.2 mm Frit inside. Reaction mixtures within syringes were placed on a rocker which was slowly agitated from side to side. Coupling solutions were prepared using Fmoc-protected amino acid (3 eq.) and Oxyma Pure (3 eq.) dissolved in DMF, to which DIC (3 eq.) was added. The mixture was stirred for 3 minutes, after which the solution was added to the resin and agitated for 1 h. Upon reaction completion, the resin was washed three times with DMF, three times with DCM and finally three times with Et_2O. Successful coupling reactions were checked using the Kasier test prior to Fmoc deprotection. Fmoc deprotection was carried out by agitating the resin with 20% piperidine in DMF for 20 mins. The resin was washed as before, and

successful deprotections were checked using the Kaiser test prior to moving on to the next coupling. After deprotection of the final amino acid, the dry resin was transferred to a 12 mL vial and stirred with 20% HFIP in DCM (10 mL per mmol peptide) for 2 h to cleave the peptide from the resin. The mixture was filtered through a cotton filter, and the filtrate concentrated *in vacuo*. Peptides were precipitated from the remaining residue with cold Et_2O. The Et_2O was removed, and precipitate triturated a further two times with cold Et_2O, after which the solid was dried on a rotary evaporator. Depending on purity (determined by LCMS), the peptide was either used for subsequent reactions without further purification or was dissolved in a H_2O:MeCN mixture (1:1) prior to purification by reverse-phase chromatography.

<u>Peptide Cyclisation</u>

The linear peptide was dissolved in DMF (1 mL DMF per mg peptide) followed by the addition of DIPEA (3 eq) and HATU (1.5 eq) and the reaction was left to stir at room temperature overnight. After reaction completion, the mixture was concentrated on a rotary evaporator and the remaining residue purified by reverse-phase chromatography. The fractions were checked via LC MS and pooled together and concentrated *in vacuo*.

<u>TFA Deprotection</u>

Acid labile protecting groups on cyclic peptides were dissolved in a TFA cleavage cocktail TFA/TIPS/H_2O (95:2.5:2.5% v/v) and stirred at ambient temperature for 1 hour. Excess TFA was then removed under reduced pressure and purified by reverse phase HPLC chromatography.

<u>Synthesis of TAT – tag</u>

CGRKKRRQRRRPPQ linear peptide was synthesised using 2-CTC Solid Phase peptide synthesis. The peptide was cleaved from the resin utilising a cleavage cocktail containing TFA/TIPS/H_2O (95:2.5:2.5% v/v) for 2 h at RT. The resin was removed via filtration and the peptide was concentrated *in vacuo*. The peptide was triturated with cold Et_2O (-80 °C) which was removed followed by two additional washes of ice cold Et_2O (-80 °C). The resulting solid was further dried via rotary evaporation.

LR MS (ESI+): m/z (M+H+) calculated for $C_{73}H_{136}N_{36}O_{17}S$, 1822.2, found; 911.6 [M+2H]2+, 608.3 [M+3H]3+, 456.4 [M+4H]4+, 365.4 [M+5H]5+.

<u>Synthesis of 2-Mercaptopyridine (Spy) protected Cysteine-TAT</u>

CGRKKRRQRRRPPQ (834 mg, 0.46 mmol, 1 eq) was dissolved in DMF (100 mL) to which was added Aldrithiol-2 (1 g, 4.6 mmol, 10 eq) and was left to stir at ambient temperature overnight. The mixture was concentrated *in vacuo*, and the product C(Spy)GRKKRRQRRRPPQ was precipitated from the reaction mixture by drop-wise addition of the oily mixture into ice cold Et_2O (-80 °C). The Et_2O was removed, and the precipitate washed two additional times followed by drying via rotary evaporation. The crude was then purified via biotage chromatography. Fractions were analysed via LC/MS, desired fractions were then pooled, concentrated *in vacuo*, and lyophilised to yield C(Spy)GRKKRRQRRRPPQ as a white solid (400 mg, 0.21 mmol, 45%).
LR MS (ESI+): m/z (M+H+) calculated for $C_{78}H_{139}N_{37}O_{17}S_2$, 1930.1, found; 966.2$[M+2H]^{2+}$, 644.4 $[M+3H]^{3+}$, 483.7 $[M+4H]^{4+}$, 387.2 $[M+5H]^{5+}$.

Chromatography

Purification was performed on a Biotage-Isolera Spektra Four Purification system using a SFAR 30g D DUO Column for reverse phase chromatography. A binary solvent elution system of 0.1% TFA/H_2O (Solution A) and 0.1%TFA/MeCN (Solution B) was used to equilibrate the column at 90% Solution A and 10% Solution B for 3 column volumes at 25 mLmin^{-1}. Compounds were dissolved in either 2 mL of DMF or MeCN and injected directly onto the column. A series of gradients were set according to the results of the LC/MS of the crude material. Fractions were collected when UV 220 nm exceeded a 40 mAu threshold. Fractions were analysed via LC/MS, desired fractions were then pooled, concentrated *in vacuo,* and lyophilised to yield the desired product.

Preparative HPLCs

Samples were injected as solutions of either DMF or MeCN via a Waters Flex inject system into a RP-HPLC system consisting of a Waters 1525 binary pump coupled to a Waters 2998 Chapter 8 133 photodiode array detector. Cyclic peptides were then purified using a Waters Atlantis T3 column (5.0 µm particle size, 19 × 100 mm). A binary solvent system consisting of 0.1 % TFA / water, (Solution A) and 0.1 % TFA / MeCN (Solution B) were ran at gradients according to their retention time determined by analysis via LC/MS of the crude. Fractions with an absorbance of 220 nM absorbance were collected, analysed via LC/MS, desired fractions pooled, concentrated *in vacuo,* and lyophilised to yield the desired product.

Electrospray - LC/MS Analysis

Samples were analysed using a Waters (Manchester, UK) TQD mass spectrometer equipped with a triple quadrupole analyser. Samples were introduced to the mass spectrometer via an Acquity

HClass quaternary solvent manager (with TUV detector at 254nm, sample and column manager). Ultra-performance liquid chromatography was undertaken via a Waters BEH C18 column (50 mm x 2.1mm 1.7µm). Gradient 20% acetonitrile (0.2% formic acid) to 100% acetonitrile (0.2% formic acid) in five minutes at a flow rate of 0.6 mL/min. Low-resolution mass spectra were recorded using positive ion electrospray ionisation.

High Resolution Mass Spectrometry

Samples were analysed using a MaXis (Bruker Daltonics, Bremen, Germany) mass spectrometer equipped with a Time of Flight (TOF) analyser. Samples were introduced to the mass spectrometer via a Dionex Ultimate 3000 autosampler and uHPLC pump. Gradient 20% acetonitrile (0.2% formic acid) to 100% acetonitrile (0.2% formic acid) in five minutes at 0.6 mL/ min. Column, Acquity UPLC BEH C18 (Waters) 1.7-micron 50 x 2.1 mm. High resolution mass spectra were recorded using positive ion electrospray ionisation.

Analytical HPLC

Analytical HPLCs were carried out on an Agilent 1260 Infinity II HPLC system running a binary solvent system consisting of 0.1 % TFA / water, (Solution A) 0.1 % TFA / MeCN (Solution B). Cyclic peptide PROTACs were injected (50 mL) onto a Poroshell 120 EC-C18 column (2.7 µm particle size, 3.0 x 100mm) and the gradient shown below ran at a flow rate of 0.625 mL/ min. UV absorbance was recorded at both 220 and 280 nm. Peaks detected at 220 UV absorbance were integrated to determine compound purity.

Compound data

cyclo-CRTYIL

The linear peptide was synthesised using 2-Chlorotrityl chloride resin SPPS at 0.1 mmol scale. The linear peptide was then cyclised via HATU cyclisation conditions. The cyclic peptide crude was then subjected to TFA deprotection and was subsequently purified via reverse-phase HPLC chromatography to yield *cyclo*-CRTYIL as a white solid (12 mg, 0.016 mmol, 16%).

LR MS (ESI$^+$): m/z (M+H$^+$) calculated for $C_{34}H_{55}N_9O_8S$, 750.4, found; 750.7.

HRMS (ESI$^+$): m/z (M+H$^+$) calculated for $C_{34}H_{55}N_9O_8S$, 750.3967, found; 750.3966 (0.2ppm)

Analytical HPLC Rt: (220 nm) 10.118 min (98.9% Purity).

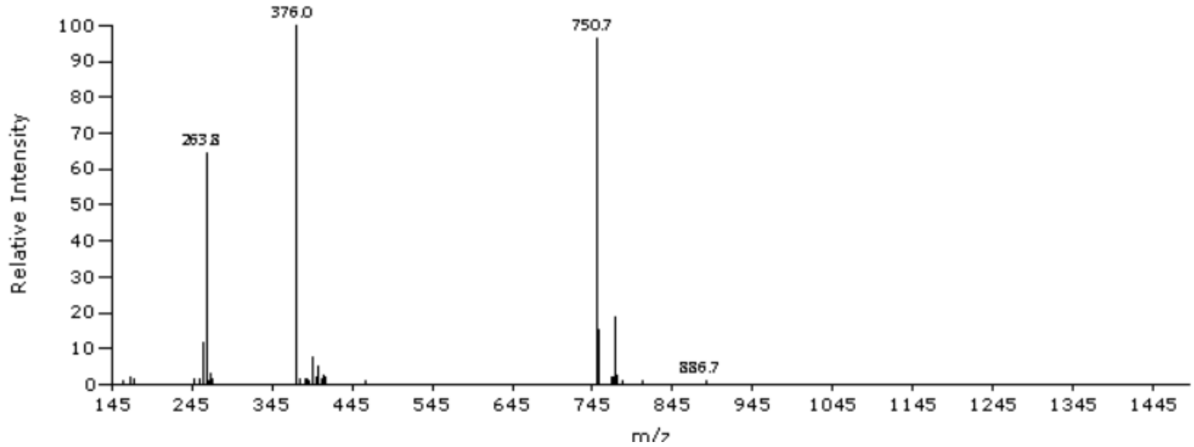

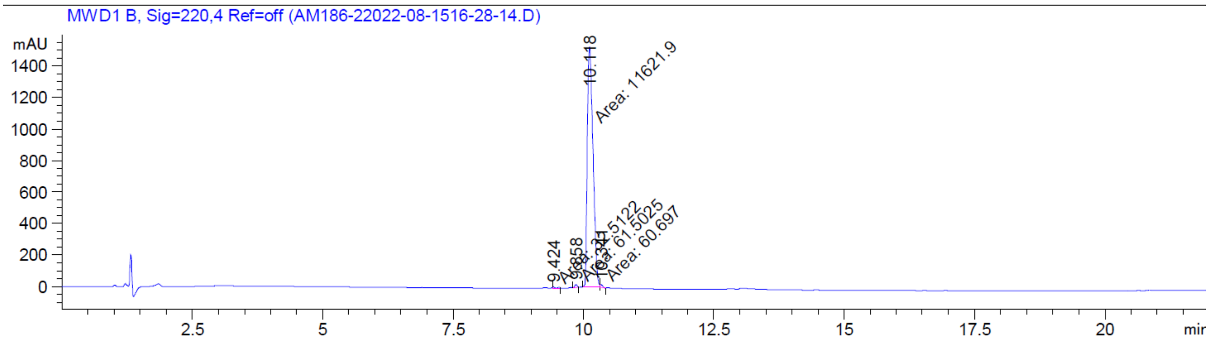

Peak #	RetTime [min]	Type	Width [min]	Area [mAU*s]	Height [mAU]	Area %
1	9.424	MM	0.0736	32.51222	7.36432	0.2761
2	9.858	MM	0.0539	61.50249	19.01447	0.5222
3	10.118	MM	0.1269	1.16219e4	1526.51135	98.6863
4	10.341	MM	0.0577	60.69704	17.53372	0.5154

cyclo-CFLLNL

The linear peptide was synthesised using 2-Chlorotrityl chloride resin SPPS at 0.1 mmol scale. The linear peptide was then cyclised via HATU cyclisation conditions. The cyclic peptide crude was then subjected to TFA deprotection and was subsequently purified via reverse-phase HPLC chromatography to yield *cyclo*- CFLLNL as a white solid (5 mg, 0.007mmol, 7%).

LR MS (ESI$^+$): m/z (M+H$^+$) calculated for $C_{34}H_{53}N_7O_7S$, 704.4, found; 704.5 $[M+H]^+$

HRMS (ESI$^+$): m/z (M+H$^+$) calculated for $C_{34}H_{53}N_7O_7S$, 704.3800, found; 704.3784 (2.2 ppm error).

Analytical HPLC Rt: (220 nm) 7.872 min (99.9% Purity)

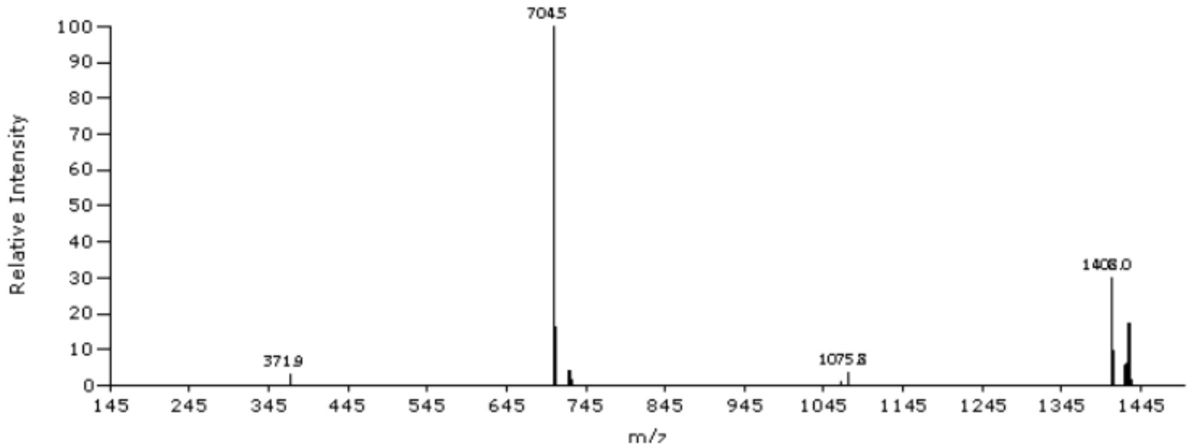

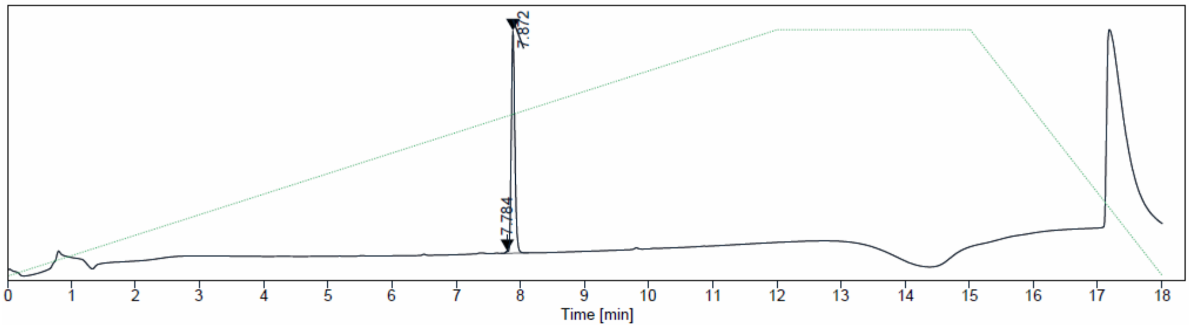

Signal: MWD1B,Sig=220,4 Ref=400,50

RT [min]	Type	Width [min]	Area	Height	Area%	Name
7.784	BV f	0.0533	2.9878	1.5367	0.1235	
7.872	BB	0.4380	2415.8222	631.4477	99.8765	
		Sum	2418.8100			

<u>*cyclo*-CSVWLY</u>

The linear peptide was synthesised using 2-Chlorotrityl chloride resin SPPS at 0.1 mmol scale. The linear peptide was then cyclised via HATU cyclisation conditions. The cyclic peptide crude was then subjected to TFA deprotection and was subsequently purified via reverse-phase HPLC chromatography to yield *cyclo*- CSVWLY as a white solid (10 mg, 0.013 mmol, 13%).

LR MS (ESI$^+$): m/z (M+H$^+$) calculated for $C_{37}H_{49}N_7O_8S$, 752.3, found; 752.6 [M+H]$^+$

HRMS (ESI$^+$): m/z (M+H$^+$) calculated for $C_{37}H_{49}N_7O_8S$, 752.3436, found; 752.3428 (1.1 ppm error).

Analytical HPLC Rt: (220 nm) 6.834 min (81% Purity)

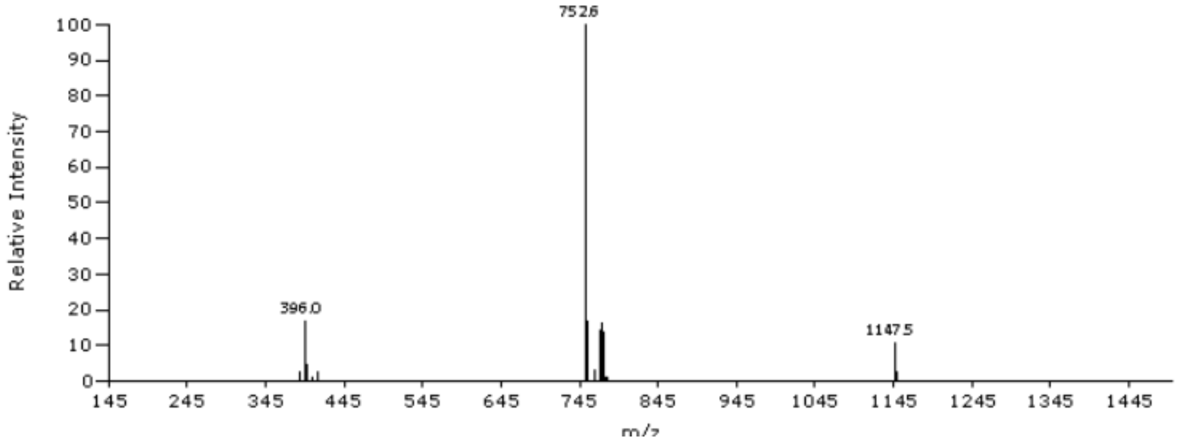

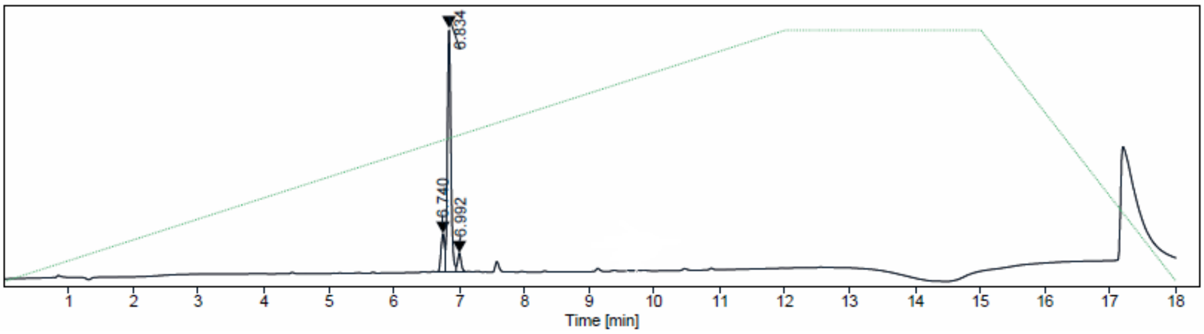

RT [min]	Type	Width [min]	Area	Height	Area%	Name
6.740	BV	0.1327	791.6041	209.6925	13.0141	
6.834	VV	0.1644	4896.2749	1333.6354	80.4958	
6.992	VB	0.1558	394.7697	102.7493	6.4901	
		Sum	6082.6487			

cyclo-CKVFLL

The linear peptide was synthesised using 2-Chlorotrityl chloride resin SPPS at 0.1 mmol scale. The linear peptide was then cyclised via HATU cyclisation conditions. The cyclic peptide crude was then subjected to TFA deprotection and was subsequently purified via reverse-phase HPLC chromatography to yield *cyclo*- CKVFLL as a white solid (21 mg, 0.030 mmol, 30%).

LR MS (ESI$^+$): m/z (M+H$^+$) calculated for $C_{35}H_{57}N_7O_6S$, 704.4, found; 704.9 $[M+H]^+$

HRMS (ESI$^+$): m/z (M+H$^+$) calculated for $C_{35}H_{57}N_7O_6S$, 704.4164, found; 704.4168 (-0.6 ppm error).

Analytical HPLC Rt: (220 nm) 5.944 min (94% Purity).

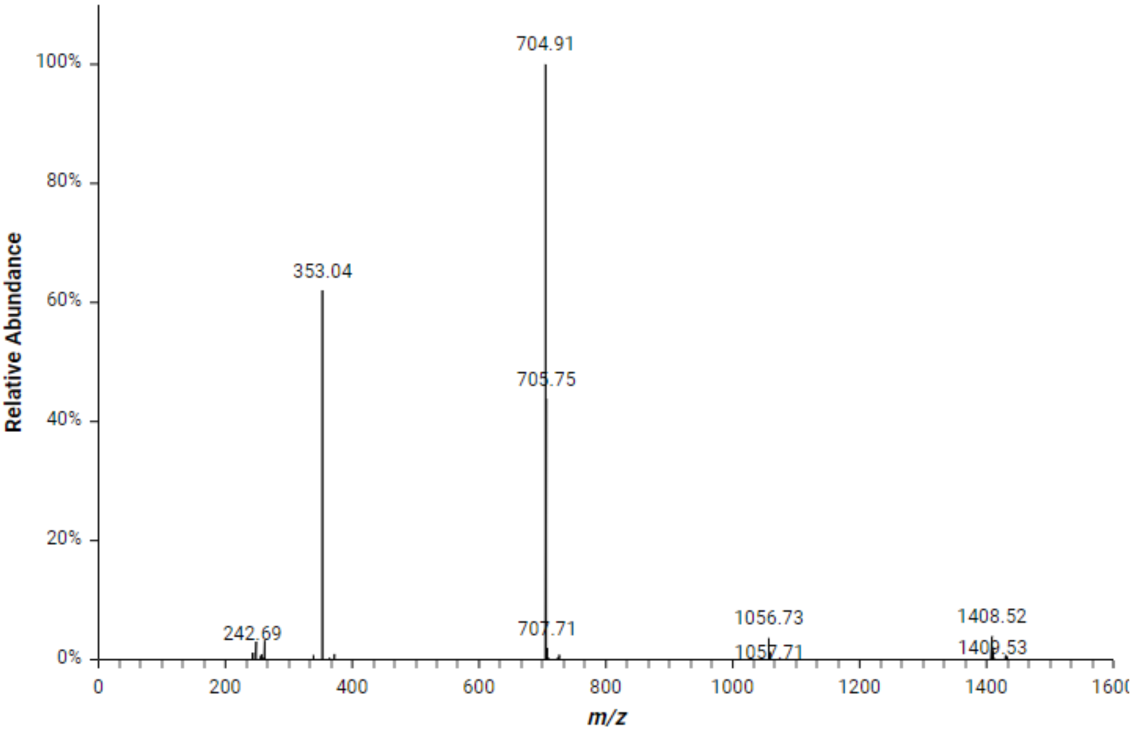

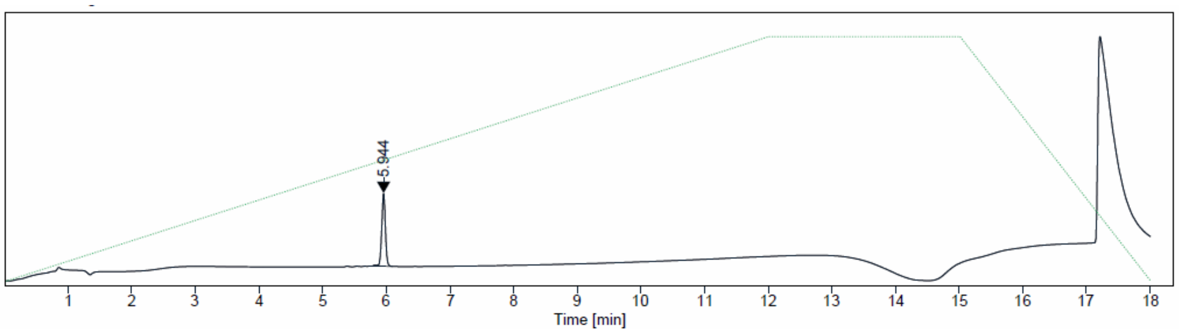

Signal: MWD1B,Sig=220,4 Ref=400,50

RT [min]	Type	Width [min]	Area	Height	Area%	Name
5.944	BB	0.3267	911.9883	228.6905	100.0000	
		Sum	911.9883			

cyclo-CRIFIM

The linear peptide was synthesised using 2-Chlorotrityl chloride resin SPPS at 0.1 mmol scale. The linear peptide was then cyclised via HATU cyclisation conditions. The cyclic peptide crude was then subjected to TFA deprotection and was subsequently purified via reverse-phase HPLC chromatography to yield *cyclo*-CRIFIM as a white solid (17 mg, 0.022 mmol, 22%).

LR MS (ESI$^+$): m/z (M+H$^+$) calculated for $C_{35}H_{57}N_9O_6S_2$, 764.4, found; 764.7 [M+H]$^+$

HRMS (ESI$^+$): m/z (M+H$^+$) calculated for $C_{35}H_{57}N_9O_6S_2$, 764.3946, found; 764.3955 (-0.9 ppm error)

Analytical HPLC Rt: (220 nm) 12.110 min (94% Purity).

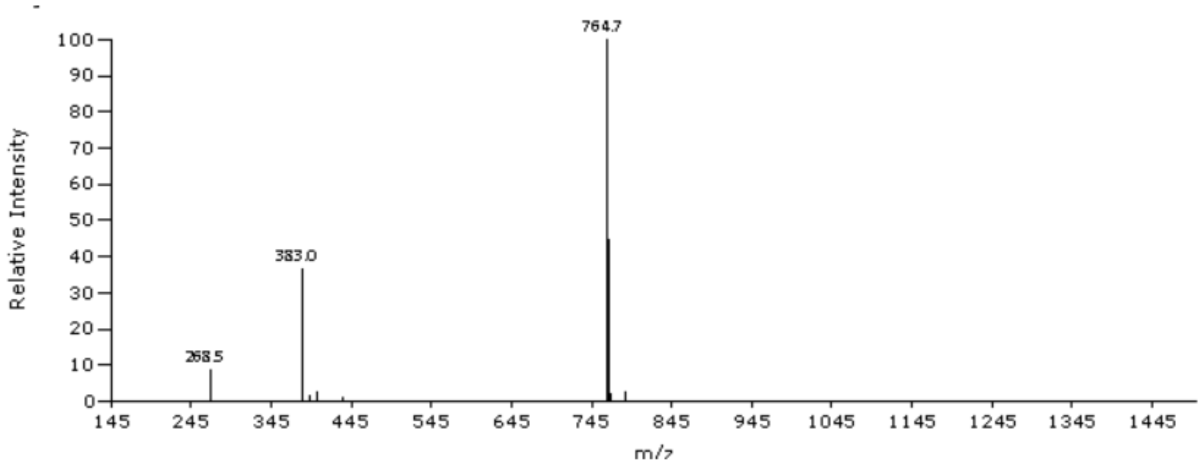

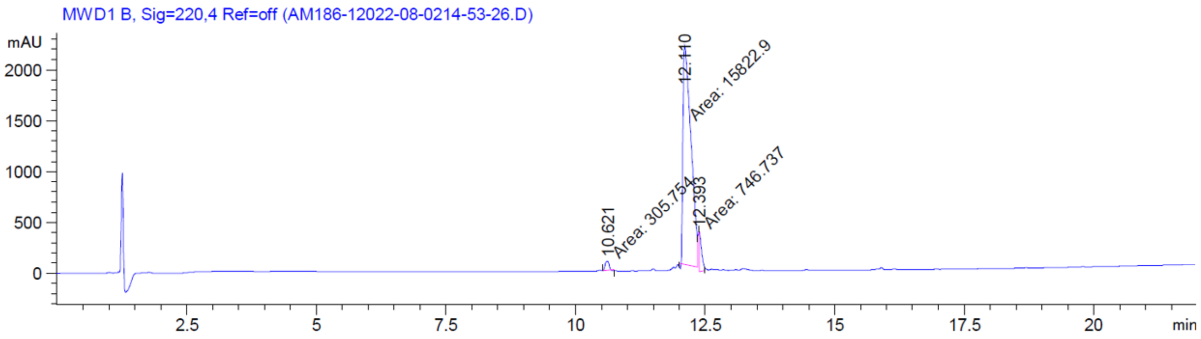

Peak #	RetTime [min]	Type	Width [min]	Area [mAU*s]	Height [mAU]	Area %
1	10.621	MM	0.0850	305.75378	59.92771	1.8118
2	12.110	MM	0.1793	1.58229e4	1471.11218	93.7632
3	12.393	MM	0.0467	746.73700	266.45160	4.4250

cyclo-CRLYIV

The linear peptide was synthesised using 2-Chlorotrityl chloride resin SPPS at 0.1 mmol scale. The linear peptide was then cyclised via HATU cyclisation conditions. The cyclic peptide crude was then subjected to TFA deprotection and was subsequently purified via reverse-phase HPLC chromatography to yield *cyclo*-CRLYIV as a white solid (7 mg, 0.009 mmol, 9%).

LR MS (ESI$^+$): m/z (M+H$^+$) calculated for $C_{35}H_{57}N_9O_7S$, 748.4, found; 748.6 [M+H]$^+$

HRMS (ESI$^+$): m/z (M+H$^+$) calculated for $C_{35}H_{57}N_9O_7S$, 748.4174, found; 748.4191 (-2.2 ppm error)

Analytical HPLC Rt: (220 nm) 11.131 mins (81.7% purity)

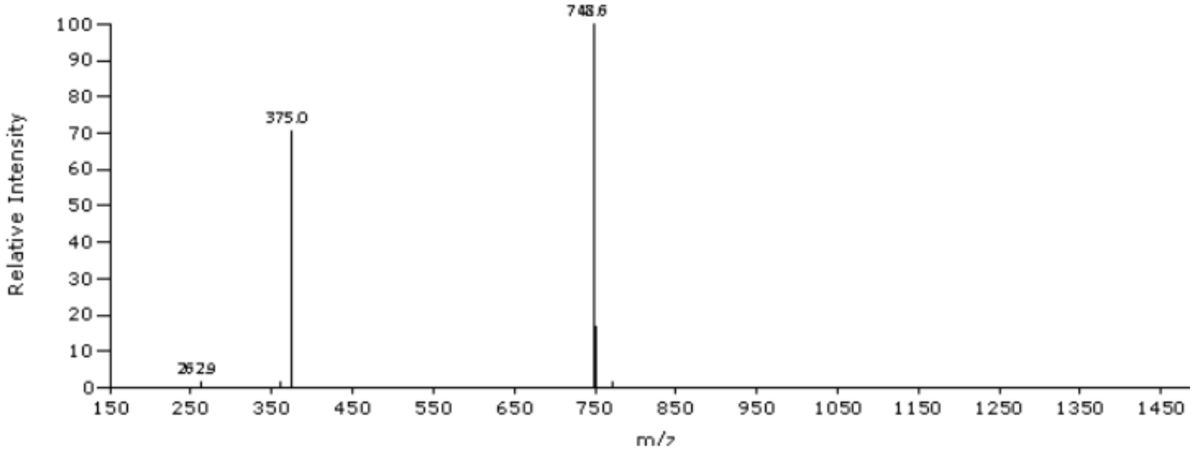

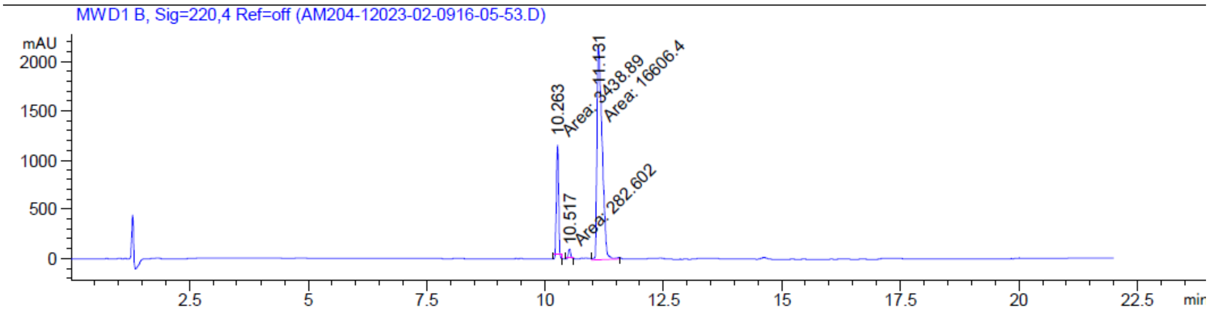

Peak #	RetTime [min]	Type	Width [min]	Area [mAU*s]	Height [mAU]	Area %
1	10.263	MM	0.0490	3438.89282	1169.64172	16.9171
2	10.517	MM	0.0517	282.60205	91.05397	1.3902
3	11.131	MM	0.1229	1.66064e4	2252.22437	81.6926

<u>*cyclo*-CRILIL</u>

The linear peptide was synthesised using 2-Chlorotrityl chloride resin SPPS at 0.1 mmol scale. The linear peptide was then cyclised via HATU cyclisation conditions. The cyclic peptide crude was then subjected to TFA deprotection and was subsequently purified via reverse-phase HPLC chromatography to yield *cyclo*-CRILIL as a white solid (15 mg, 0.02 mmol, 21%).

LR MS (ESI$^+$): m/z (M+H$^+$) calculated for $C_{33}H_{61}N_9O_6S$, 712.4, found; 712.5 $[M+H]^+$

HRMS (ESI$^+$): m/z (M+H$^+$) calculated for $C_{33}H_{61}N_9O_6S$, 712.4538, found; 712.4556 (-2.5 ppm error)

Analytical HPLC Rt: (220 nm) 12.290 min (100% Purity)

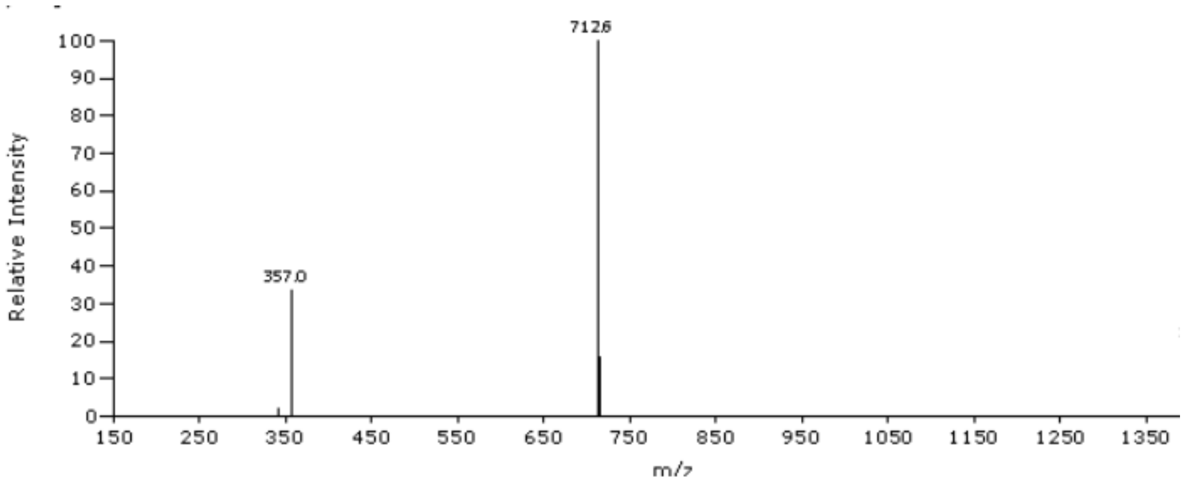

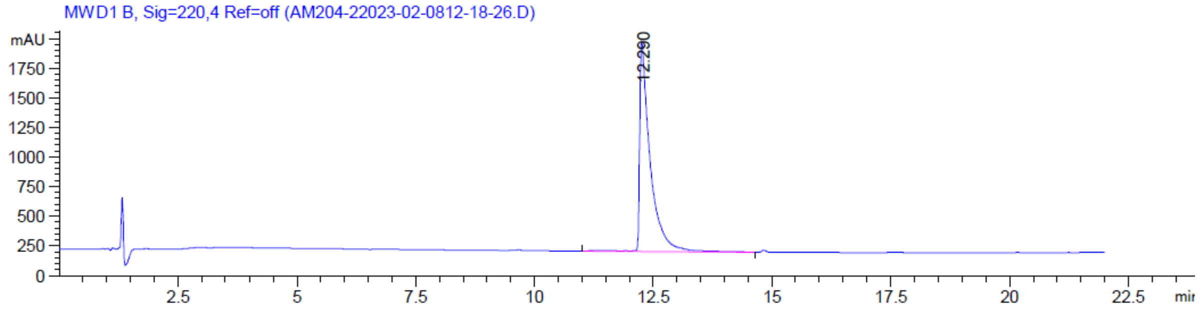

Peak #	RetTime [min]	Type	Width [min]	Area [mAU*s]	Height [mAU]	Area %
1	12.290	VB R	0.2285	2.70625e4	1674.52563	100.0000

cyclo-CRLYVL

The linear peptide was synthesised using 2-Chlorotrityl chloride resin SPPS at 0.1 mmol scale. The linear peptide was then cyclised via HATU cyclisation conditions. The cyclic peptide crude was then subjected to TFA deprotection and was subsequently purified via reverse-phase HPLC chromatography to yield *cyclo*-CRLYVL as a white solid (20 mg, 0.027 mmol, 27%).

LR MS (ESI$^+$): m/z (M+H$^+$) calculated for $C_{35}H_{57}N_9O_7S$, 748.41, found; 748.76 [M+H]$^+$

HRMS (ESI$^+$): m/z (M+H$^+$) calculated for $C_{35}H_{57}N_9O_7S$, 748.4174, found; 748.4156 (+2.4 ppm error)

Analytical HPLC Rt: (220 nm) 5.673 min (97.5% Purity).

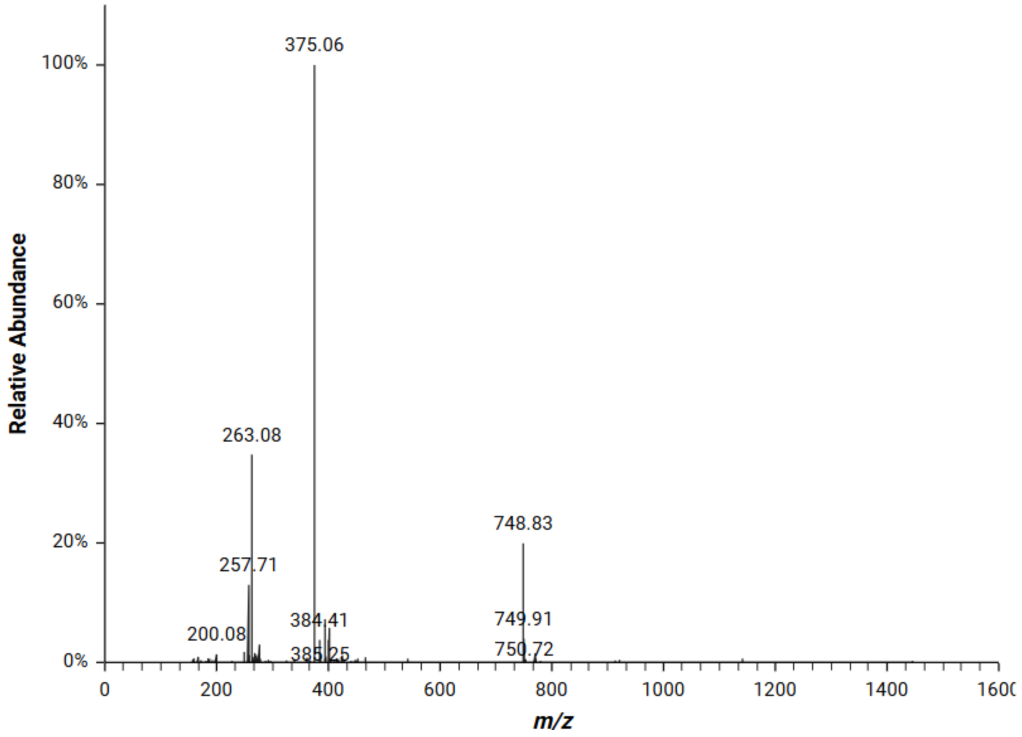

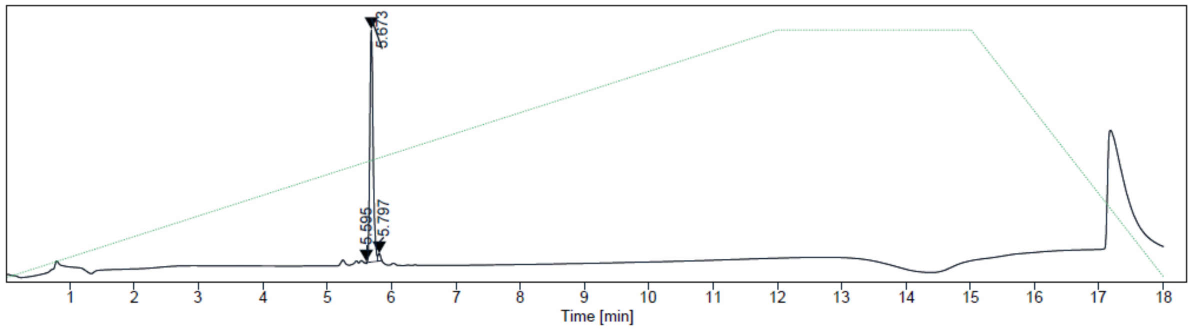

Signal: MWD1B,Sig=220,4 Ref=400,50

RT [min]	Type	Width [min]	Area	Height	Area%	Name
5.595	MM n	0.0324	4.6559	4.2491	0.1143	
5.673	MM m	0.1696	3967.7652	1100.6724	97.4488	
5.797	MM m	0.0688	99.2200	36.1122	2.4369	
		Sum	4071.6410			

<u>*cyclo*-CRLIIV</u>

The linear peptide was synthesised using 2-Chlorotrityl chloride resin SPPS at 0.1 mmol scale. The linear peptide was then cyclised via HATU cyclisation conditions. The cyclic peptide crude was then subjected to TFA deprotection and was subsequently purified via reverse-phase HPLC chromatography to yield *cyclo*-CRLIIV as a white solid (23 mg, 0.033 mmol, 33%).

LR MS (ESI$^+$): m/z (M+H$^+$) calculated for $C_{32}H_{59}N_9O_6S$, 698.4, found; 698.6 [M+H]$^+$

HRMS (ESI$^+$): m/z (M+H$^+$) calculated for $C_{32}H_{59}N_9O_6S$, 698.4382, found; 698.4393 (-1.6 ppm error)

Analytical HPLC Rt: (220 nm) 12.833 (94.3% purity)

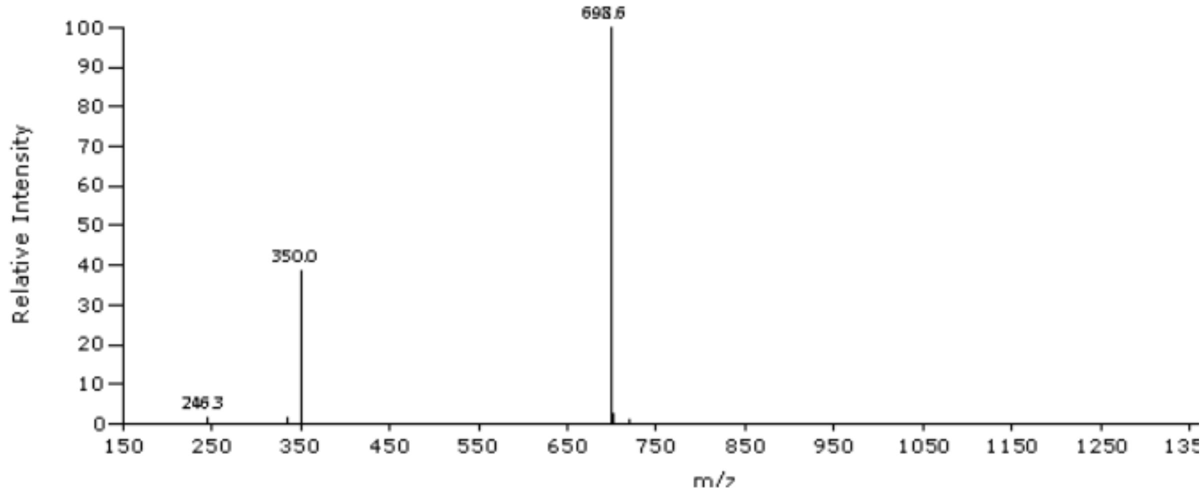

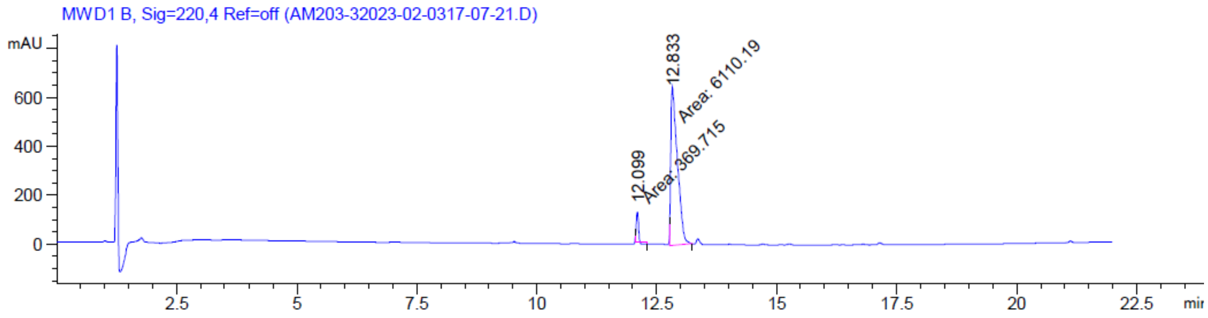

Peak #	RetTime [min]	Type	Width [min]	Area [mAU*s]	Height [mAU]	Area %
1	12.099	MM	0.0436	369.71545	141.44865	5.7056
2	12.833	MM	0.1540	6110.19043	661.28326	94.2944

<u>*cyclo*-CRVYIL</u>

The linear peptide was synthesised using 2-Chlorotrityl chloride resin SPPS at 0.1 mmol scale. The linear peptide was then cyclised via HATU cyclisation conditions. The cyclic peptide crude was then subjected to TFA deprotection and was subsequently purified via reverse-phase HPLC chromatography to yield *cyclo*-CRVYIL as a white solid (10 mg, 0.013 mmol, 13%).

LR MS (ESI$^+$): m/z (M+H$^+$) calculated for $C_{35}H_{57}N_9O_7S_2$, 748.4, found; 748.6 [M+H]$^+$

HRMS (ESI$^+$): m/z (M+H$^+$) calculated for $C_{35}H_{57}N_9O_7S_2$, 748.4162, found; 748.4174 (+1.7 ppm error)

Analytical HPLC Rt: (220 nm) 5.594 min (84.3% purity)

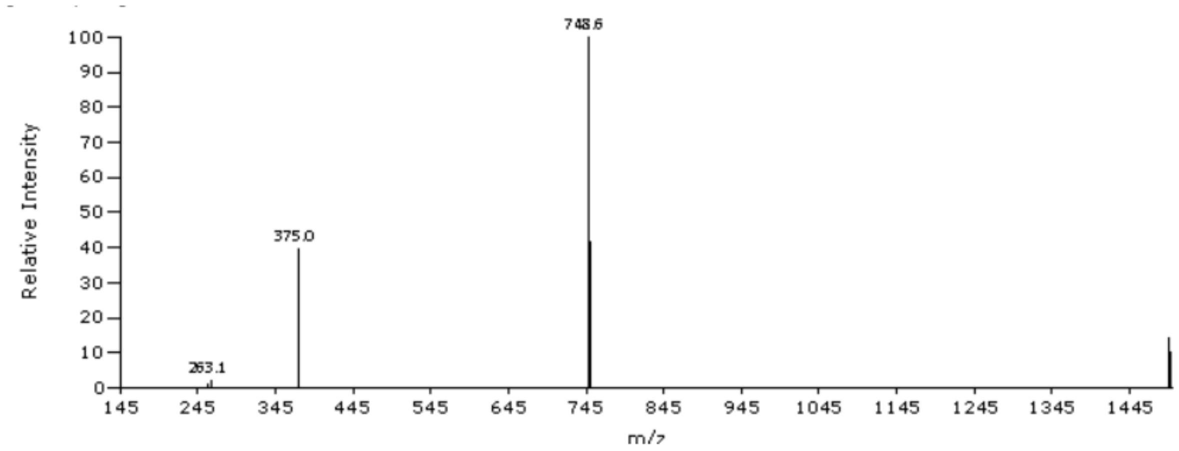

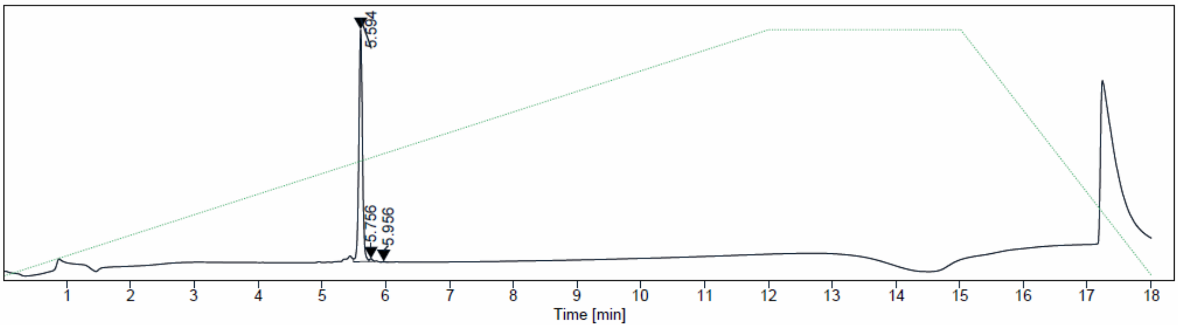

RT [min]	Type	Width [min]	Area	Height	Area%	Name
5.420	MM m	0.0933	120.4563	39.7581	3.8647	
5.676	MM m	0.0777	44.4898	16.0918	1.4274	
5.836	MM m	0.0747	325.9212	117.1201	10.4568	
5.919	MM m	0.1624	2625.9715	740.3585	84.2511	
		Sum	3116.8388			

<u>*cyclo*-CVMRIA</u>

The linear peptide was synthesised using 2-Chlorotrityl chloride resin SPPS at 0.1 mmol scale. The linear peptide was then cyclised via HATU cyclisation conditions. The cyclic peptide crude was then subjected to TFA deprotection and was subsequently purified via reverse-phase HPLC chromatography to yield *cyclo*-CVMRIA as a white solid (8 mg, 0.012 mmol, 12%).

LR MS (ESI$^+$): m/z (M+H$^+$) calculated for $C_{28}H_{51}N_9O_6S_2$, 674.3, found; 674.5 [M+H]$^+$

HRMS (ESI$^+$): m/z (M+H$^+$) calculated for $C_{28}H_{51}N_9O_6S_2$, 674.3476 found; 674.3467 (+1.4 ppm error)

Analytical HPLC Rt: (220 nm) 4.637 min (99.5% purity)

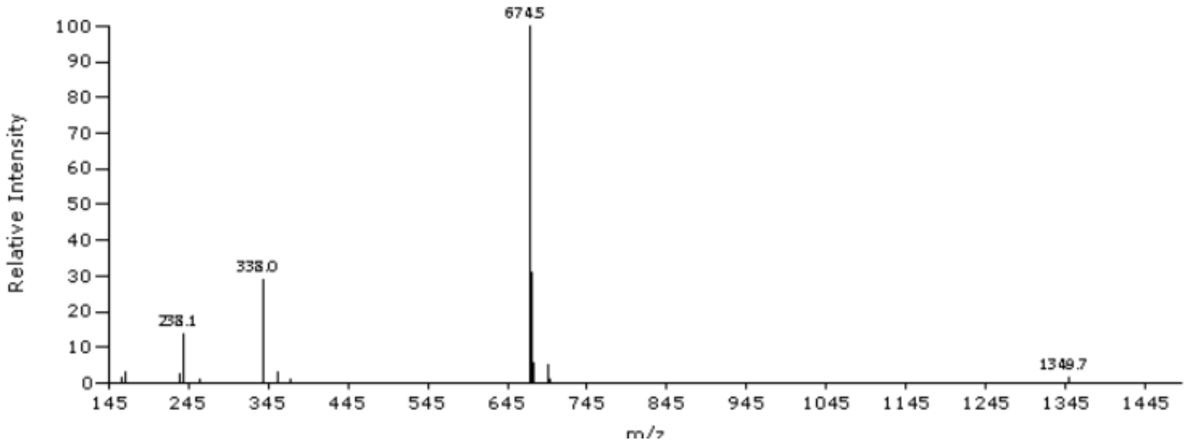

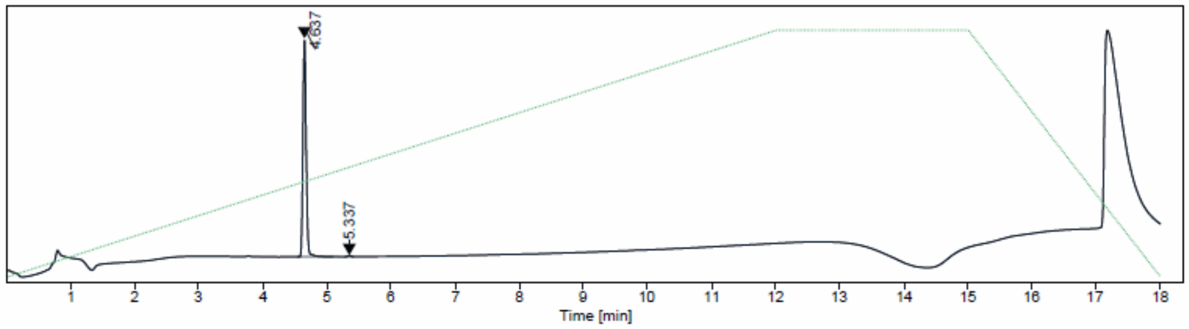

Signal: MWD1B,Sig=220,4 Ref=400,50

RT [min]	Type	Width [min]	Area	Height	Area%	Name
4.637	BB	0.5578	2126.0153	604.8485	99.4976	
5.337	BB	0.1518	10.7355	3.2379	0.5024	
		Sum	2136.7507			

cyclo-CRTIVL

The linear peptide was synthesised using 2-Chlorotrityl chloride resin SPPS at 0.1 mmol scale. The linear peptide was then cyclised via HATU cyclisation conditions. The cyclic peptide crude was then subjected to TFA deprotection and was subsequently purified via reverse-phase HPLC chromatography to yield *cyclo*-CRTIVL as a white solid (3 mg, 0.004 mmol, 4%).

LR MS (ESI$^+$): m/z (M+H$^+$) calculated for $C_{30}H_{55}N_9O_7S$, 686.4, found; 686.6 [M+H]$^+$

HRMS (ESI$^+$): m/z (M+H$^+$) calculated for $C_{30}H_{55}N_9O_6S_2$, 686.4018, found; 686.4001 (+2.5 ppm error)

Analytical HPLC Rt: (220 nm) 5.223 min (72.2% purity)

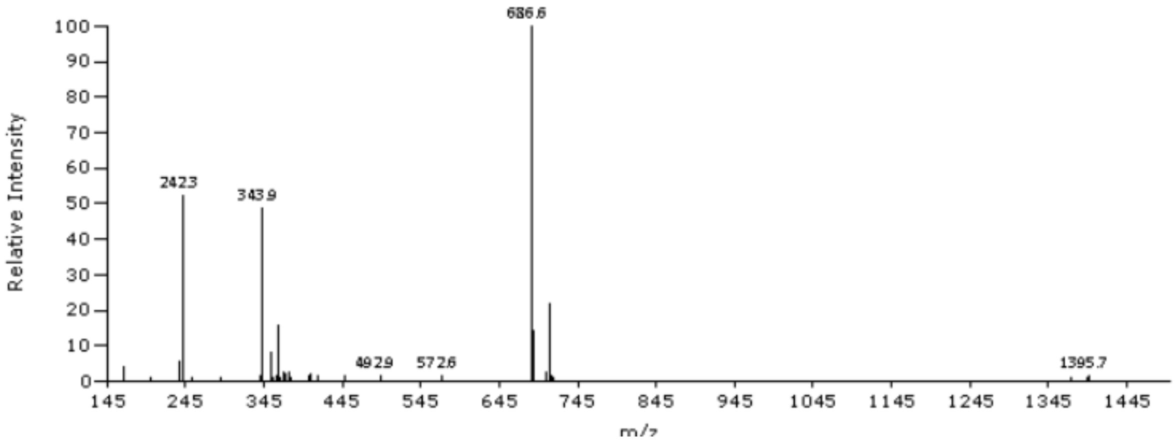

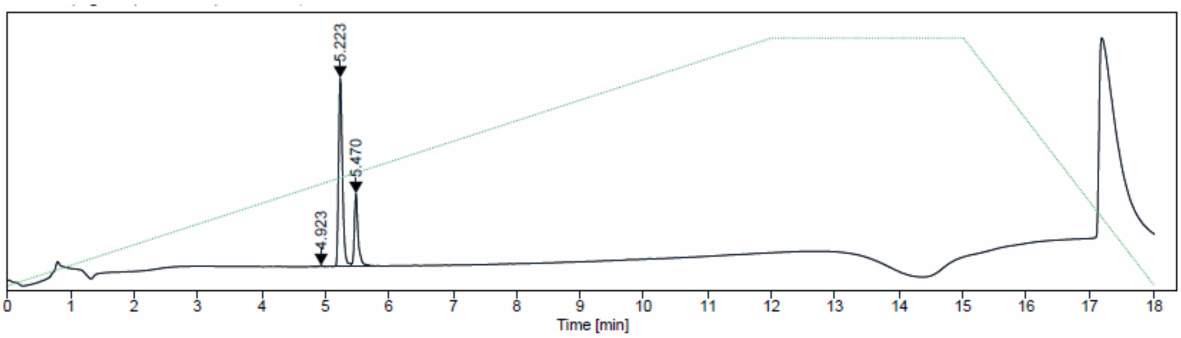

Signal: MWD1B,Sig=220,4 Ref=400,50

RT [min]	Type	Width [min]	Area	Height	Area%	Name
4.923	BB	0.2080	10.8087	2.8124	0.3460	
5.223	BV	0.2932	2256.6071	535.6951	72.2354	
5.470	VB	0.3735	856.5474	206.1284	27.4186	
		Sum	3123.9632			

cyclo-CRLLIL

The linear peptide was synthesised using 2-Chlorotrityl chloride resin SPPS at 0.1 mmol scale. The linear peptide was then cyclised via HATU cyclisation conditions. The cyclic peptide crude was then subjected to TFA deprotection and was subsequently purified via reverse-phase HPLC chromatography to yield *cyclo*-CRLLIL as a white solid (21 mg, 0.031 mmol, 31%).

LR MS (ESI$^+$): m/z (M+H$^+$) calculated for $C_{33}H_{61}N_9O_6S$, 712.4, found; 712.6 [M+H]$^+$

HRMS (ESI$^+$): m/z (M+H$^+$) calculated for $C_{33}H_{61}N_9O_6S$, 712.4538, found; 712.4533 (+1.7 ppm error)

Analytical HPLC Rt: (220 nm) 5.223 min (93.9% purity)

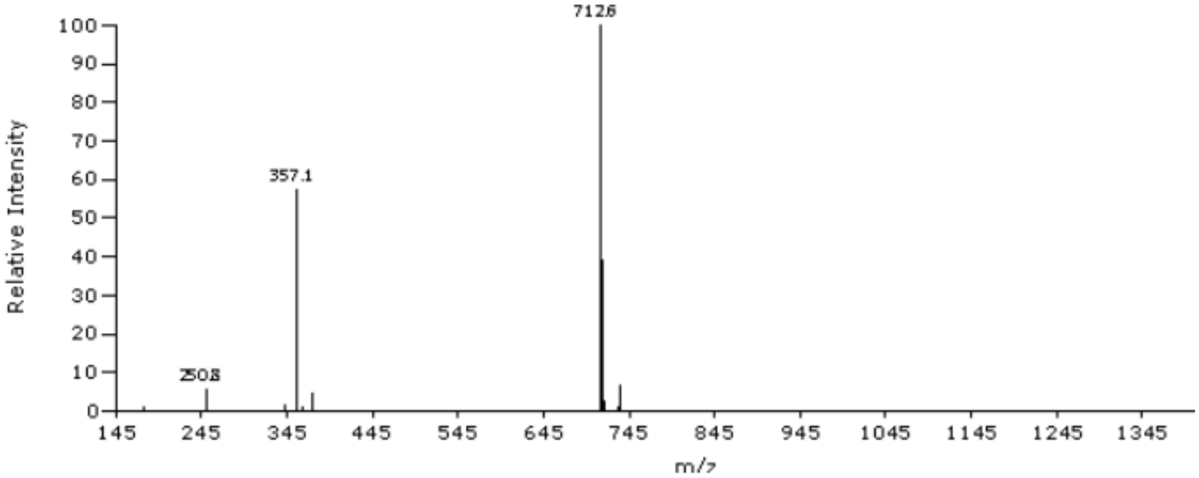

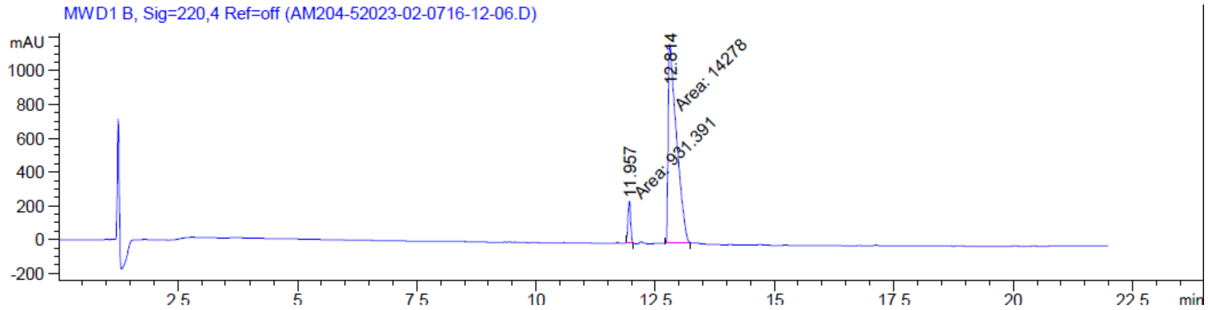

Peak #	RetTime [min]	Type	Width [min]	Area [mAU*s]	Height [mAU]	Area %
1	11.957	MM	0.0645	931.39056	240.74300	6.1238
2	12.814	MM	0.1984	1.42780e4	1199.48767	93.8762

<u>*cyclo*-CRRIIL</u>

The linear peptide was synthesised using 2-Chlorotrityl chloride resin SPPS at 0.1 mmol scale. The linear peptide was then cyclised via HATU cyclisation conditions. The cyclic peptide crude was then subjected to TFA deprotection and was subsequently purified via reverse-phase HPLC chromatography to yield *cyclo*-CRRIIL as a white solid (8 mg, 0.011 mmol, 11%).

LR MS (ESI$^+$): m/z (M+H$^+$) calculated for $C_{33}H_{62}N_{12}O_6S$, 755.5, found; 755.6 $[M+H]^+$

HRMS (ESI$^+$): m/z (M+H$^+$) calculated for $C_{33}H_{62}N_{12}O_6S$, 755.4709 found; 755.4705 (+1.7 ppm error)

Analytical HPLC Rt: (220 nm) 10.950 min (100% purity)

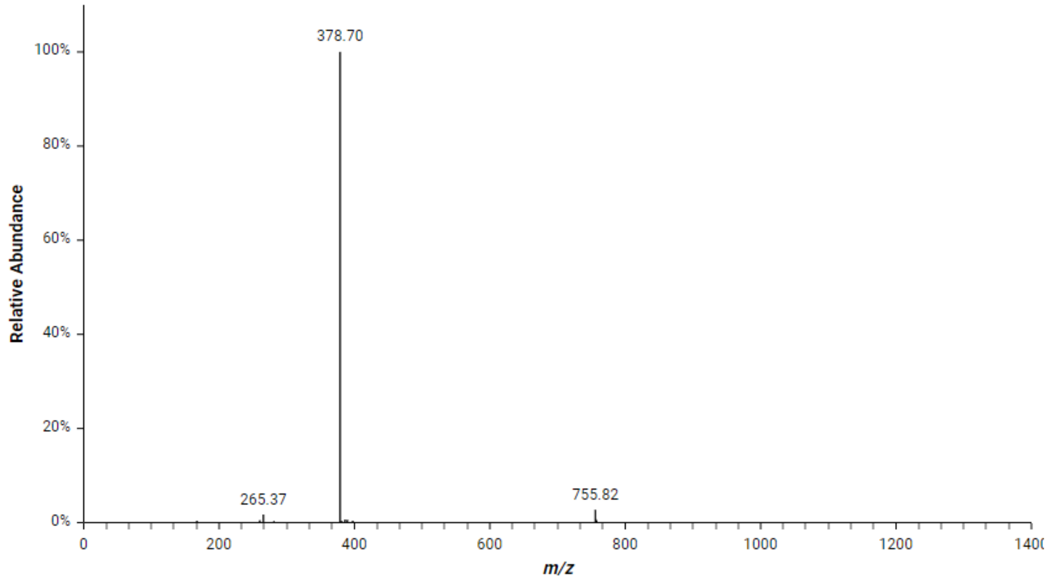

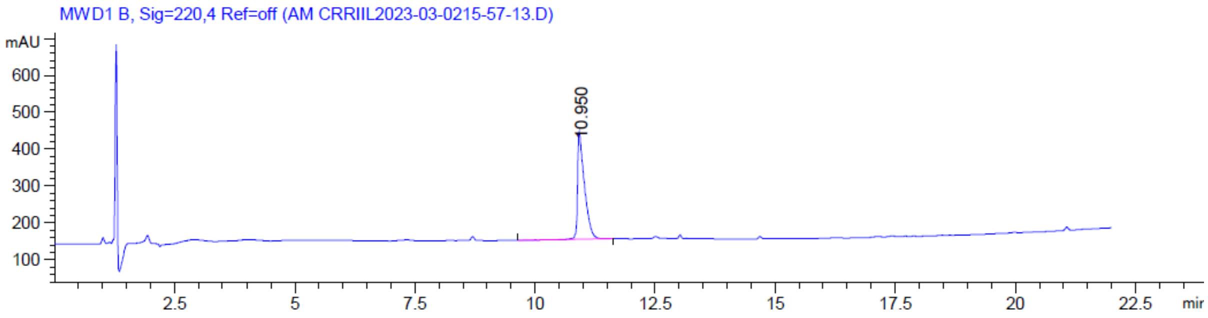

Peak #	RetTime [min]	Type	Width [min]	Area [mAU*s]	Height [mAU]	Area %
1	10.950	BB	0.1718	2942.91577	260.50363	100.0000

cyclo-CRMFIL

The linear peptide was synthesised using 2-Chlorotrityl chloride resin SPPS at 0.1 mmol scale. The linear peptide was then cyclised via HATU cyclisation conditions. The cyclic peptide crude was then subjected to TFA deprotection and was subsequently purified via reverse-phase HPLC chromatography to yield *cyclo*-CRMFIL as a white solid (6.7 mg, 0.0088 mmol, 9%).

LR MS (ESI$^+$): m/z (M+H$^+$) calculated for $C_{35}H_{57}N_9O_6S_2$, 764.4, found; 764.5 $[M+H]^+$

HRMS (ESI$^+$): m/z (M+H$^+$) calculated for $C_{35}H_{57}N_9O_6S_2$, 764.3946 found; 764.3953 (-1.0 ppm error)

Analytical HPLC Rt: (220 nm) 13.242 min (93% purity)

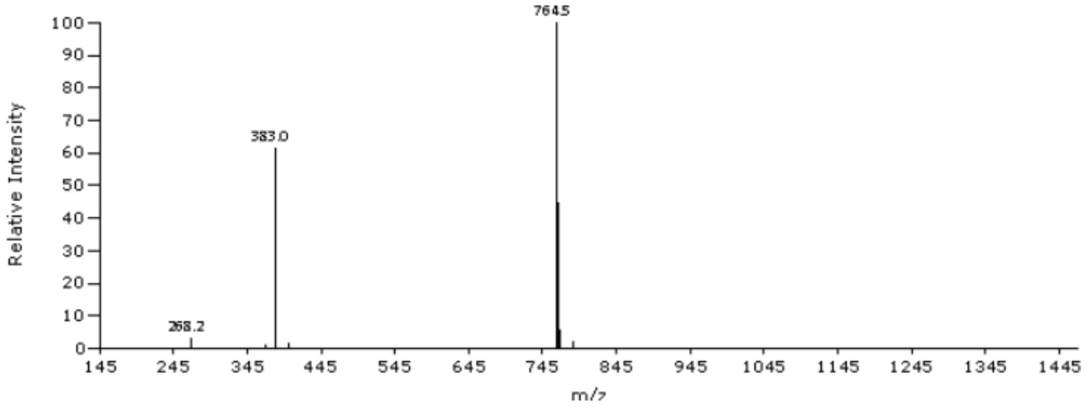

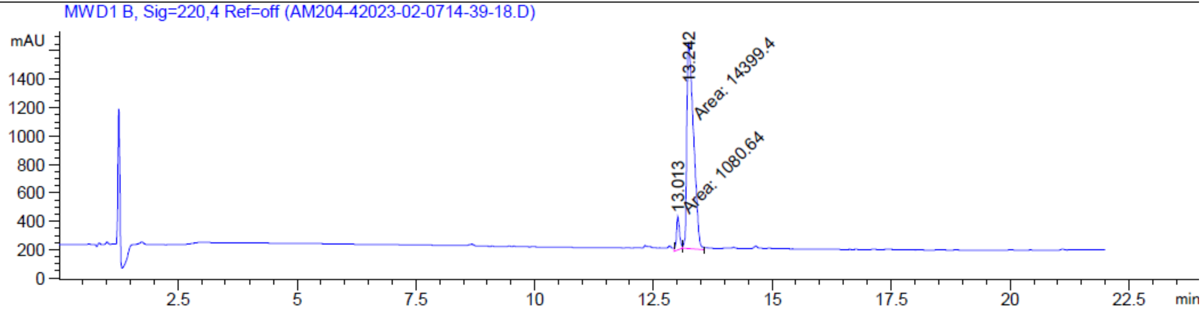

Peak #	RetTime [min]	Type	Width [min]	Area [mAU*s]	Height [mAU]	Area %
1	13.013	MM	0.0773	1080.63586	233.12259	6.9809
2	13.242	MM	0.1657	1.43994e4	1448.52893	93.0191

cyclo-CRYLIL

The linear peptide was synthesised using 2-Chlorotrityl chloride resin SPPS at 0.1 mmol scale. The linear peptide was then cyclised via HATU cyclisation conditions. The cyclic peptide crude was then subjected to TFA deprotection and was subsequently purified via reverse-phase HPLC chromatography to yield *cyclo*-CRYLIL as a white solid (31 mg, 0.041 mmol, 41%)

LR MS (ESI$^+$): m/z (M+H$^+$) calculated for $C_{36}H_{59}N_9O_7S$, 762.4, found; 762.7

HRMS (ESI$^+$): m/z (M+H$^+$) calculated for $C_{36}H_{59}N_9O_7S$, 762.4331, found; 762.4327 (-0.5 ppm error).

Analytical HPLC Rt: (220 nm) 12.132 min (98% Purity)

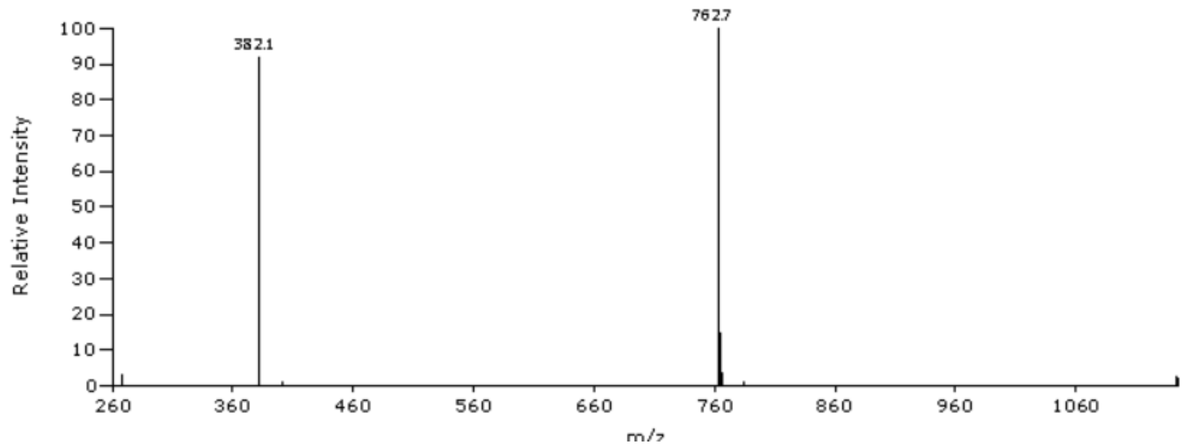

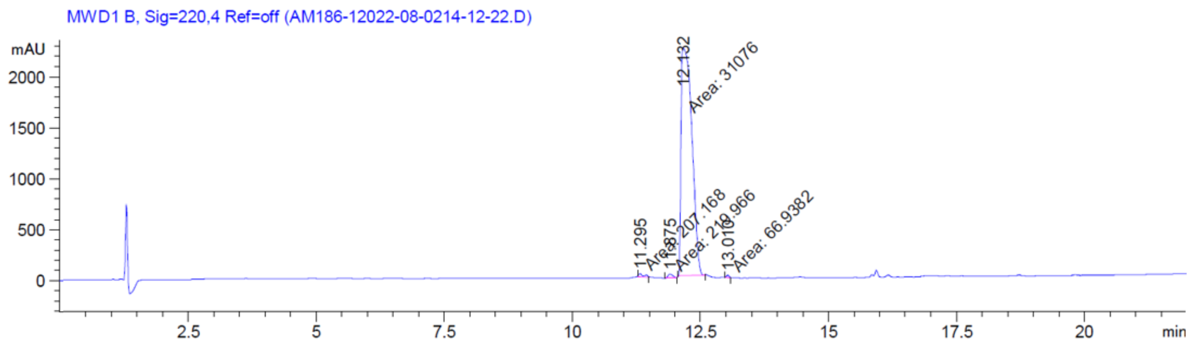

Peak #	RetTime [min]	Type	Width [min]	Area [mAU*s]	Height [mAU]	Area %
1	11.295	MM	0.1168	207.16753	29.55183	0.6562
2	11.875	MM	0.1054	219.96609	34.78299	0.6968
3	12.132	MM	0.2460	3.10760e4	2105.02051	98.4350
4	13.010	MM	0.0561	66.93819	19.89194	0.2120

cyclo-CRVLVL

The linear peptide was synthesised using 2-Chlorotrityl chloride resin SPPS at 0.1 mmol scale. The linear peptide was then cyclised via HATU cyclisation conditions. The cyclic peptide crude was then subjected to TFA deprotection and was subsequently purified via reverse-phase HPLC chromatography to yield *cyclo*-CRVLVL as a white solid (20 mg, 0.029 mmol, 29%)

LR MS (ESI$^+$): m/z (M+H$^+$) calculated for $C_{31}H_{57}N_9O_6S$, 684.4, found; 684.6

HRMS (ESI$^+$): m/z (M+H$^+$) calculated for $C_{31}H_{57}N_9O_6S$, 684.4225, found; 684.4234 (-0.5 ppm error).

Analytical HPLC Rt: (220 nm) 11.675 (95.8% purity)

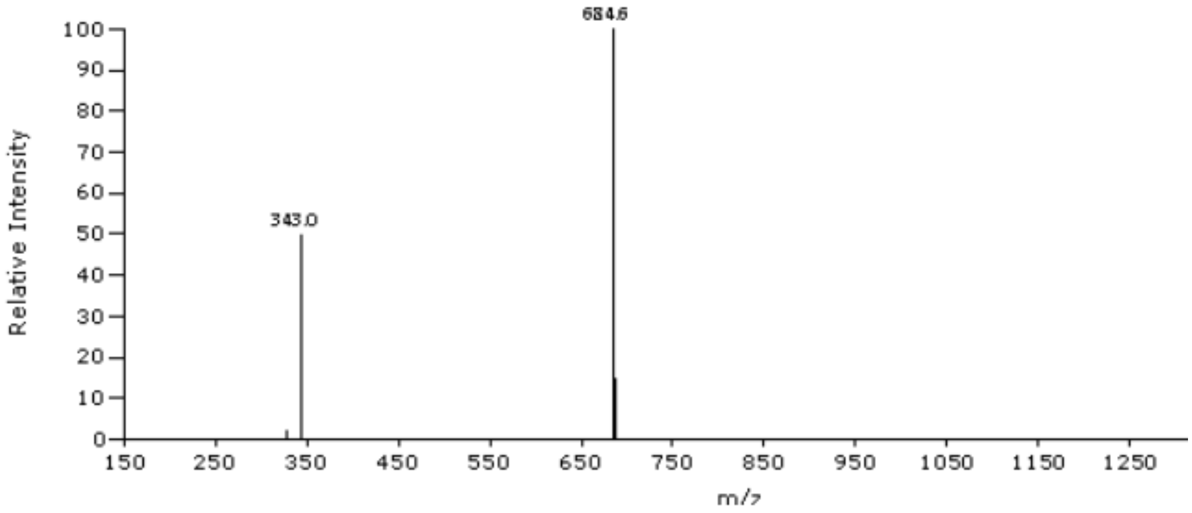

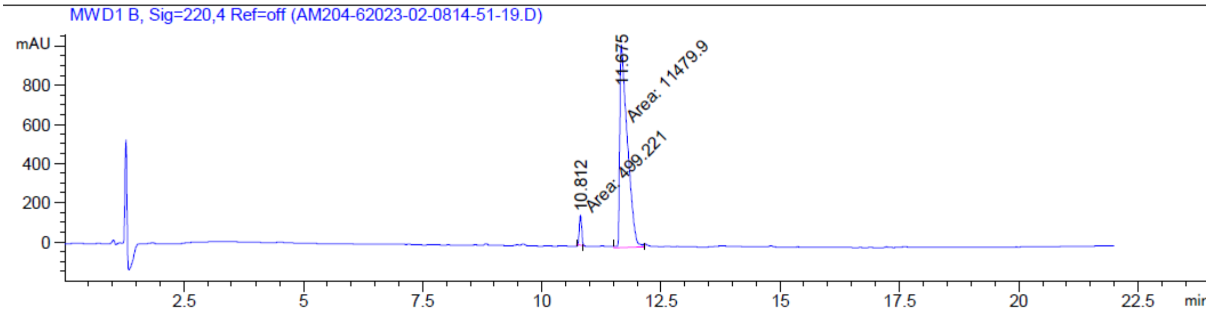

Peak #	RetTime [min]	Type	Width [min]	Area [mAU*s]	Height [mAU]	Area %
1	10.812	MM	0.0557	499.22067	149.27106	4.1674
2	11.675	MM	0.1873	1.14799e4	1021.53070	95.8326

cyclo-CRMYVL

The linear peptide was synthesised using 2-Chlorotrityl chloride resin SPPS at 0.1 mmol scale. The linear peptide was then cyclised via HATU cyclisation conditions. The cyclic peptide crude was then subjected to TFA deprotection and was subsequently purified via reverse-phase HPLC chromatography to yield *cyclo*-CRMYVL as a white solid (15 mg, 0.020 mmol, 20%)

LR MS (ESI$^+$): m/z (M+H$^+$) calculated for $C_{34}H_{55}N_9O_7S_2$, 766.4, found; 766.6

HRMS (ESI$^+$): m/z (M+H$^+$) calculated for $C_{34}H_{55}N_9O_7S_2$, 766.3720 found; 766.3739 (+2.5 ppm error).

Analytical HPLC Rt: (220 nm) 5.561 min (96% Purity).

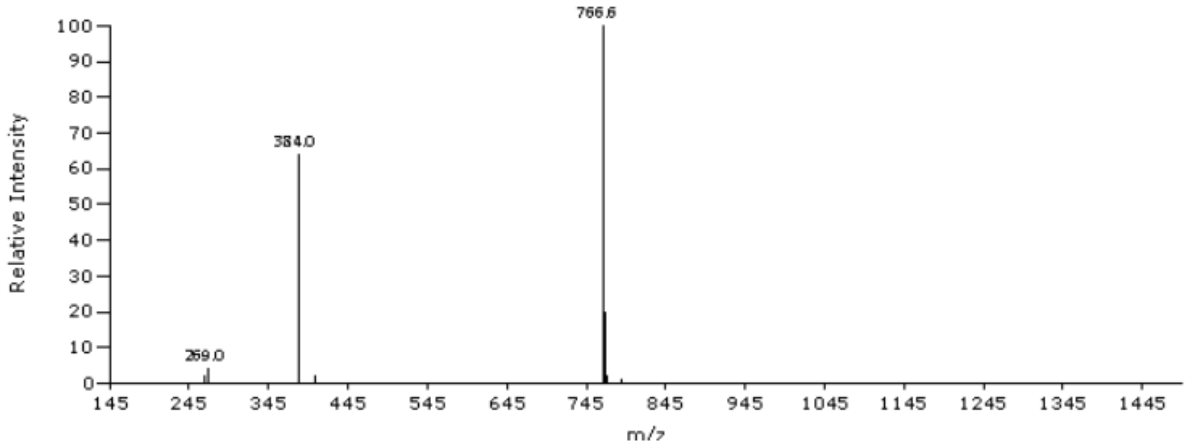

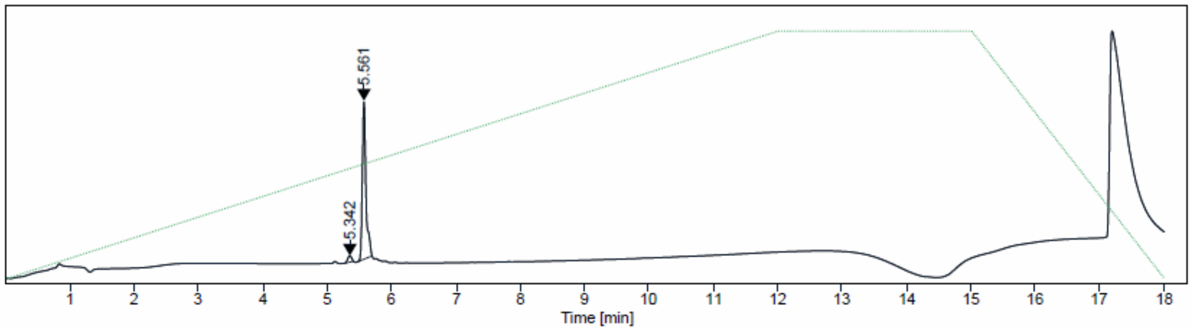

Signal: MWD1B,Sig=220,4 Ref=400,50

RT [min]	Type	Width [min]	Area	Height	Area%	Name
5.342	MM m	0.1356	74.6896	18.2219	3.9593	
5.561	MM m	0.2316	1811.7563	464.7544	96.0407	
		Sum	1886.4459			

cyclo-CRYYVL

The linear peptide was synthesised using 2-Chlorotrityl chloride resin SPPS at 0.1 mmol scale. The linear peptide was then cyclised via HATU cyclisation conditions. The cyclic peptide crude was then subjected to TFA deprotection and was subsequently purified via reverse-phase HPLC chromatography to yield *cyclo*-CRYYVL as a white solid (4 mg, 0.005 mmol, 5%)

LR MS (ESI$^+$): m/z (M+H$^+$) calculated for $C_{38}H_{55}N_9O_8S$, 798.4, found; 798.6

HRMS (ESI$^+$): m/z (M+H$^+$) calculated for $C_{38}H_{55}N_9O_8S$, 798.3967 found; 798.3985 (-2.2 ppm error).

Analytical HPLC Rt: (220 nm) 5.500 min (77.2% Purity).

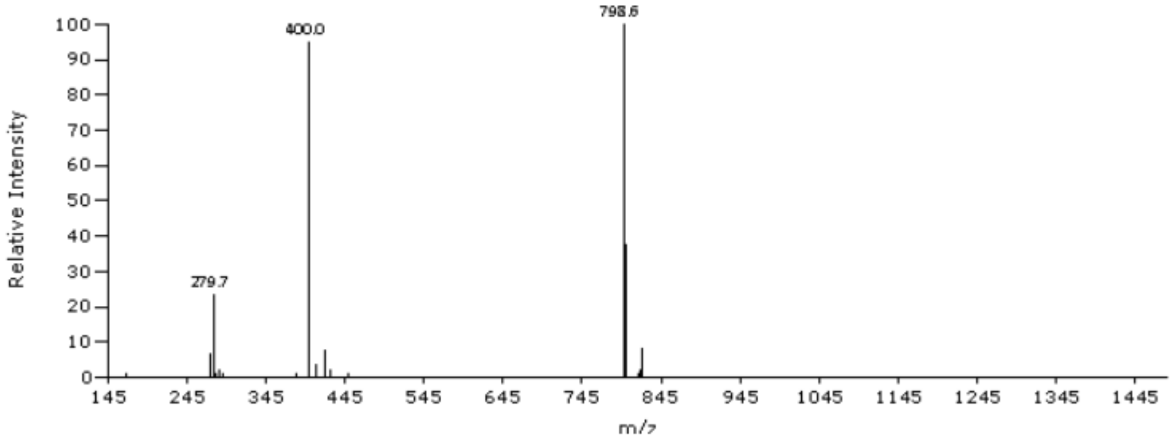

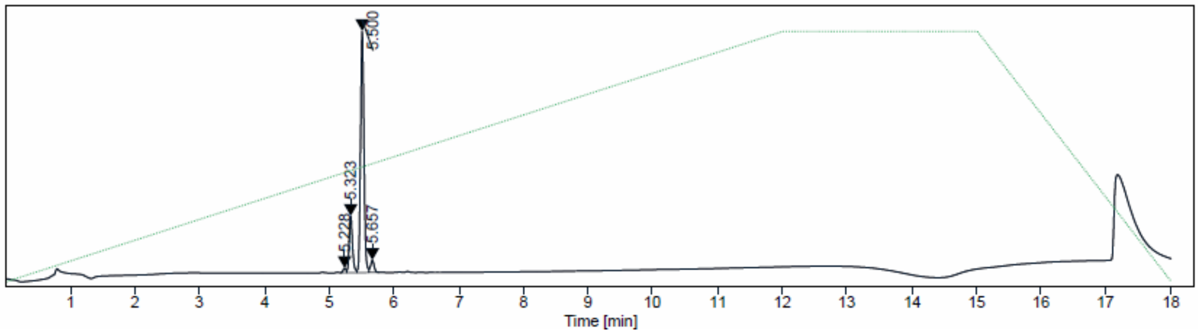

Signal: MWD1B,Sig=220,4 Ref=400,50

RT [min]	Type	Width [min]	Area	Height	Area%	Name
5.228	BV	0.0968	86.0330	27.4913	1.1723	
5.323	VV	0.1541	1287.2909	361.2168	17.5407	
5.500	VV	0.1864	5664.0611	1543.1539	77.1789	
5.657	VB	0.1628	301.4851	77.7705	4.1081	
		Sum	7338.8700			

<u>*cyclo*-CLLFCL</u>

The linear peptide was synthesised using 2-Chlorotrityl chloride resin SPPS at 0.1 mmol scale. The linear peptide was then cyclised via HATU cyclisation conditions. The cyclic peptide crude was then subjected to TFA deprotection and was subsequently purified via reverse-phase HPLC chromatography to yield *cyclo*-CLLFCL as a white solid (31 mg, 0.045 mmol, 45%).

LR MS (ESI$^+$): m/z (M+H$^+$) calculated for $C_{33}H_{52}N_6O_6S_2$, 693.3, found; 693.7 [M+H]$^+$

HRMS (ESI$^+$): m/z (M+H$^+$) calculated for $C_{33}H_{52}N_6O_6S_2$, 693.3463, found; 693.3470 (-1.0 ppm error)

Analytical HPLC Rt: (220 nm) 16.080 min (97% Purity).

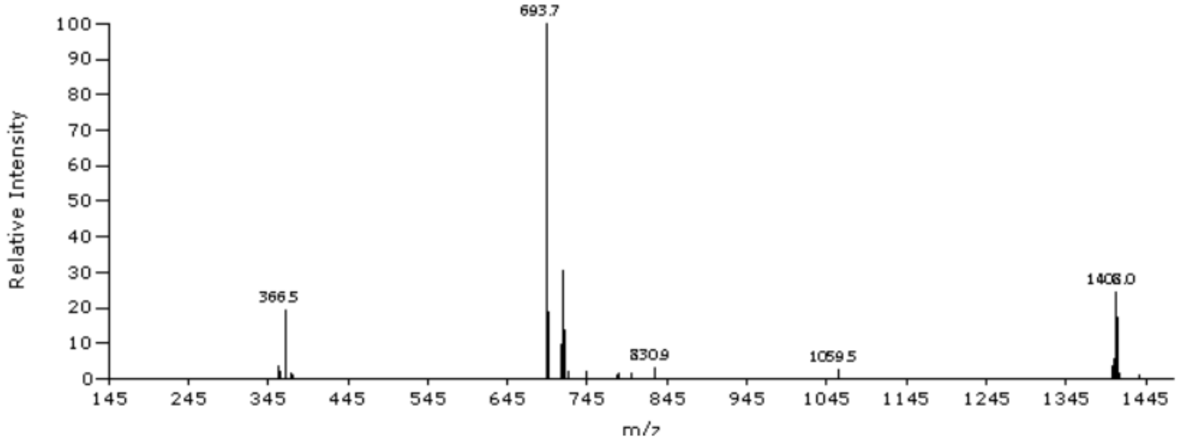

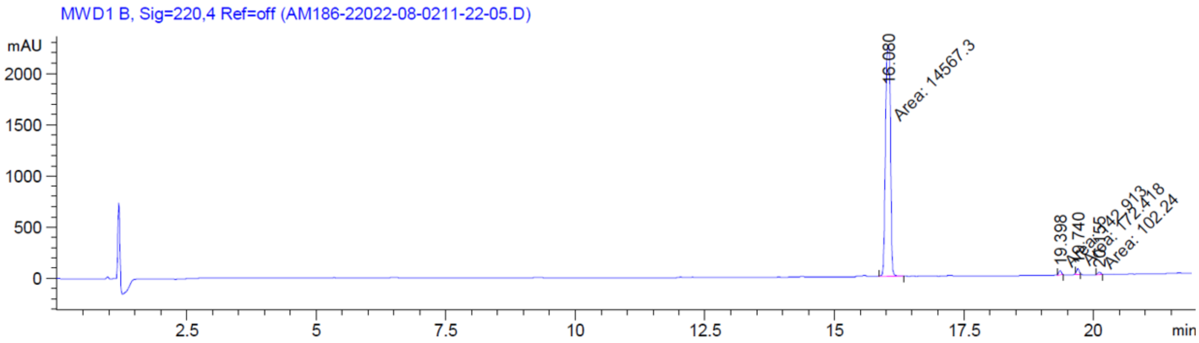

Peak #	RetTime [min]	Type	Width [min]	Area [mAU*s]	Height [mAU]	Area %
1	16.080	MM	0.1115	1.45673e4	2178.10986	97.2134
2	19.398	MM	0.0576	142.91286	41.37557	0.9537
3	19.740	MM	0.0553	172.41776	51.97831	1.1506
4	20.155	MM	0.0667	102.24026	25.54376	0.6823

cyclo-C(TAT)RTYIL

Cyclo - CRTYIL (4 mg, 0.0053 mmol, 1 eq) was added to a solution of C(Spy)GRKKRRQRRRPPQ (20 mg, 0.0106 mmol, 2 eq) dissolved in DMF (2 mL) and stirred in an inert atmosphere overnight at ambient temperature. The mixture was then purified by reverse-phase HPLC. Fractions analysed via LC/MS, desired fractions were then pooled, concentrated *in vacuo*, and lyophilised to yield *Cyclo*-C(TAT)RTYIL as a white solid (9.7 mg, 0.0038 mmol, 71 %).

LR MS (ESI$^+$): m/z (M+4H)4+ calculated for $C_{107}H_{189}N_{45}O_{25}S_2$, 514.7, found; 515.0 $[M+4H]4^+$

HRMS (ESI$^+$): shows expected model isotope pattern.

Analytical HPLC Rt: (220 nm) 7.848 min (88% Purity).

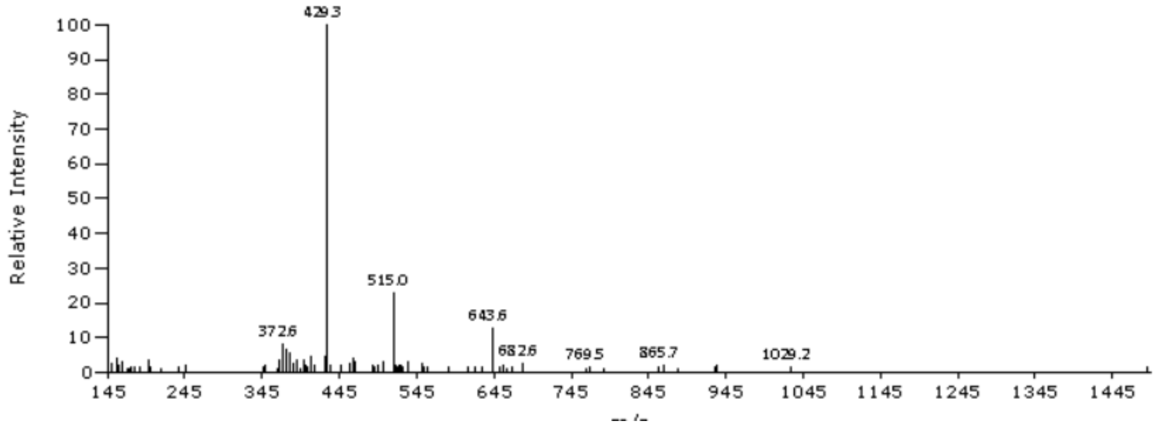

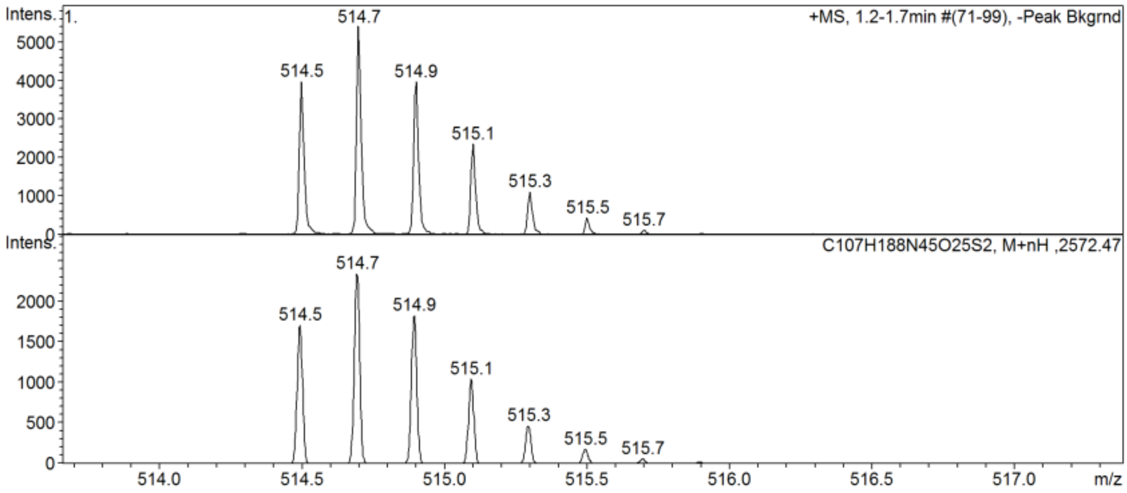

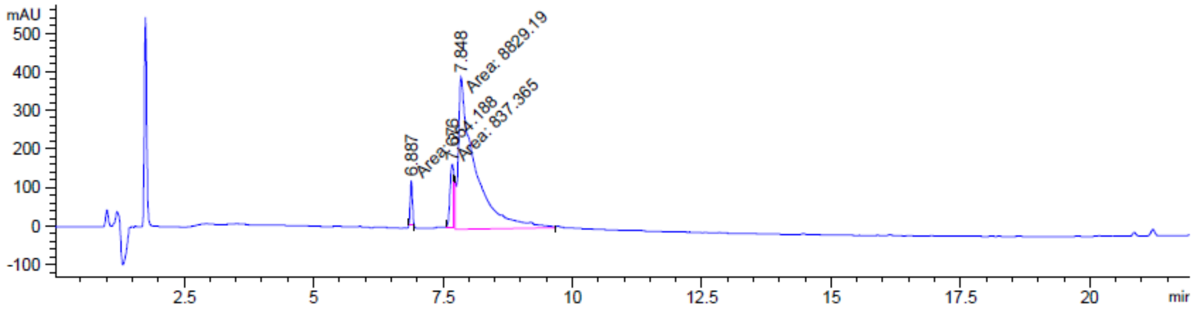

Peak #	RetTime [min]	Type	Width [min]	Area [mAU*s]	Height [mAU]	Area %
1	6.887	MM	0.0514	354.18774	114.75957	3.5345
2	7.676	MM	0.0856	837.36505	163.01120	8.3563
3	7.848	MM	0.3736	8829.19043	393.89941	88.1091

<u>*cyclo*-C(TAT)RILIL</u>

Cyclo – CRILIL (5 mg, 0.007 mmol, 1 eq) was added to a solution of C(Spy)GRKKRRQRRRPPQ (27 mg, 0.014 mmol, 2 eq) dissolved in DMF (2 mL) and stirred in an inert atmosphere overnight at ambient temperature. The mixture was then purified by reverse-phase HPLC. Fractions analysed via LC/MS, desired fractions were then pooled, concentrated *in vacuo*, and lyophilised to yield *Cyclo*-C(TAT)RILIL as a white solid (11.4 mg, 0.0045 mmol, 64 %).

LR MS (ESI$^+$): m/z (M+6H)6+ calculated for $C_{106}H_{195}N_{45}O_{23}S_2$, 422.8, found; 423.2 $[M+6H]^{6+}$

HRMS (ESI$^+$): shows expected model isotope pattern.

Analytical HPLC Rt: (220 nm) 9.830 min (95.8% Purity).

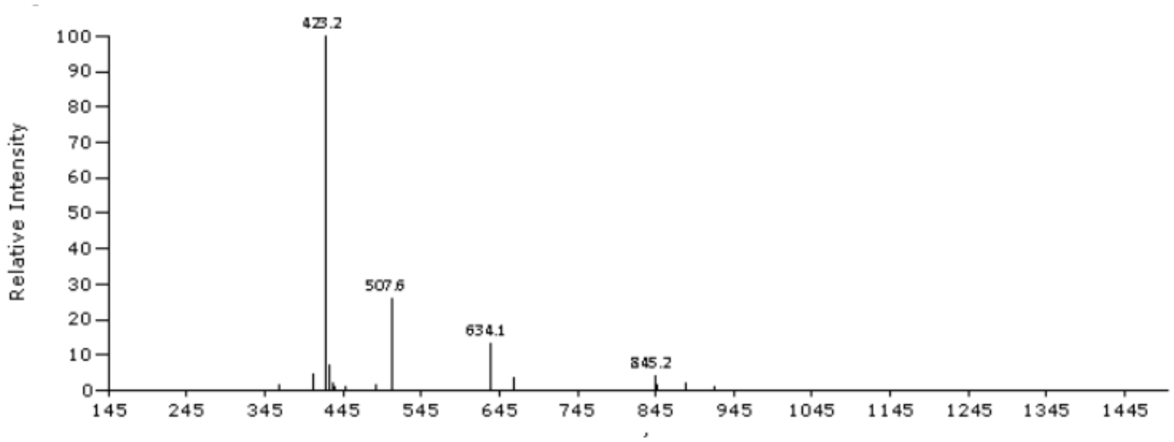

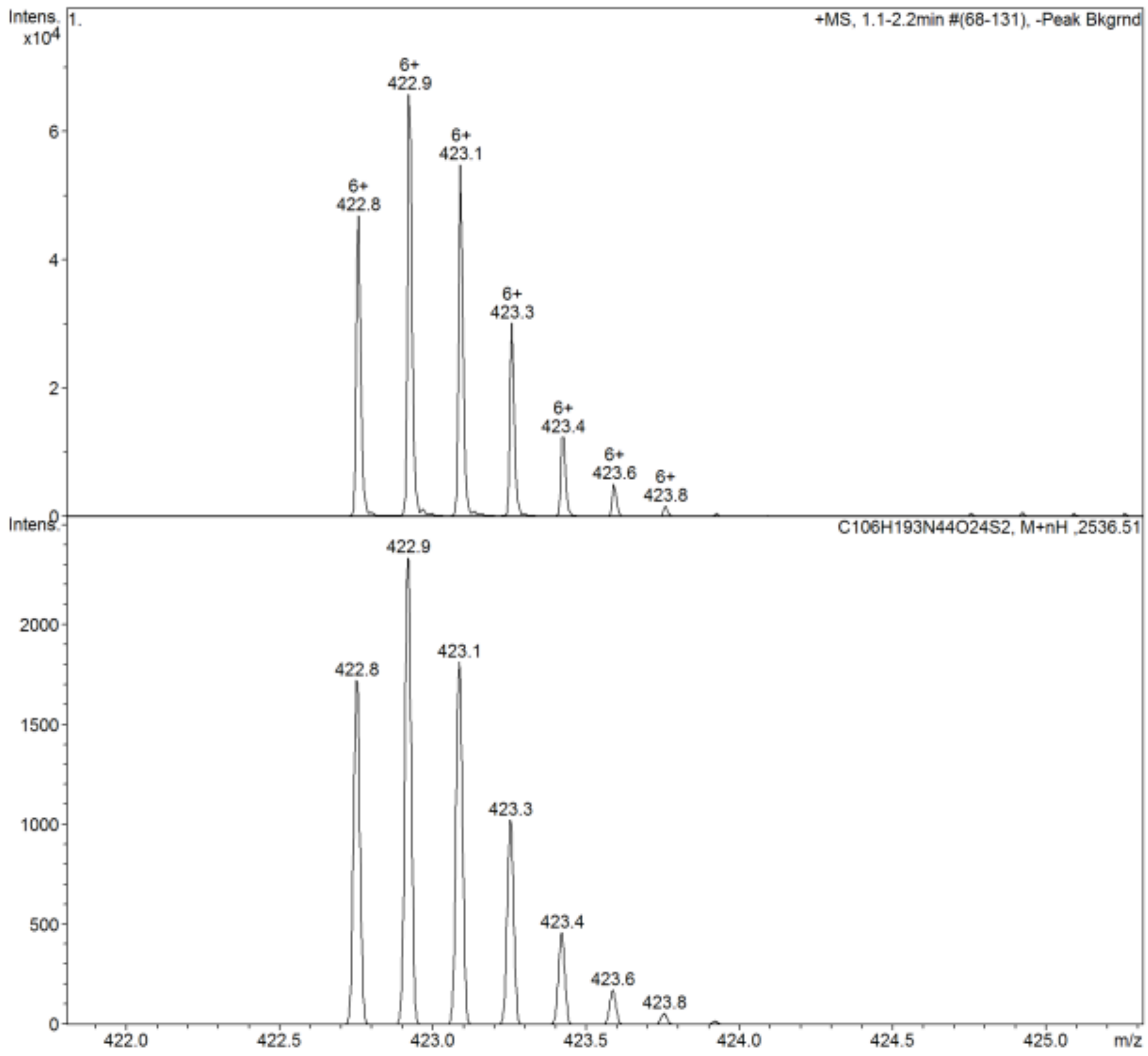

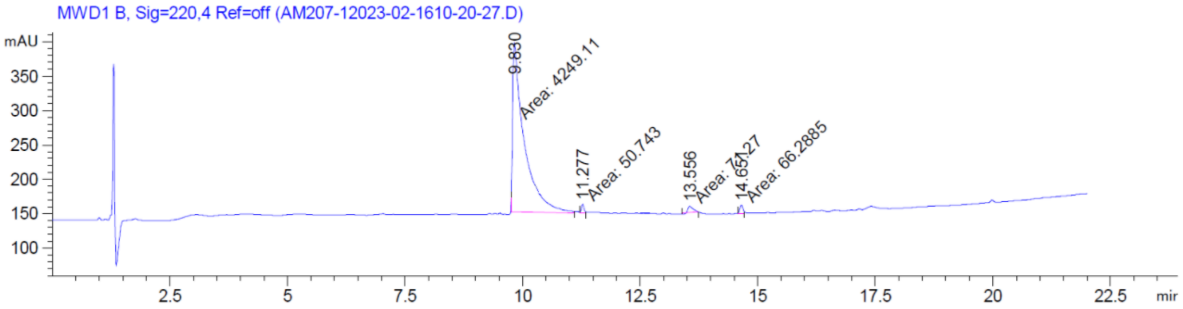

Peak #	RetTime [min]	Type	Width [min]	Area [mAU*s]	Height [mAU]	Area %
1	9.830	MM	0.2916	4249.11230	242.83081	95.7565
2	11.277	MM	0.0640	50.74303	13.21964	1.1435
3	13.556	MM	0.1280	71.27004	9.27808	1.6061
4	14.651	MM	0.0838	66.28852	13.18128	1.4939

<u>*cyclo*-C(TAT)RLLIL</u>

Cyclo – CRLLIL (5 mg, 0.007 mmol, 1 eq) was added to a solution of C(Spy)GRKKRRQRRRPPQ (27 mg, 0.014, 2 eq) dissolved in DMF (2 mL) and stirred in an inert atmosphere overnight at ambient temperature. The mixture was then purified by reverse-phase HPLC. Fractions analysed via LC/MS, desired fractions were then pooled, concentrated *in vacuo*, and lyophilised to yield *Cyclo*-C(TAT)RLLIL as a white solid (12.5 mg, 0.0049 mmol, 71 %).

LR MS (ESI$^+$): m/z (M+6H)6+ calculated for $C_{106}H_{195}N_{45}O_{23}S_2$, 422.8, found; 423.1 $[M+6H]6^+$

HRMS (ESI$^+$): shows expected model isotope pattern.

Analytical HPLC Rt: (220 nm) 9.605min (100% Purity).

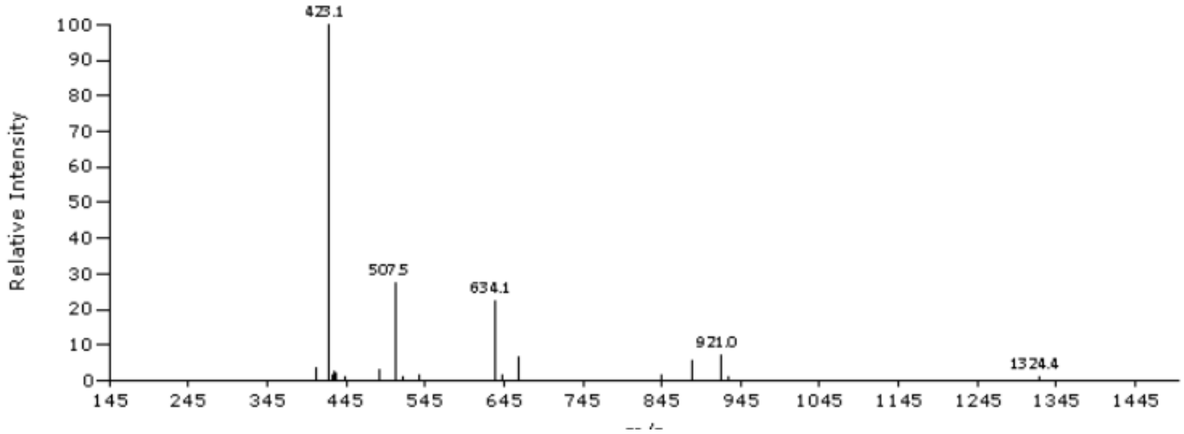

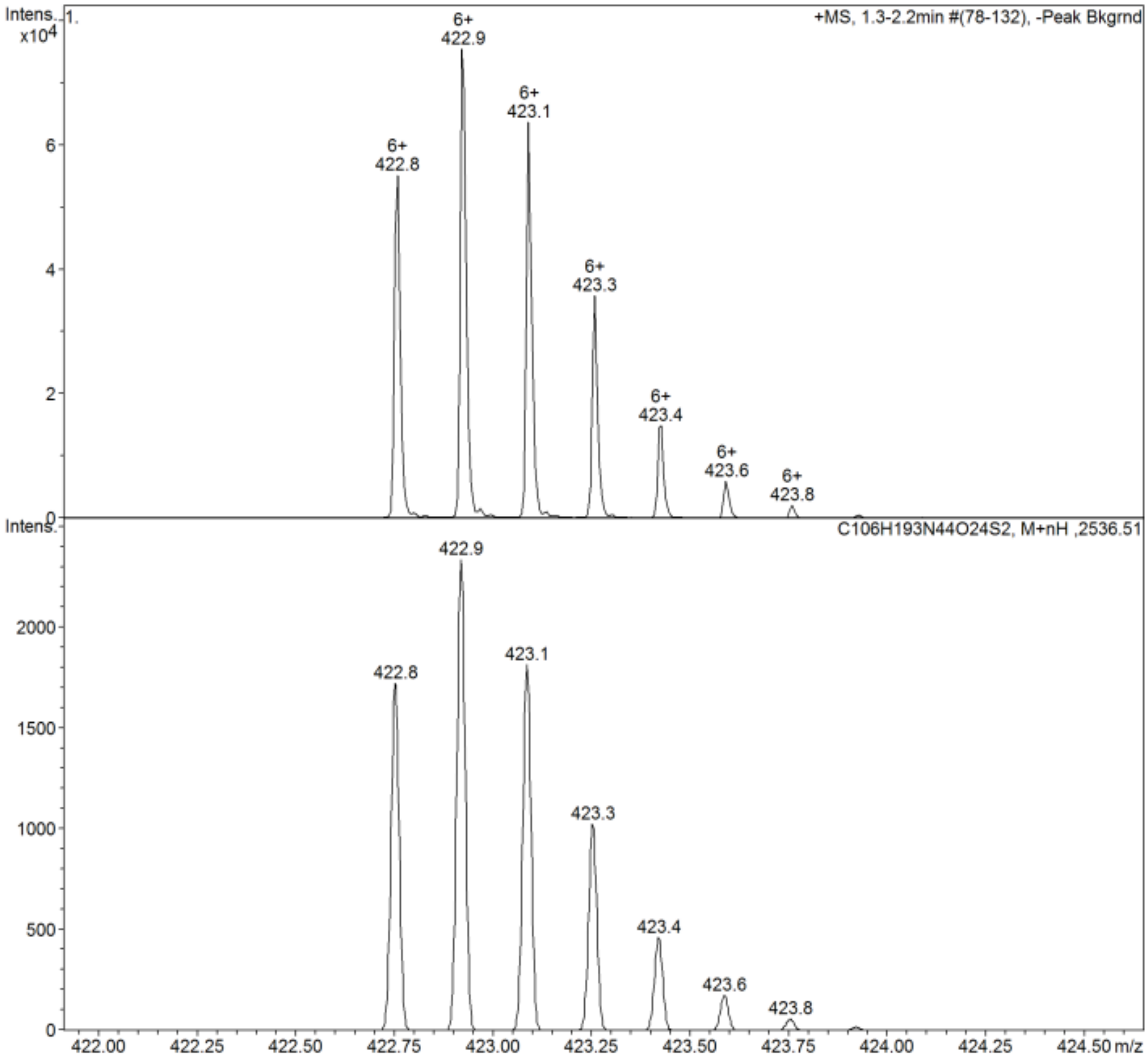

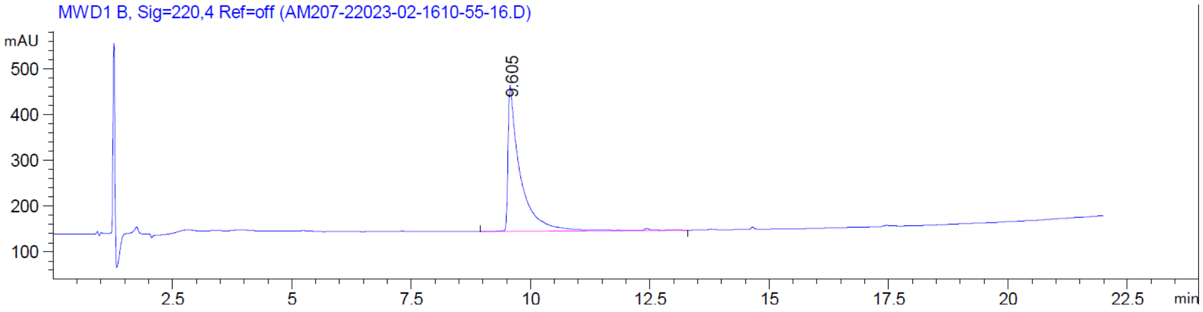

Peak #	RetTime [min]	Type	Width [min]	Area [mAU*s]	Height [mAU]	Area %
1	9.605	BV R	0.2843	5708.80420	281.91489	100.0000

cyclo-C(TAT)RRIIL

Cyclo – CRRIIL (5 mg, 0.0066 mmol, 1 eq) was added to a solution of C(Spy)GRKKRRQRRRPPQ (25 mg, 0.013 mmol, 2 eq) dissolved in DMF (2 mL) and stirred in an inert atmosphere overnight at ambient temperature. The mixture was then purified by reverse-phase HPLC. Fractions analysed via LC/MS, desired fractions were then pooled, concentrated *in vacuo*, and lyophilised to yield *Cyclo*-C(TAT)RLLIL as a white solid (10.5 mg, 0.0041 mmol, 62 %).

LR MS (ESI$^+$): m/z (M+6H)6+ calculated for $C_{106}H_{195}N_{45}O_{23}S_2$, 430.2, found; 430.2 $[M+6H]6^+$

HRMS (ESI$^+$): shows expected model isotope pattern.

Analytical HPLC Rt: (220 nm) 8.772 min (89.8% Purity).

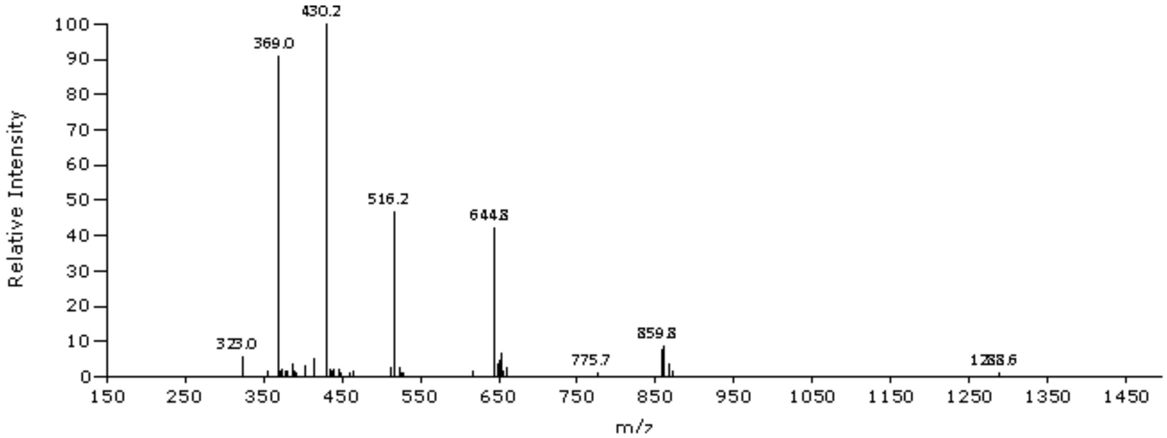

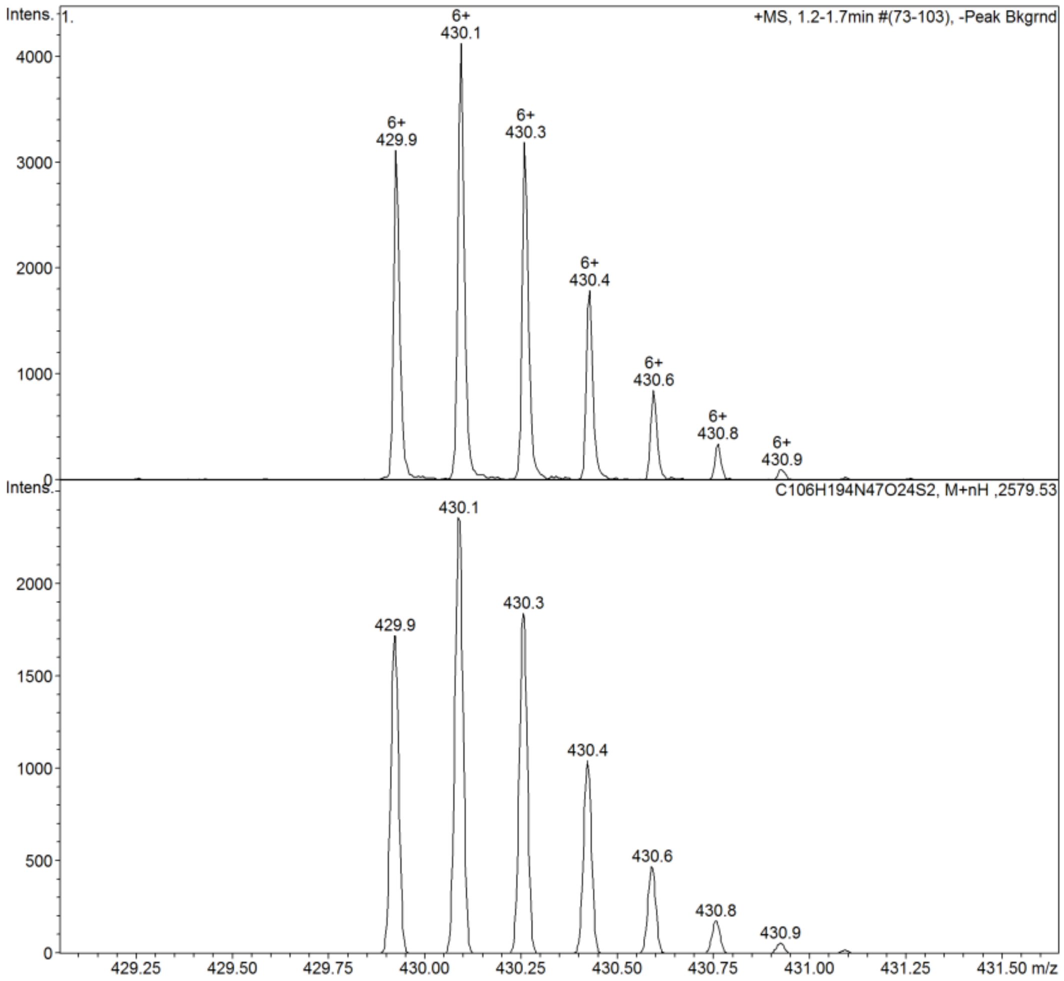

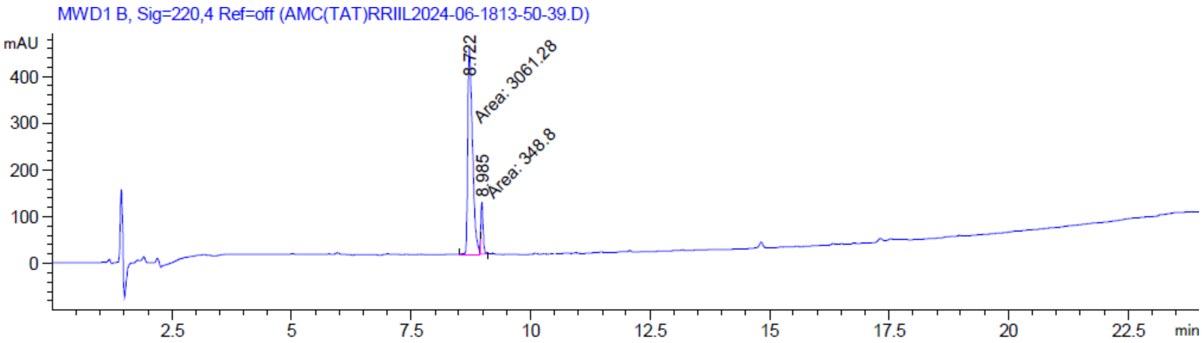

Peak #	RetTime [min]	Type	Width [min]	Area [mAU*s]	Height [mAU]	Area %
1	8.722	MM	0.1139	3061.27539	447.93839	89.7715
2	8.985	MM	0.0521	348.79999	111.64520	10.2285

<u>*cyclo*-C(TAT)RMFIL</u>

Cyclo – CRMFIL (5 mg, 0.006 mmol, 1 eq) was added to a solution of C(Spy)GRKKRRQRRRPPQ (23 mg, 0.012 mmol, 2 eq) dissolved in DMF (2 mL) and stirred in an inert atmosphere overnight at ambient temperature. The mixture was then purified by reverse-phase HPLC. Fractions analysed via LC/MS, desired fractions were then pooled, concentrated *in vacuo*, and lyophilised to yield *Cyclo*-C(TAT)RMFIL as a white solid (9 mg, 0.0035 mmol, 58 %).

LR MS (ESI$^+$): m/z (M+5H)5+ calculated for $C_{106}H_{195}N_{45}O_{23}S_2$, 517.4, found; 518.0 [M+5H]5$^+$

HRMS (ESI$^+$): shows expected model isotope pattern.

Analytical HPLC Rt: (220 nm) 9.613 min (97.5% Purity).

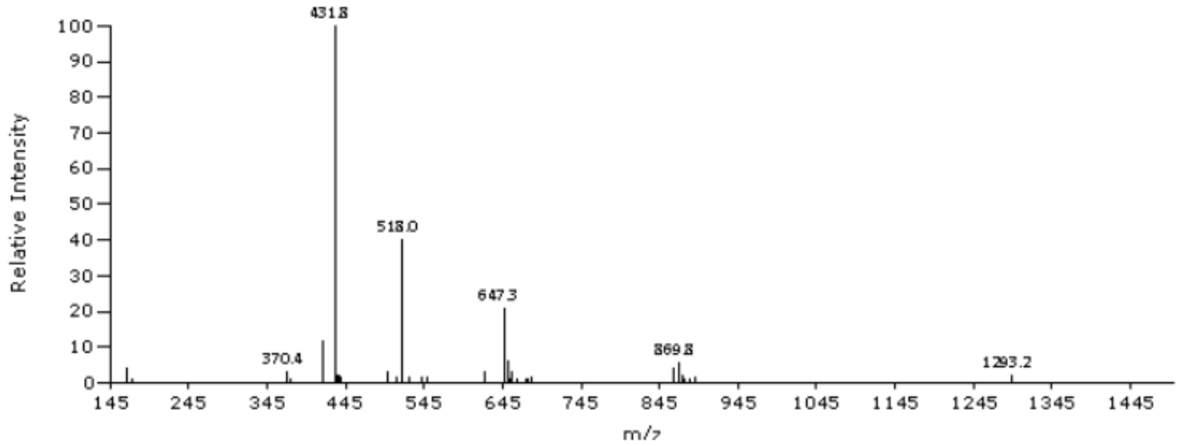

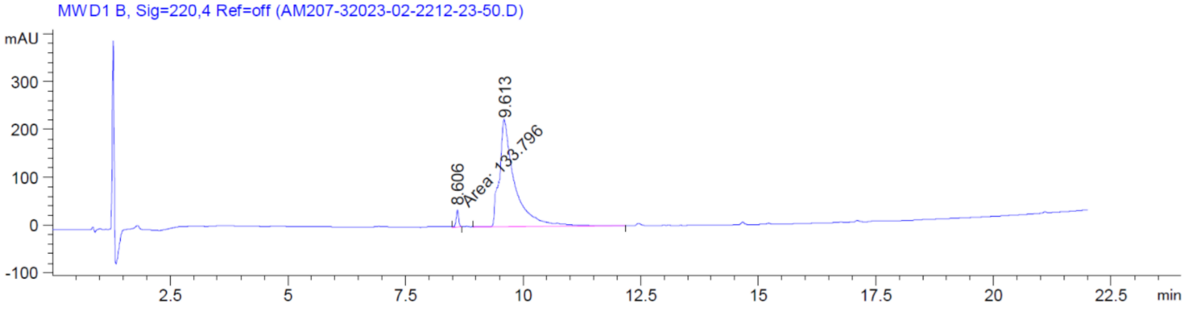

Peak #	RetTime [min]	Type	Width [min]	Area [mAU*s]	Height [mAU]	Area %
1	8.606	MM	0.0618	133.79616	36.10934	2.4993
2	9.613	VB	0.3279	5219.61084	217.59705	97.5007

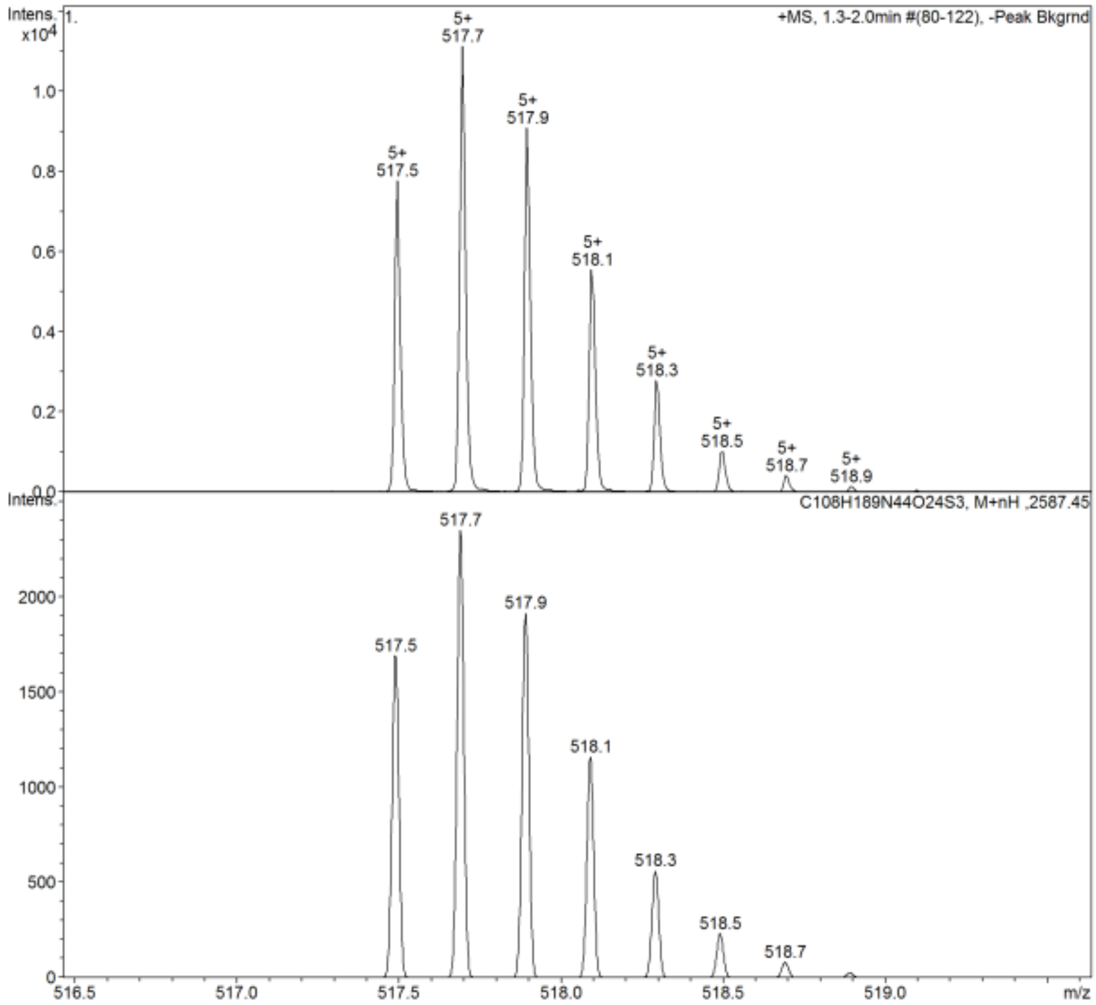
Intens.
x10^4
1.
+MS, 1.3-2.0min #(80-122), -Peak Bkgrnd
5+ 517.5
5+ 517.7
5+ 517.9
5+ 518.1
5+ 518.3
5+ 518.5
5+ 518.7
5+ 518.9
C108H189N44O24S3, M+nH ,2587.45
517.5
517.7
517.9
518.1
518.3
518.5
518.7
m/z

cyclo-C(TAT)RYLIL

Cyclo – CRYLIL (4 mg, 0.0053 mmol, 1 eq) was added to a solution of C(Spy)GRKKRRQRRRPPQ (21 mg, 0.011 mmol, 2 eq) dissolved in DMF (2 mL) and stirred in an inert atmosphere overnight at ambient temperature. The mixture was then purified by reverse-phase HPLC. Fractions analysed via LC/MS, desired fractions were then pooled, concentrated *in vacuo*, and lyophilised to yield *Cyclo*-C(TAT)RYLIL as a white solid (10 mg, 0.0039 mmol, 74 %).

LR MS (ESI$^+$): m/z (M+5H$^+$) calculated for $C_{109}H_{193}N_{45}O_{24}S_2$, 517.1, found; 517.3.

HRMS (ESI$^+$) shows expected model isotope pattern.

Analytical HPLC Rt: (220 nm) 8.986 min (99% Purity).

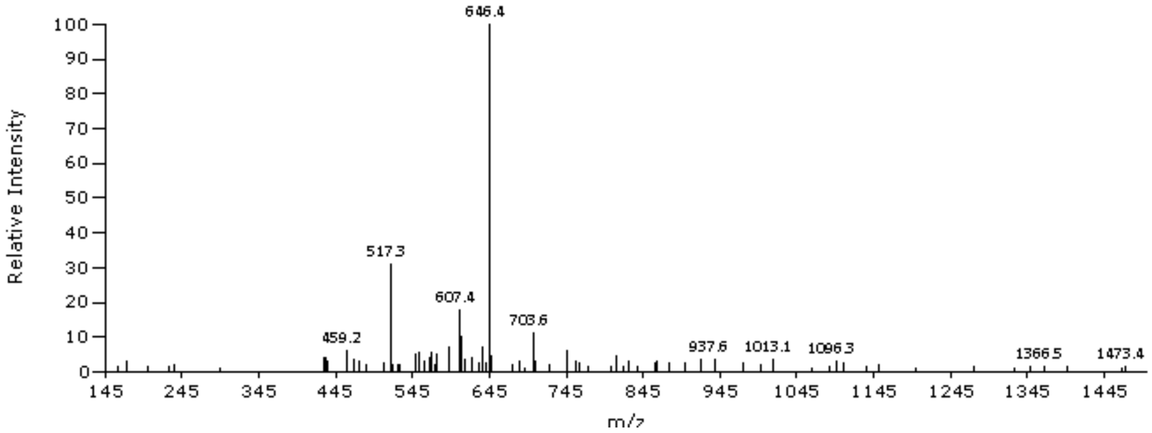

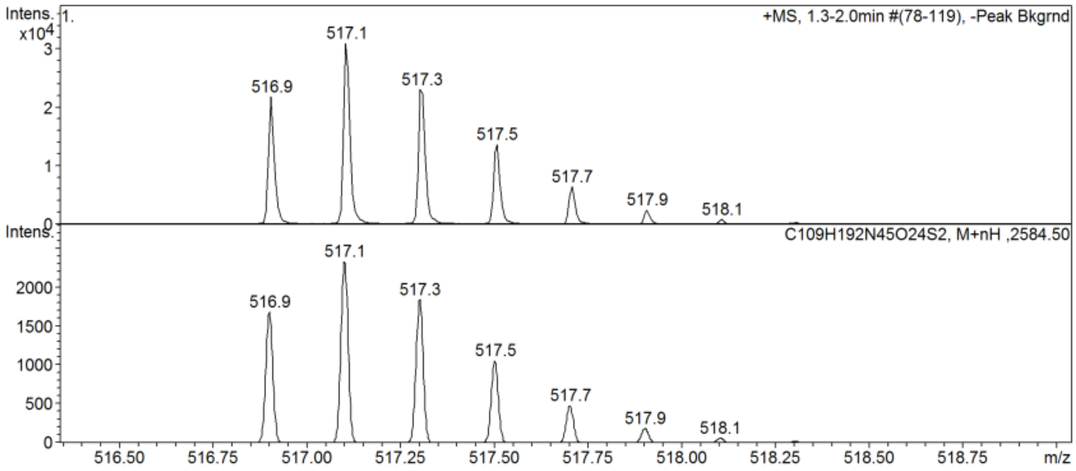

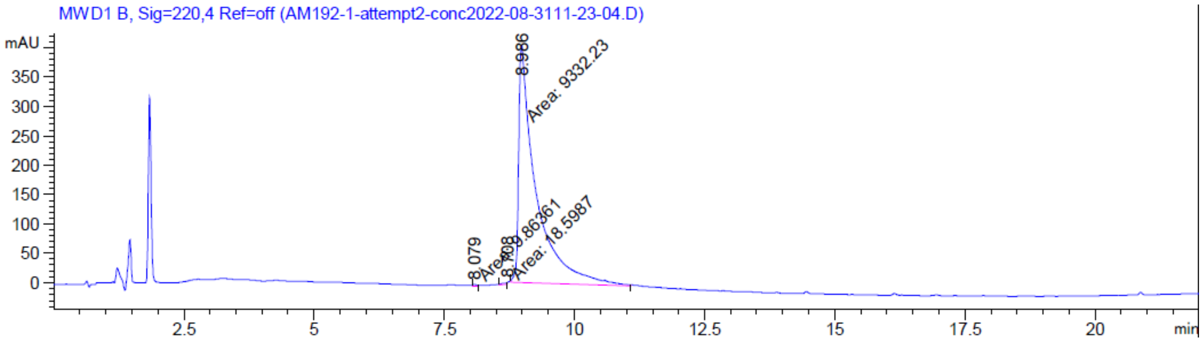

Peak #	RetTime [min]	Type	Width [min]	Area [mAU*s]	Height [mAU]	Area %
1	8.079	MM	0.0588	9.86361	2.79711	0.1054
2	8.708	MM	0.0941	18.59869	3.29305	0.1987
3	8.986	MM	0.3851	9332.22852	403.83920	99.6959

<u>*cyclo*-C(TAT)LLFC(TAT)L</u>

Cyclo – CLLFCL (5 mg, 0.0072 mmol, 1 eq) was added to a solution of C(Spy)GRKKRRQRRRPPQ (41.2 mg, 0.021 mmol, 3 eq) dissolved in DMF (2 mL) and stirred in an inert atmosphere overnight at ambient temperature. The mixture was then purified by reverse-phase HPLC. Fractions analysed via LC/MS, desired fractions were then pooled, concentrated *in vacuo*, and lyophilised to yield *Cyclo*-C(TAT)LLFC(TAT)L as a white solid (11 mg, 0.0025 mmol, 35 %).

LR MS (ESI$^+$): m/z (M+8H$^+$) calculated for $C_{179}H_{320}N_{78}O_{40}S_4$, 542.3, found; 542.8.

HRMS (ESI$^+$) shows expected model isotope pattern.

Analytical HPLC Rt: (220 nm) 8.363 min (94% Purity).

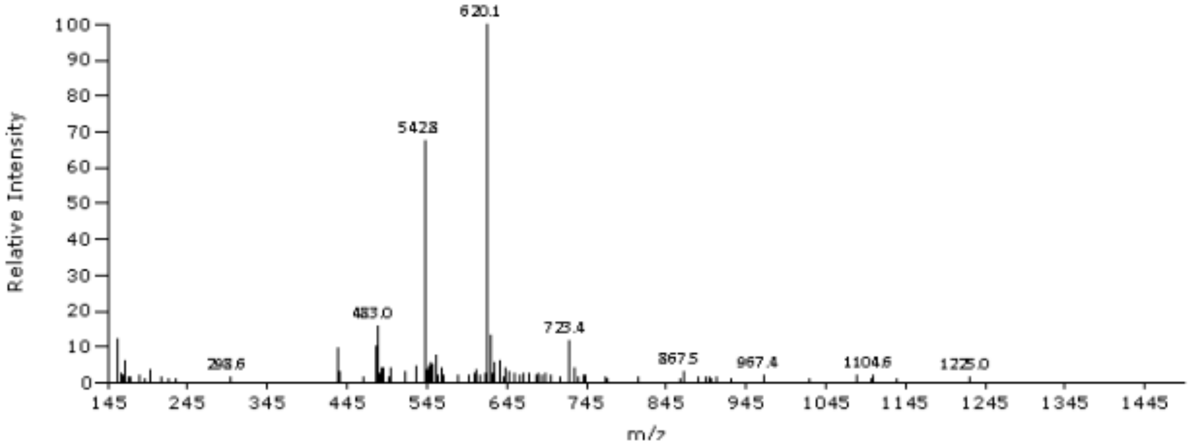

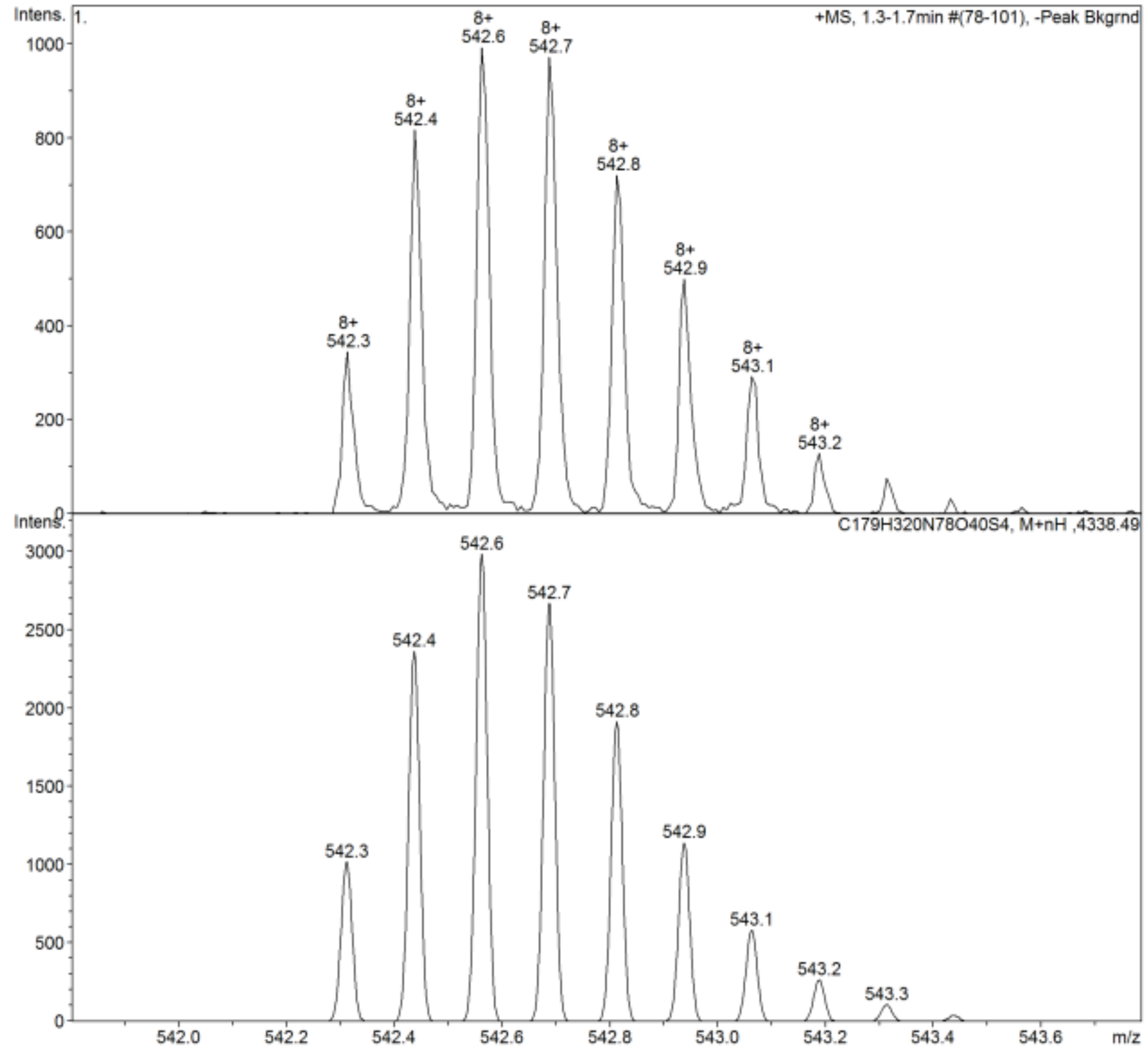

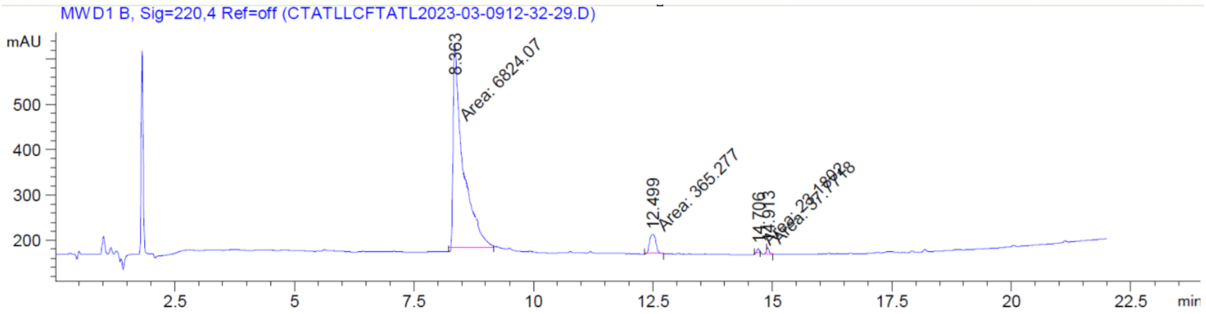

Peak #	RetTime [min]	Type	Width [min]	Area [mAU*s]	Height [mAU]	Area %
1	8.363	MM	0.2511	6824.07080	453.00864	94.1212
2	12.499	MM	0.1479	365.27682	41.15342	5.0381
3	14.706	MM	0.0523	23.18016	7.38468	0.3197
4	14.913	MM	0.0429	37.77177	14.66503	0.5210

<u>*cyclo*-C(TAT)LLFVY</u>

Cyclo – CLLFVY (5.8 mg, 0.0079 mmol, 1 eq) was added to a solution of C(Spy)GRKKRRQRRRPPQ (30.5 mg, 0.0158 mmol, 2 eq) dissolved in DMF (2 mL) and stirred in an inert atmosphere overnight at ambient temperature. The mixture was then purified by reverse-phase HPLC. Fractions analysed via LC/MS, desired fractions were then pooled, concentrated *in vacuo*, and lyophilised to yield *Cyclo*-C(TAT)LLFC(TAT)L as a white solid (11 mg, 0.0025 mmol, 35 %).

LR MS (ESI$^+$): m/z (M+4H$^+$) calculated for $C_{111}H_{188}N_{42}O_{24}S_2$, 640.4, found; 640.7.

HRMS (ESI$^+$) shows expected model isotope pattern.

Analytical HPLC Rt: (220 nm) 8.363 min (94.7% Purity).

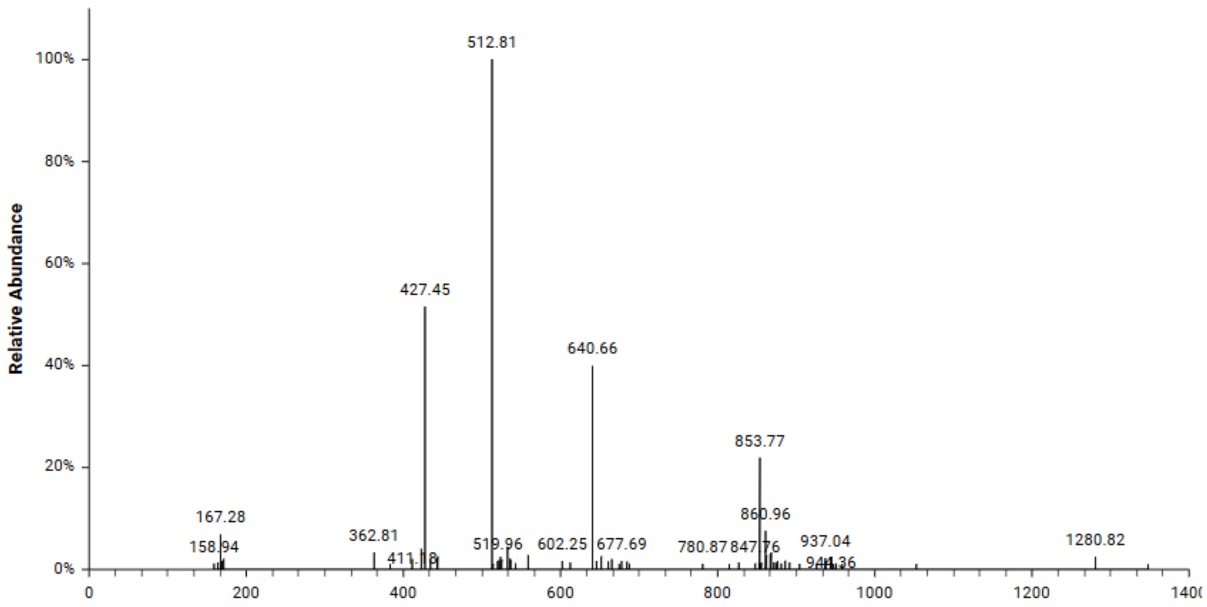

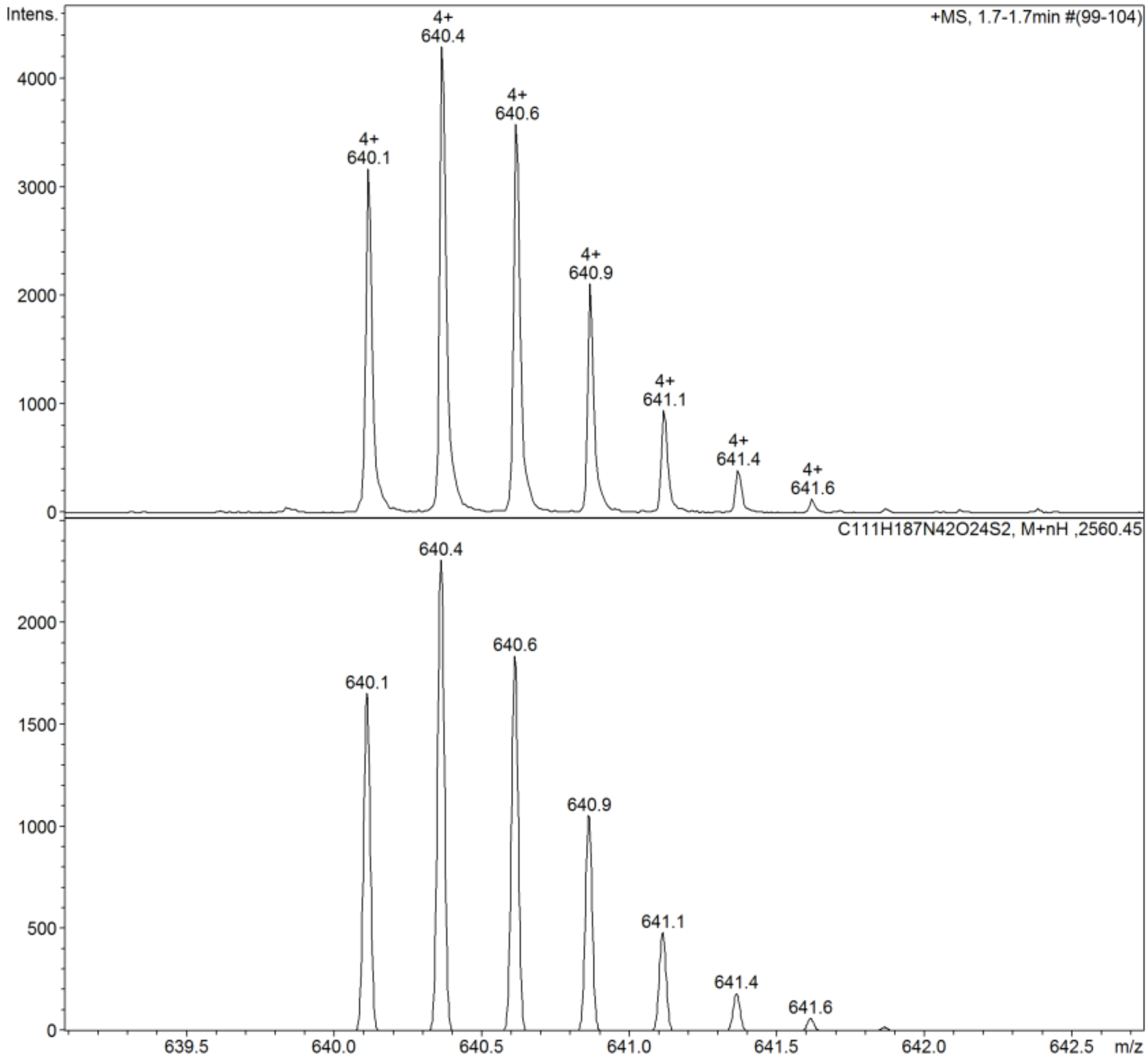

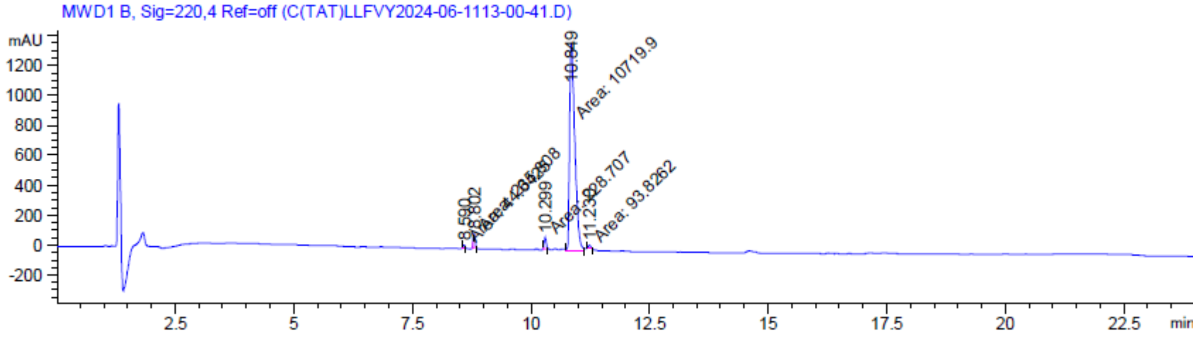

Peak #	RetTime [min]	Type	Width [min]	Area [mAU*s]	Height [mAU]	Area %
1	8.590	MM	0.0362	44.64246	20.57647	0.3943
2	8.802	MM	0.0413	235.80789	95.06930	2.0826
3	10.299	MM	0.0492	228.70667	77.53615	2.0199
4	10.849	MM	0.1283	1.07199e4	1392.58667	94.6746
5	11.230	MM	0.0689	93.82619	22.70897	0.8286

References

(1) Henikoff, S.; Henikoff, J. G. Amino acid substitution matrices from protein blocks. *Proc Natl Acad Sci U S A* **1992**, *89* (22), 10915-10919. DOI: 10.1073/pnas.89.22.10915 From NLM Medline.
(2) Miranda, E.; Nordgren, I. K.; Male, A. L.; Lawrence, C. E.; Hoakwie, F.; Cuda, F.; Court, W.; Fox, K. R.; Townsend, P. A.; Packham, G. K.; et al. A cyclic peptide inhibitor of HIF-1 heterodimerization that inhibits hypoxia signaling in cancer cells. *J Am Chem Soc* **2013**, *135* (28), 10418-10425. DOI: 10.1021/ja402993u From NLM Medline.
(3) Tavassoli, A.; Benkovic, S. J. Split-intein mediated circular ligation used in the synthesis of cyclic peptide libraries in E. coli. *Nat Protoc* **2007**, *2* (5), 1126-1133. DOI: 10.1038/nprot.2007.152 From NLM Medline.
(4) Cock, P. J.; Antao, T.; Chang, J. T.; Chapman, B. A.; Cox, C. J.; Dalke, A.; Friedberg, I.; Hamelryck, T.; Kauff, F.; Wilczynski, B.; et al. Biopython: freely available Python tools for computational molecular biology and bioinformatics. *Bioinformatics* **2009**, *25* (11), 1422-1423. DOI: 10.1093/bioinformatics/btp163 From NLM Medline.
(5) Tareen, A.; Kinney, J. B. Logomaker: beautiful sequence logos in Python. *Bioinformatics* **2020**, *36* (7), 2272-2274. DOI: 10.1093/bioinformatics/btz921 From NLM Medline.
(6) Pedregosa, F.; Varoquaux, G.; Gramfort, A.; Michel, V.; Thirion, B.; Grisel, O.; Blondel, M.; Prettenhofer, P.; Weiss, R.; Dubourg, V.; et al. Scikit-learn: Machine Learning in Python. *J Mach Learn Res* **2011**, *12*, 2825-2830.
(7) Ball, A. T.; Mohammed, S.; Doigneaux, C.; Gardner, R. M.; Easton, J. W.; Turner, S.; Essex, J. W.; Pairaudeau, G.; Tavassoli, A. Identification and Development of Cyclic Peptide Inhibitors of Hypoxia Inducible Factors 1 and 2 That Disrupt Hypoxia-Response Signaling in Cancer Cells. *J Am Chem Soc* **2024**, *146* (13), 8877-8886. DOI: 10.1021/jacs.3c10508 From NLM Medline.
